# Overdeepenings in the Swiss plateau: U-shaped geometries underlain by inner gorges

**DOI:** 10.1186/s00015-023-00447-y

**Published:** 2023-12-05

**Authors:** Dimitri Bandou, Fritz Schlunegger, Edi Kissling, Urs Marti, Regina Reber, Jonathan Pfander

**Affiliations:** 1 Institute of Geological Sciences, University of Bern, Bern, Switzerland; 2 Department of Earth Sciences, ETH Zurich, Zurich, Switzerland; 3 Landesgeologie Swisstopo, Bern, Switzerland

## Abstract

We investigated the mechanisms leading to the formation of tunnel valleys in the Swiss foreland near Bern. We proceeded through producing 3D maps of the bedrock topography based on drillhole information and a new gravimetric survey combined with modelling. In this context, the combination of information about the densities of the sedimentary fill and of the bedrock, together with published borehole data and the results of gravity surveys along 11 profiles across the valleys, served as input for the application of our 3D gravity modelling software referred to as PRISMA. This ultimately allowed us to model the gravity effect of the Quaternary fill of the overdeepenings and to produce cross-sectional geometries of these troughs. The results show that 2–3 km upstream of the city of Bern, the overdeepenings are approximately 3 km wide. They are characterized by steep to oversteepened lateral flanks and a wide flat base, which we consider as a U-shaped cross-sectional geometry. There, the maximum residual gravity anomaly ranges between − 3 to − 4 mGal for the Aare valley, which is the main overdeepening of the region. Modelling shows that this corresponds to a depression, which reaches a depth of c. 300 m a.s.l. Farther downstream approaching Bern, the erosional trough narrows by c. 1 km, and the base gets shallower by c. 100 m as revealed by drillings. This is supported by the results of our gravity surveys, which disclose a lower maximum gravity effect of c. − 0.8 to − 1.3 mGal. Interestingly, in the Bern city area, these shallow troughs with maximum gravity anomalies ranging from − 1.4 to − 1.8 mGal are underlain by one or multiple inner gorges, which are at least 100 m deep (based on drilling information) and only a few tens of meters wide (disclosed by gravity modelling). At the downstream end of the Bern area, we observe that the trough widens from 2 km at the northern border of Bern to c. 4 km approximately 2 km farther downstream, while the bottom still reaches c. 300 to 200 m a.s.l. Our gravity survey implies that this change is associated with an increase in the maximum residual anomaly, reaching values of − 2.5 mGal. Interestingly, the overdeepening’s cross-sectional geometry in this area has steeply dipping flanks converging to a narrow base, which we consider as V-shaped. We attribute this shape to erosion by water either underneath or at the snout of a glacier, forming a gorge. This narrow bedrock depression was subsequently widened by glacial carving. In this context, strong glacial erosion upstream of the Bern area appears to have overprinted these traces. In contrast, beneath the city of Bern and farther downstream these V-shaped features have been preserved. Available chronological data suggest that the formation of this gorge occurred prior to MIS 8 and possibly during the aftermath of one of the largest glaciations when large fluxes of meltwater resulted in the fluvial carving into the bedrock.

**Supplementary Information:**

The online version contains supplementary material available at 10.1186/s00015-023-00447-y.

## Introduction

### Overdeepenings

Overdeepenings are bedrock depressions with a thalweg that is below the base level in the region. Such erosional features have been observed in mountainous areas and their lowlands that have experienced one or multiple glaciations (Preusser et al., 2010, 2011; Fischer and Häberli, 2012; Linsbauer et al., 2016; Häberli et al., 2016). Overdeepenings, however, have also been reported from flat areas such as the Midwest of North America (Wright, 1973), northern continental Europe (e.g., Piotrowski, 1997; Krohn et al., 2009), Scandinavia (Clark and Walder, 1994), the North Sea (Moreau et al., 2012; Lohrberg et al., 2022), and beneath the Greenland and Antarctic ice sheets (Patton et al., 2016). Most authors have considered a formation beneath a glacier (e.g., Wright, 1973; Schlüchter, 1989; Preusser et al., 2011; Kehew et al., 2012; Dürst Stucki and Schlunegger, 2013; Liebl et al., 2023; Kirkham et al., 2021; 2023), mainly because these depressions occur below the regional base level, are closed depressions, and are situated in mountainous regions far from the influence of sea-level fluctuations. Although overdeepenings or tunnel valleys have been reported from a large variety of settings, interpretations of the processes through which they were carved are still being contested (e.g., Cook and Swift, 2012; Kirkham et al., 2022). This is the case because the analysis of the overdeepenings’ shapes, which potentially contain diagnostic information for interpreting the related erosional processes (Magrani et al., 2020; Gegg and Preusser, 2023), is thwarted as these troughs are buried by sediments. Therefore, the geometries of these troughs have to be determined indirectly by geophysical surveys or through drillings. Nevertheless, interpretations range from the view where overdeepenings were carved by glacial processes with support by englacial and subglacial meltwater (Egholm et al., 2009; 2012; Herman and Braun, 2008; Herman et al., 2011; Beaud et al., 2014; Liebl et al., 2023) particularly for cases where they have a U-shaped cross-sectional geometry characterized by a flat base and steep lateral flanks (Kehew et al., 2012). Alternative interpretations point to the importance of overpressurized subglacial meltwater as erosional process, where a continuous (Smed, 1998; Huuse and Lykke-Andersen, 2000; Praeg, 2003; Cohen et al., 2023) or episodic outburst of water from beneath the snout of glaciers (Wright, 1973; Piotrowski, 1994; Björnsson, 1996; Clayton et al., 1999; Beaney, 2002; Shaw, 2002; Jørgensen and Sandersen, 2006) could contribute to the carving into the bedrock. The result is a cross-sectional geometry that is V-shaped, where the flanks are steep and converge at depth to a narrow, approximately < 20 m wide base. The meltwater origin hypothesis bases on Bernoulli’s principle (e.g., Batchelor, 1967), where at the glacier’s snout a decrease in the ice thickness leads to large drops in hydrostatic pressures, which translates into hydrodynamic pressures and thus into erosional work, under the condition that the subglacial channel network is a closed system. In either cases, most studies converge to the notion where glacial carving yields tunnel valleys with U-shaped cross-sectional geometries, whereas erosion through overpressurized subglacial meltwater preferentially returns V-shaped incisions.

### Overdeepenings in the Swiss plateau and aim of paper

The Swiss Plateau, which is located on the northern side of the Alps, hosts several well-studied tunnel valleys and overdeepenings (Moscariello et al., 1998; Preusser et al., 2010; Horstmeyer et al., 2012; Dürst Stucki and Schlunegger, 2013; Schnellmann and Madrisch, 2014; Magrani et al., 2020; Anselmetti et al., 2022; Gegg and Preusser, 2023), which have been analysed through seismic and gravity surveys and sedimentary archives encountered in drillings (Kissling and Schwendener, 1990; Rosselli and Raymond, 2003; Dehnert et al., 2012, Buechi et al., 2017a, b; Jordan, 2010; Dürst Stucki et al., 2010; Schnellman and Madritsch, 2014; Reber and Schlunegger, 2016; Gegg et al., 2021; Schwenk et al., 2022a, 2022b; Bandou et al., 2022). In this context, three hypotheses have been proposed to explain the origin of these troughs in the Alpine region. As a first mechanism, it was considered that fluvial erosion could have caused the formation of narrow gorges, which were subsequently widened by glacial carving (Kissling and Schwendener, 1990). As a second origin, it has been proposed that the tunnel valleys were carved with support by overpressurized meltwater (Dürst Stucki and Schlunegger, 2013; Cohen et al., 2023). However, as noted above, this mechanism requires a hydrologically closed system, which might be challenged by the relatively large permeabilities usually measured for the Molasse bedrock (Keller et al., 1990) that underlies these troughs, at least in the Swiss Plateau. Finally, glacial carving through bedrock abrasion or quarrying could offer a third mechanisms to explain the formation of these troughs (Herman et al., 2011, Sternai et al., 2013). The resulting erosional shapes are usually U-shaped, because the glaciers’ viscosities result in the translation of some of the vertical pressure gradients towards the lateral sides through dislocation creep, thereby widening the incisions (Cuffey and Paterson, 2010; Alley et al., 2019).

In this contribution, we aim at assessing the erosional processes resulting in the formation of the overdeepenings with a focus on the system in the Bern area situated on the northern margin of the Swiss Alps. Admittedly, the geometry of the overdeepenings in the Bern region has already been reconstructed by Reber and Schlunegger (2016) at a high level of details based on thousands of drillings. Yet due to a lack of deep drillings in the center of the channels, the accuracy of the existing bedrock topography model is limited particularly for the deeper levels. Therefore, given the success in applying gravity surveys for disclosing the geometry of overdeepenings in previous contributions (Kissling and Schwendener, 1990; Rosselli and Raymond, 2003; Perrouty et al., 2016; Bandou et al., 2022), we collected additional gravity data from the overdeepening system in the Bern area to retrieve more information particularly on the geometry of the lateral flanks and the basal parts of this trough. We then complemented the results of our gravity survey with a synthesis on existing chronological data about the valley fill to reconstruct the history of overdeepening formation in this area, and to infer the mechanisms through which these troughs were formed.

## Setting

**Fig. 1:**
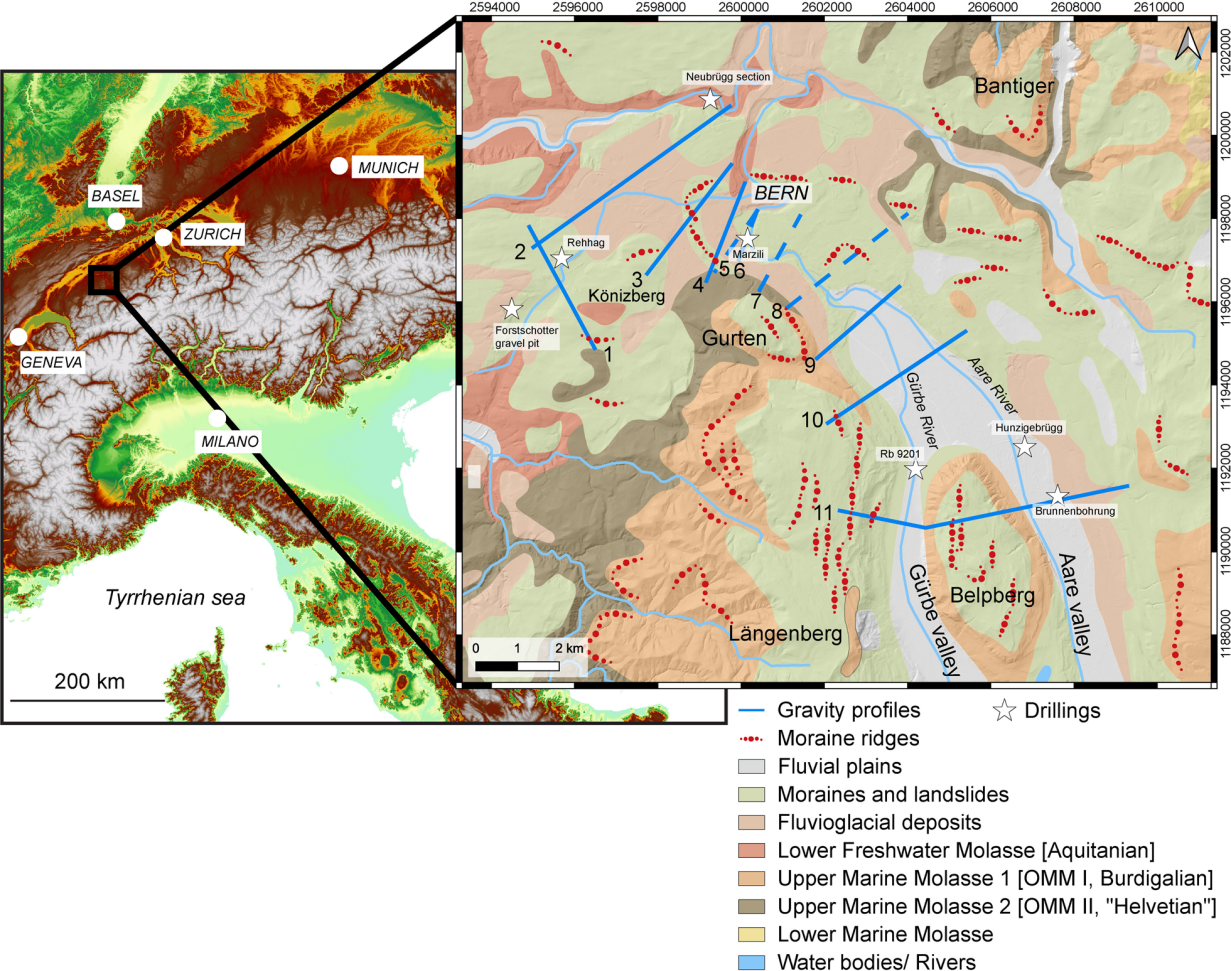
Geological map taken from Swisstopo (Gerber, 1927), showing the pattern of the surface geology and the location of the drillings and sections (stars on the figure), which provided key information for this work. These are the Brunnenbohrung Münsingen (Kellerhals and Häfeli, 1984), the Rehhag Drilling (Schwenk et al., 2022a, b) and the Neubrügg section (Lüthy et al. (1963), for which limited information about the chronological framework for the Quaternary sediments is available. We also used constraints offered by the Forstschotter gravel pit, the drilling RB 9201 (Kellerhals and Isler, 1983; Geotest, 1995) and that of the Hunzigebrügg (Zwahlen et al., 2021). Modified after Beck and Rutsch (1949), Spicher (1972) and Bandou et al. (2022). The map also shows the various sections we analysed through a gravimetric survey only (dashed blue lines) and through a combination of a gravity survey and modelling (blue lines). These are the following profiles: (1) Bümpliz, (2) Bremgarten, (3) Bern1, (4) Bern2, (5) Bern3, (6) Bern4, (7) Wabern1, (8) Wabern2, (9) Kehrsatz and (10) Airport. The Gürbe valley—Belpberg—Aare valley profile (11) was already published in a previous contribution (Bandou et al., 2022)

**Fig. 2:**
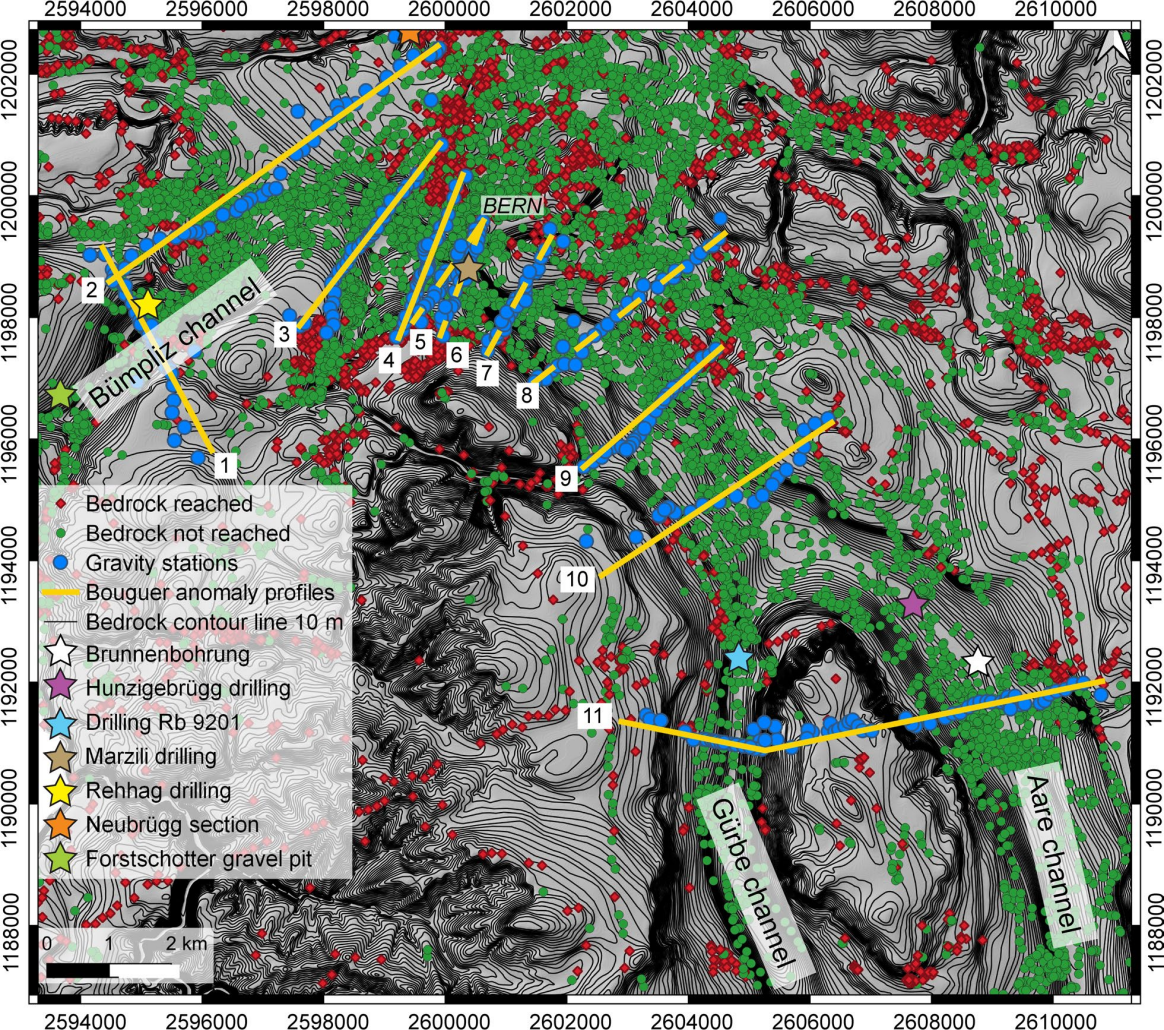
Map showing the bedrock topography of the Bern area with 10 m-contour lines. The map was reproduced using the openly accessible digital dataset for the bedrock topography of the canton Bern (https://www.geo.apps.be.ch/de/;Karten;Felsrelief), and it is also available from the openly accessible database of swisstopo (© swisstopo). This dataset was originally produced by Reber and Schlunegger (2016). The map also displays the drillings that reached the bedrock (red dots). Most of the drillings, however, did not reach the bedrock and yield minimum constraints on the depths of the overdeepening contour lines (green dots). The blue dots illustrate the location of the gravity stations that are used in this study. The map also shows the profiles (in yellow) that were analyzed for the measured gravity signals (see legend of Fig. 1 for labels). Modified after Bandou et al. (2022)

The overdeepening system of the Bern area (Fig. 1), which is the focus of the study, was previously uncovered by thousands of shallow drillings that were sunk into the subsurface for engineering purposes (Reber and Schlunegger, 2016; Fig. 2). This resulted in a level of details that has been unprecedented so far for an overdeepening system. The available bedrock topography map (Reber and Schlunegger, 2016) shows that in the upstream area south of Bern, the tunnel valley system consists of two overdeepenings referred to as the Gürbe and the Aare overdeepening (Fig. 2). They are separated by a bedrock ridge made up of Early Miocene Upper Molasse sandstones (Fig. 1). These two troughs, which are c. 1.5 km (Gürbe) and approximately 2.5 km-wide (Aare overdeepening or Aare channel) and c. 160 m (Gürbe) and 270 m deep (Aare), converge c. 10 km south of Bern to one single depression that is c. 3 km wide and that strikes SE-NW (Figs. 1 and 2). This single depression, which is still > 1 km wide beneath Bern, transitions into a wider basin with side channels striking SW-NE that connect to the main basin c. 2 km NW of Bern (Reber and Schlunegger, 2016; see Fig. 2). The largest of these side troughs was referred as the Bümpliz channel (Fig. 2) and was explored through the Rehhag scientific drilling (Figs. 1, 2) by Schwenk et al., (2022a, b). This drilling reached the bedrock at 362 m a.s.l. (Schwenk et al., 2022a), which corresponds to a drilling depth of 210 m.

**Fig. 3:**
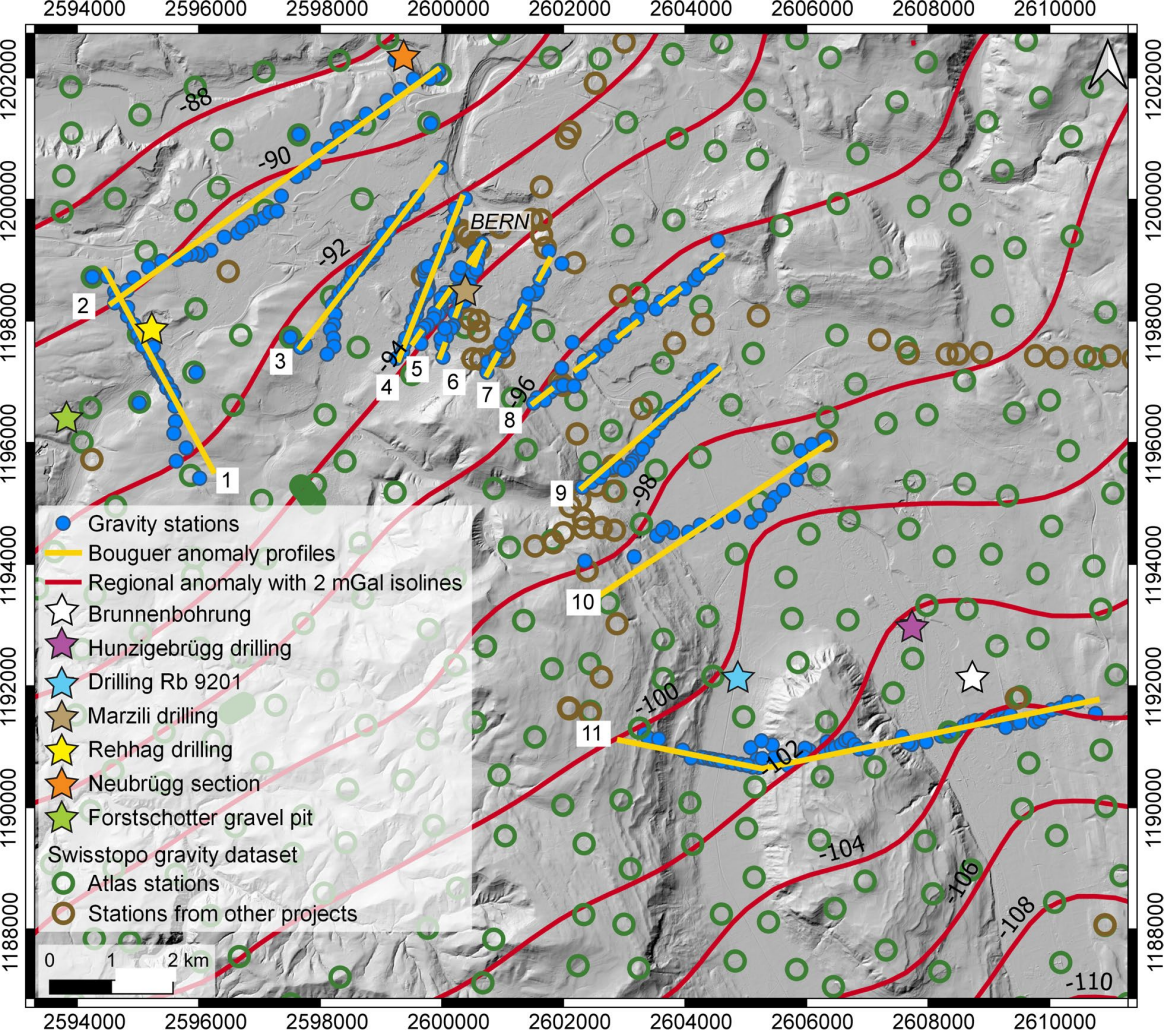
Modern topography from the SwissAlti 3D DEM ( © swisstopo) with gravity profiles and gravity data from the Gravity Atlas of Switzerland, modified from Bandou et al. (2022). The map also shows the Bouguer anomaly contour lines and the locations of our stations (blue dots) and those of key drillings (stars). The map and the contour lines are from the openly accessible database of swisstopo (‘Carte gravimétrique de la Suisse (Anomalies de Bouguer) 1: 500,000, © swisstopo; Olivier et al., 2008; 2011). The yellow line shows the locations of our profiles (yellow lines, see legend of Fig. 1 for explanation of labels). The gravity values at the green and brown stations are available from the swisstopo database. The data of the brown stations were collected in the framework of various projects, and they are now integrated in the Gravity Atlas of Switzerland. Note that the contour lines were derived from the green stations only

Age constraints on the overdeepening fill are provided by three sections. These are the deposits encountered in the Aare overdeepening (Brunnenbohrung Münsingen, Fig. 1) providing an age between MIS 6 and 2 (Kellerhals and Isler, 1983; Kellerhals and Häfeli, 1984; Zwahlen et al., 2021; Bandou et al., 2022). Another age constraint was established for a temporary outcrop on the downstream end of the main overdeepening where a cliff on the SW margin of the Aare River (Neubrügg section, Fig. 1) exposes a suite of Quaternary sediments. This succession consists of a till at the base and the top of the section, which were referred to as ‘Riss’ and ‘Würm’ moraines by Lüthy et al. (1963). The succession also includes a sand and a gravel layer with pollen fragments in the sand layer. Lüthy et al. (1963) did not assign a precise age to this latter unit, but they considered that the pollen fragments (mainly from spruce and beech) could record the end of a warm period (end of MIS 5e?). Recently, age constraints were presented by Schwenk et al. (2022a) for the Quaternary infill of the Bümpliz tributary trough (Fig. 1; Rehhag drilling) based on optically stimulated luminesce (OSL) data measured for quartz minerals (Schwenk, 2022, Schwenk et al., 2022a). This information points towards a minimum age of MIS 8 approximately between 300′000 and 250′000 years before present. To the SW of the Rehhag drilling, a thick gravel referred to as Forstschotter (Figs. 1, 2 and 3), forms a cap unit, which was tentatively assigned to the MIS 6 by Schwenk et al. (2022a) based on its morphostratigraphic and lithostratigraphic position.

South of the Bern region the bedrock comprises several 100 m-thick packages of Late Miocene sandstones, which have been referred to as the Upper Marine Molasse (OMM; German abbreviation) in the regional literature (Beck and Rutsch, 1949) and which also constitute an important sediment source for the overdeepening fill (Schwenk et al., 2022b). In the area of the city of Bern and farther north, the bedrock consists of an alternation of sandstones and mudstones. These deposits are part of the Lower Freshwater Molasse (USM; German abbreviation) and underlie the OMM (Isenschmid, 2019). In the city of Bern, the Molasse bedrock is dissected by transtensional NW–SE striking faults (Isenschmid, 2019).

## Methods

Following Kissling and Schwendener (1990) and Bandou et al. (2022) we determined the change of the shape of the overdeepenings along a series of profiles where gravity data was acquired in the field (Sect. 3.1), because such information is most diagnostic for the erosional mechanism at work (Preusser et al., 2011; Magrani et al., 2020). In this context, the reconstruction of the cross-sectional bedrock topography requires that for each profile, the Bouguer gravity values (Sect. 3.2) and particularly the gravity contribution of the overdeepening fill (the so-called residual anomaly values, Sect. 3.3) will be determined. Such information is then used to determine the wavelength of the main trough, and it will be employed to identify the gravity effect of secondary overdeepenings and/or geological structures either at the lateral flanks of the profiles or beneath them. As a subsequent step, we used a forward modelling approach to compute the gravity effect (Sect. 3.4) of the overdeepening fill thereby approximating the geometry of these bedrock troughs by prisms (Kissling and Schwendener, 1992; Bandou et al., 2022). The modelling and the subsequent visualization of the modelling results requires that coordinates have to be transformed from the Swiss to a local coordinate system (LCS, Sect. 3.3).

### Collection of gravity data in the field

We collected gravity data along 10 new sections (Figs. 1, 2), which are between 2 and 6 km long and have a spacing that ranges between 2 and 1 km. The gravity data of Sect. 11 (Fig. 2) was already processed and discussed by Bandou et al. (2022), the results of which we include in this paper. We particularly place our sections where the lateral boundary of the target overdeepening is constrained by shallow Molasse bedrock (< 5 m according to Reber and Schlunegger, 2016) on either profile end and where thus no significant gravity effect of the overdeepening fill is expected. Upon collecting gravity data, we selected a spacing between the new MPs (measurement points) that ranges from 50 to 200 m. Yet some MPs were separated by a longer distance (c. 400 to 600 m) particularly where the terrain (e.g., dense forest, cliffs, private properties, train stations, tracks, highways etc.) precludes a more densely-spaced survey. For each site, we locally selected a point where the coordinates are placed exactly between the cells of the 2 m SwissAlti3D DEM (Digital Elevation Model; see Fig. 3). This allowed us to measure the elevation in the middle of the four cells surrounding the station (Bandou, 2023a). At these sites, we measured the GNSS coordinates including the elevations that were taken in the Swiss LV95 system. This was done to best account for the effect of the near field topography on the gravity signal while also taking advantage of the 2 m DEM.

For the gravity measurements, we employed the Swisstopo Scintrex CG5 gravimeter, which has a precision of 0.001 mGal. At each location, we measured the gravity signal through 8 cycles of 45 s each. The instrument was then taking a total of 1000 measurements per cycle. We deemed the measurements stable and yielding an acceptable result if the gravity values of the last four cycles were within the uncertainty of ±0.001 mGal, which is the reading resolution of the gravimeter. We then used the average value of the 4 last cycles for further processing. Before and after each day of measurement, we measured the gravity values at either the gravity station of Metas that we used as a base (2′601′930.5, 1′197′018.2, Swiss coordinates) or in the basement of the Institute of Geological Sciences (2′599′200, 1′200′060, Swiss coordinates) to correct the instrumental drifts. A drift rate per hour was calculated linearly (Scintrex, 2012; see discussion in Yu et al. (2015), and Meurers, 2018), which was used to correct the daily measurements. Outliers caused by earthquakes, motorized and pedestrian traffics, bikes, and the gravimeter’s instability after transport were identified in the field. The related information was taken from the live gravity readings and the difference between the average values of each cycle (including jumps of a few mGal between them and trends in the collected gravity data). The resulting average drift rate was c. 0.011 mGal/h with a maximum and a minimum drift rate of c. 0.015 and 0.005 mGal/h, respectively.

### Calculation of the Bouguer gravity values

**Fig. 4:**
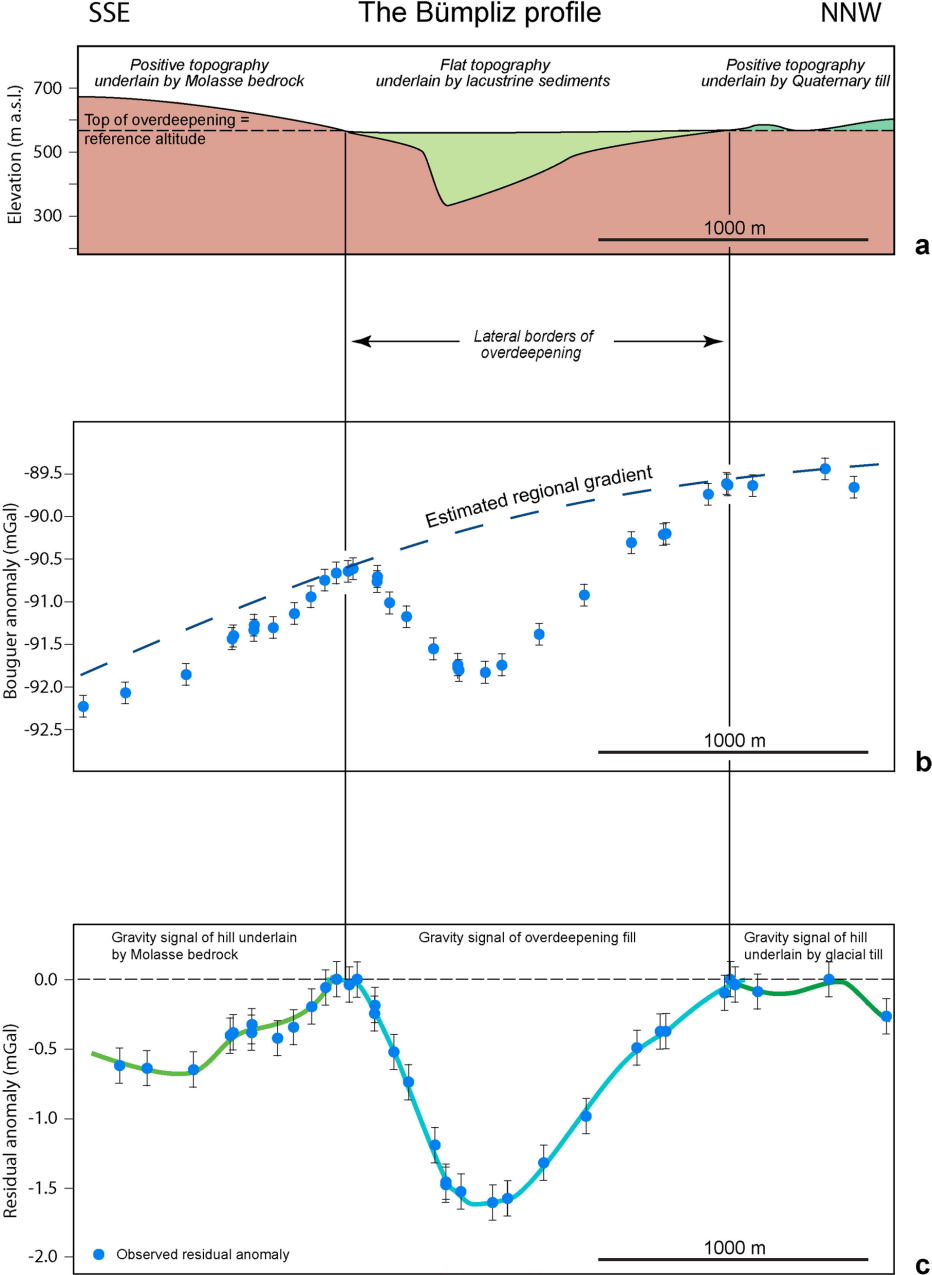
Example of how the residual anomalies were determined, using the Bümpliz profile as an example. **a** The target overdeepening with a Quaternary fill occurs beneath a generally flat surface. It is flanked by a surface topography on either side, which is underlain by Molasse bedrock on the SSE margin, and by a glacial till on the NNW side. **b** We determined the regional gravity gradient using the measured gravity values (blue dots, where the size of a dot corresponds to an estimated average error of ±0.04 mGal while the maximum uncertainty of ±0.13 mGal is shown by error bars, see text) on the lateral boundaries of our target overdeepening. **c** The residual gravity anomalies are determined through calculating the difference between the regional gravity gradient and the measured Bouguer anomalies. Because the Bouguer anomalies were calculated with a standard density of 2670 kg/m^3^, a positive surface topography made up of Molasse bedrock with a density of 2500 kg/m^3^ will yield negative residual anomaly values. Such a signal would disappear if a correction with a density difference of − 170 kg/m^3^ would be applied (difference between the standard Bouguer density of 2670 kg/m^3^ and that of the Molasse bedrock according to Bandou et al., 2022). On the NNW boundary, the positive topography underlain by Quaternary sediments with a density of 2000 kg/m^3^ also yields a negative residual anomaly signal, which could be removed if a correction with a density of − 670 kg/m^3^ would be applied (difference between the standard Bouguer density of 2670 kg/m^3^ and that of Quaternary sediments). If the overdeepening fill would be replaced by Molasse bedrock, then the residual gravity anomaly signals would also disappear. Accordingly the mass of the Quaternary fill causing the negative gravity anomaly signals can be estimated through modelling thereby using information about the density difference between the Molasse bedrock and the Quaternary fill

We used the swisstopo software Quawirk (available from swisstopo upon request and described in Bandou, 2023a) and a standard density of 2670 kg/m^3^ (LaFehr, 1991; Bernabini et al., 1994; Holom and Oldow, 2007) to correct the measured gravity data for gravity signals related to elevation, tides (Hinze et al., 2005) and for the effect of the topography on the gravity values (Bandou, 2023a). We also considered the gravity signal related to the near-field topography using the high-precision GNSS data and the SwissAlti3D DEM with a resolution of 2 m as a basis (protocol available in Bandou, 2023a). We then obtained the Bouguer anomaly value at each MP (measurement point, Additional file 1: Appendix A) upon subtracting these contributions and the normal gravity (according to international reference gravity formula; Hinze et al., 2005) from the measured gravity data. Bandou et al. (2022) estimated a maximum uncertainty of ±0.13 mGal through repeated gravity and GNSS measurements, which we generally employed in this work (to be on the conservative side). However, our average gravity uncertainty including the contribution of the gravity signal related to the near-field topography is ±0.04 mGal, estimated again through repeated measurements of the same station (Additional file 1: Appendix A and Bandou, 2023a). We always show both values either through the size of the error bars or that of the blue circles (e.g., Fig. 4).

### Projection of data and determination of the residual anomalies

As mentioned above, the measured gravity stations follow as much as possible the course of the profiles though due to logistic conditions some deviations could not be avoided. For interpretation purposes (derivation of residual anomalies and gravity modelling) the Bouguer gravity values of all stations belonging to a profile were projected perpendicularly onto the profile (Additional file 3: Appendix C). We deviated from this strategy for the profile across the Bümpliz channel where due to logistic reasons the gravity profile had to be placed obliquely to the orientation of the overdeepening’s long axis (Sect. 4.1 and Additional file 3: Appendix C and Additional file 4: Appendix D). Note that for the derivation of residual anomalies and subsequent modelling we ignored stations that are several hundreds of meters off the target profiles.

The residual anomaly corresponds to the effect caused by the mass of a local structure, which, in our case, is the fill of an overdeepening or the effect of a mountain ridge where the bedrock density is different from the standard 2670 kg/m^3^ used for the Bouguer anomaly calculations (Fig. 4). Note that while principally the Bouguer gravity effect may show the signal of a local excess or a deficit of mass, in our study region we only expect negative anomalies from the overdeepenings as the densities of their infill is lower than that of the Molasse bedrock (Bandou et al., 2022). In this context, Schwenk et al. (2022a) reported differences in the densities between the overdeepening fill and the Molasse bedrock that range from − 300 to – 400 kg/m^3^, whereas Bandou et al. (2022) used the Nettleton method to determine a density difference of – 500 kg/m^3^. These values are in excellent agreement with the results obtained by Kissling and Schwendener (1990) for the sedimentary fill of an overdeepening in the Ticino valley.

The residual gravity anomaly is obtained by calculating the difference between the gravity gradient of the region of interest and the local Bouguer anomaly values along the profile. Therefore, as a first step, we determined the general regional gravity gradient based on the data of the Gravimetric Atlas of Switzerland. We complemented this data by using the results of our own survey, which has a higher spatial resolution along the profiles (Fig. 3). Figure 4 shows the procedure from a conceptual point of view. In this example, an overdeepening with a lake infill and a flat surface is laterally bordered by two mounds underlain either by Molasse bedrock or by a Quaternary till (Fig. 4a). The gravity values measured at the lateral borders of the overdeepening were then used to constrain the course of the regional gravity gradient (Fig. 4b). The consolidated Molasse sediments (either Lower Freshwater Molasse or Upper Marine Molasse, Isenschmid, 2019) has a bulk rock density of 2500 kg/m^3^ (Bandou et al., 2022) that is smaller than the standard 2670 kg/m^3^ value used for Bouguer corrections. Therefore, a positive topography underlain by Molasse sediments will have a negative residual signal. The same is also valid for a positive topography, which is underlain by Quaternary sediments with density values where the contrast to the standard density is even higher (− 670 kg/m^3^ according to Bandou et al., 2022). Accordingly, a negative residual anomaly can either be related to a signal caused by the overdeepening fill and/or by a hill made up of Molasse bedrock and/or Quaternary sediments (Fig. 4c). Such patterns will be considered upon designing the modelling strategy (see Sect. 3.4).

### Gravity modelling using PRISMA

**Fig. 5:**
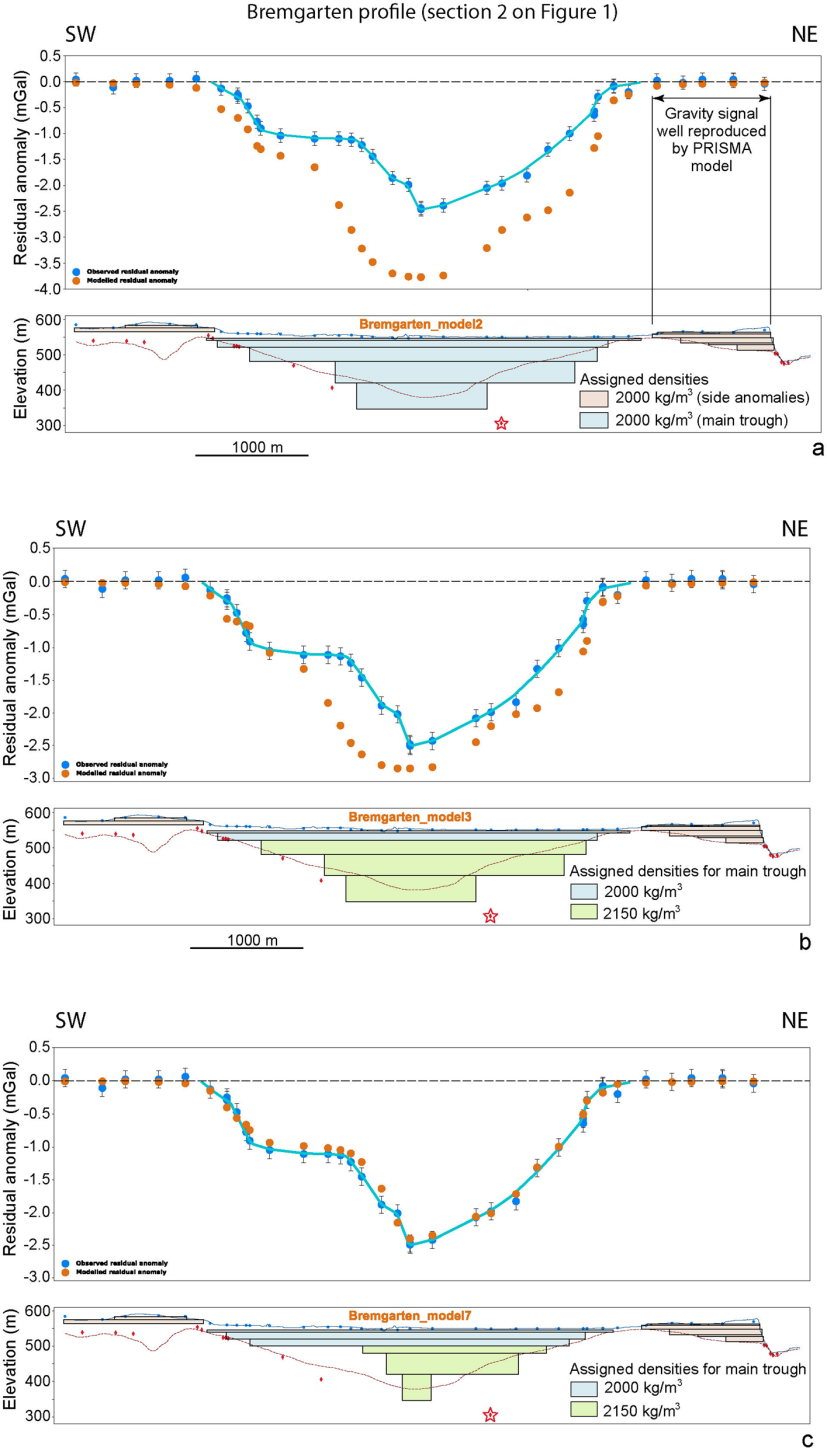
Example (Bremgarten profile, Sect. 2 on Fig. 1), illustrating of how we proceeded upon modelling. **a** The first models were accomplished considering all drilling information and assigning a bulk density of 2000 kg/m^3^ to the Quaternary fill of the main overdeepening and to the positive topography on the lateral margin of the overdeepening, particularly if these topographies are overlain by Quaternary sediments. For the Bremgarten profile, such a first model shows that the residual anomaly on the NE margin is well reproduced by the model, whereas the model largely overestimates the residual anomaly signal of the main overdeepening. **b** The use of a bulk density of 2000 kg/m^3^ for the uppermost part of the Quaternary fill (constrained by the good fit between the modelling results and the measured residual anomalies on the NE margin) and a slightly higher density of 2150 kg/m^3^ for the lower part of the section (constrained by the data from the Rehhag drilling in Schwenk et al., 2022a) improves the fit between the modelling results and the measured residual anomaly values. However, the model still overestimates the gravity signal related to the Quaternary fill of the overdeepenings. Note that we also assigned a density of 2000 kg/m^3^ for the Quaternary masses forming the topographies on either side of the trough. **c** Improvements upon fitting the modelled signal with the measured values were only possible if the widths of the prisms were reduced. We did not further increase the bulk density of the Quaternary fill because this would not be consistent with the density values measured for the Rehhag core by Schwenk et al. (2022a). In addition, such a model would predict a maximum depth for the Molasse bedrock, which would be much lower than the constraints offered by drilling information. The red broken line illustrates the bedrock topography of the model by Reber and Schlunegger (2016). The blue dots are the gravity stations, the red diamonds indicate drillings that reached the bedrock. The black rectangles show the cross sections of the prisms used for modelling. The red star denotes the projected location of the Forsthaus drilling (see Figure D.3.1 for location) Additional file 4:

**Fig. 6:**
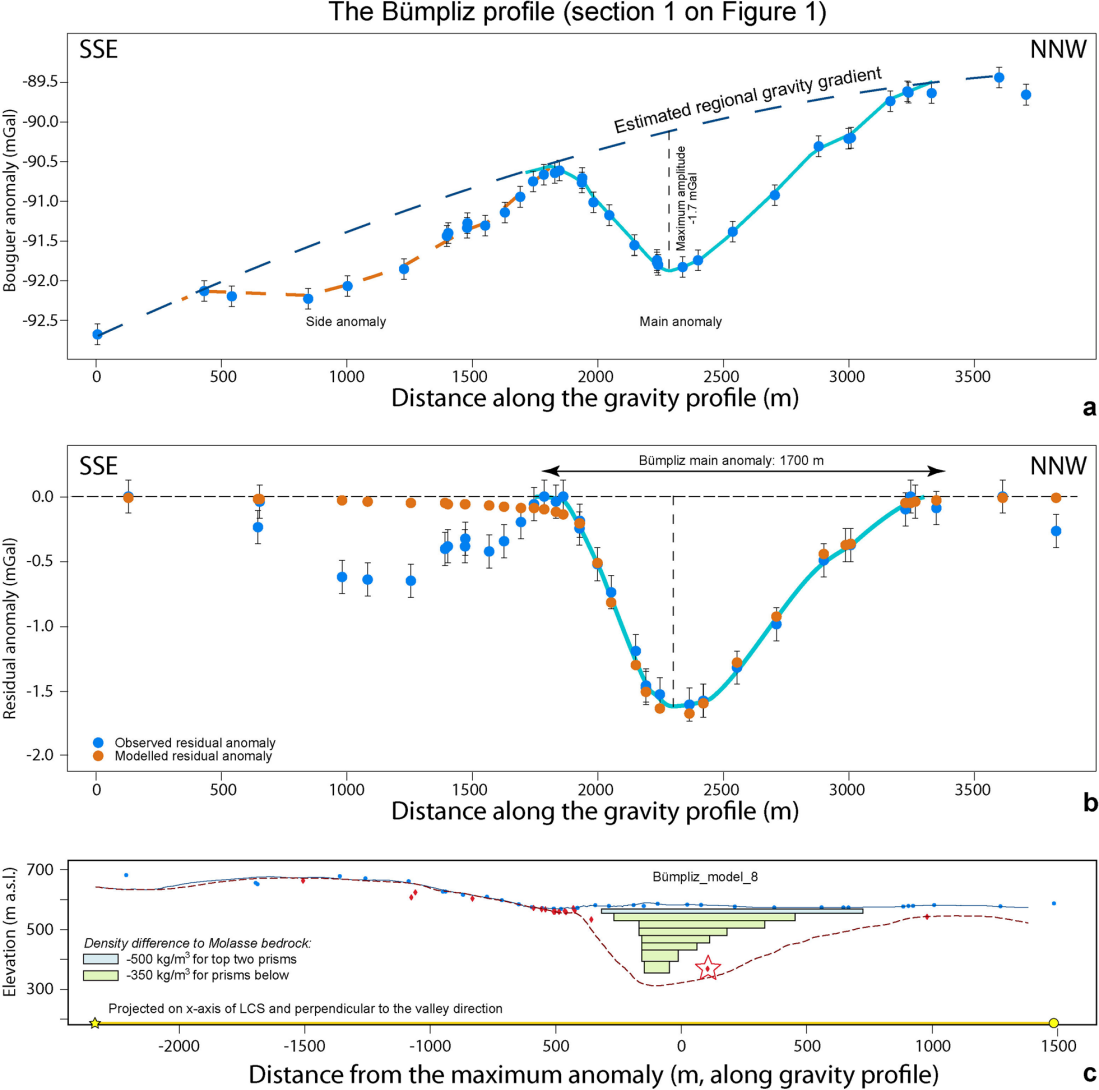
The Bümpliz profile. **a** Bouguer anomalies and regional trend of the gravity field along the profile. The blue dots represent the stations where gravity data was collected for this study. See Additional file 4: Figure D.2.1 for location of stations, Additional file 1: Appendix A for gravity data and Additional file 2: Appendix B for information on the drillings. **b** Results of the final model for the Bümpliz profile, made with a total of 8 prisms with a density contrast of − 500 kg/m^3^ for the top prism and − 350 kg/m.^3^ for the rest of the prisms. The blue dots represent the observed residual anomaly (the dot size corresponds to the average uncertainty of ± 0.04 mGal), and the orange dots are the modelled residual anomaly values for model 8. The black bars indicate our maximum uncertainty of ± 0.13 mGal. The light blue line highlights the main anomaly. **c** Elevation (SwissAlti3D 2 m DEM (© swisstopo)) along the profile (blue solid line). The red broken line illustrates the bedrock topography of the model by Reber and Schlunegger (2016). The blue dots mark the locations of the gravity stations, the red diamonds indicate drillings that reached the bedrock. The black rectangles show the cross sections of the 8 prisms used for modelling. The red star is the projected location of the Rehhag drilling (see Additional file 4: Figure D.2.1 for location)

All of our modelling of the residual anomalies has been accomplished using the PRISMA routine (Bandou, 2023b) that was developed and tested in Bandou et al. (2022). This program allows for the forward modelling of the gravity effects of subsurface objects at freely distributed points (e.g., Nagy, 1966). The routine uses a series of right-angled prisms (they appear as rectangles in the cross-sections, Fig. 5) to approximate the geometry of the structures of interest (Nagy, 1966; Banerjee and DasGupta, 1977; Kissling, 1980; Karcol and Pašteka, 2019), and it calculates the related gravity effects in a right-handed local Cartesian coordinate system (LCS) with the z-axis towards the Earth’s centre. For a successful application of the PRISMA routine, we thus had to define a LCS (see Additional file 3: Appendix C for transformation of coordinates for the case of the Bümpliz profile, which is Sect. 1 on Fig. 1) where the LCS y-axis follows the general direction of the overdeepening and where the x-axis crosses the valley at a right angle. Note that the gravity profiles running more or less perpendicularly to the valley will normally show small angles to the direction of the LCS x-axis (see examples in Additional file 4: Appendix D). We then projected the location of the station with the maximum residual anomaly onto the profile to define the origin of the LCS to facilitate the subsequent forward modelling steps. Note that this site will also represent the origin in the cross sections (point zero) where we present the modelling results (e.g., Fig. 6c). Note also that the calculation of the gravity effect is fully 3D, yet for illustration purposes we projected the results onto the profile.

Prior to modelling, we positioned the prisms parallel to the overdeepenings’ flanks using the contour lines of the Reber and Schlunegger (2016) bedrock model and the cross-sectional shape of the residual anomalies to organize and place the prisms at greater depths. In the same sense, the lengths of the prisms are defined using a priori information offered by the existing bedrock topography model and drillings (Additional file 2: Appendix B). Bandou et al. (2022) documented that prisms with a distance > 1.5 km from the profile will not increase the modelled gravity signal beyond the uncertainty of our field surveys. We therefore used prisms with such a maximum length upon modelling. Note that this is only the case if the prism can be freely extended in the valley (e.g., the Bremgarten profile, see below). In the case where the overdeepening is meandering, as is the case for the Bümpliz channel (Sect. 5.1), we set a limit to the prism where the bedrock turns.

Upon modelling, we started with a series of first prisms using the a priori information offered by the bedrock topography model of Reber and Schlunegger (2016) and the drillings close to the section. We also considered published data on the density contrasts between the Quaternary fill and the Molasse bedrock in the region (Bandou et al., 2022; Schwenk et al., 2022a). The subsequent steps included adding more prisms and adjusting their geometries to better approximate a more complex geometry. For the Bremgarten profile, for instance, the use of a bulk density of 2000 kg/m^3^ for the Quaternary sediments (which corresponds to a density contrast to the Molasse bedrock of – 500 kg/m^3^) and the full consideration of all drilling information allows to reproduce the measured gravity signal along the NE margin of the profile where the topography is underlain by Quaternary sediments. Yet this strategy yields a gravity signal where the wavelengths and amplitudes are too large particularly in the middle of the overdeepening (model 2 on Fig. 5a). The assignment of a larger bulk density of 2170 kg/m^3^ to the lower part of the overdeepening fill (following Schwenk et al., 2022a) in combination with the consideration of all drilling information (model 3 on Fig. 5b) reduces the wavelengths and amplitudes of the resulting gravity values, but the modelled signals are still too large. A further increase in the bulk density of the Quaternary fill would reduce the maximum amplitude of the modelled gravity signal, but the wavelength of the gravity signal particularly for the deepest part would still be too large compared to the observed residual anomalies. Subsequent adjustments where the widths of the prisms were continuously decreased particularly at greater depths, and where the maximum thickness of the overdeepening fill was maintained (as constrained by a drilling, red star on Fig. 5) resulted in an acceptable fit of the modelling results with the observed residual anomalies (Fig. 5c). We already mention here that some drilling information will not fit with the results of the model that best reproduces the measured gravity. This is because drilling provide very local information, while gravity records the effect of the total mass of the Quaternary infill, as documented in Fig. 5. Moreover, due to the complex geometries of the troughs (such as meanders and V-shaped deep incisions), the cross-sectional solutions that we will present below illustrate the general architecture of the troughs on either side of the profiles rather than the local details. All modelling results can be found in Bandou (2023a) and Bandou et al. (2023).

## Results and Interpretation

### The Bümpliz profile (Sect. 1 on Fig. 1)

The gravity data along the Bümpliz profile is characterized by a negative main anomaly with a maximum amplitude of − 1.7 mGal between the profile distances of 1800 m and 3400 m (Figs. 6a, b) where the estimated regional gravity gradient matches with the locally observed gravity (Additional file 4: Appendix D.2). This main anomaly documents an asymmetric V-shaped bedrock depression. The SSE flank of the depression is steeper (> 55°) than its NNW counterpart (< 20°) where the bedrock reaches nearly the surface at a distance of c. 800 m from the deepest point of the depression (Fig. 6c). Near the profile distance 2500 m the depth of the bedrock is a priori known from the results of the Rehhag drillhole (Schwenk et al., 2022a). On the SSE side, the main gravity anomaly is bordered by a side anomaly (Fig. 6b), which is most likely caused by the surface topography and the effect of the Lower Freshwater Molasse bedrock forming a positive topography at the SSE margin of the profile (e.g., Fig. 4). In addition, possible effects related to the Quaternary fill of a bedrock channel aside of the targeted cross section (see Reber and Schlunegger, 2016) could also contribute to this secondary anomaly.

For the first model, we employed a set-up that corresponds to a U-shape geometry thereby incorporating the geometry from the existing bedrock model (Reber and Schlunegger, 2016, red broken line in Fig. 6c), and we considered an asymmetry that is characterized by a much steeper SSE flank compared to the NNW side. Schwenk et al. (2022a) inferred density contrasts ranging from c. − 270 kg/m^3^ to c. − 420 kg/m^3^ between the overdeepening fill and the Lower Freshwater Molasse bedrock based on the results of measurements with a Multi Sensor Core Logger (MSCL; Geotek Ltd.). Accordingly, we started with a model where we used a value of − 300 kg/m^3^ as a first estimate for characterizing the density contrast between the Quaternary deposits and the Lower Freshwater Molasse bedrock. These initial results show that the wavelength of the modelled main anomaly is too wide and extends too far towards the flanks compared to what the gravity the data implies (Additional file 4: Appendix D.2). Subsequent improvements included a shift towards a V-shaped cross-sectional geometry and density contrasts of − 500 kg/m^3^ and − 350 kg/m^3^ (average density value taken from Schwenk et al., 2022a) between the Molasse bedrock and the uppermost few meters of the overdeepening fill, and for the Quaternary suite at deeper levels, respectively. Note that according to Schenk et al. (2022a), the material with a bulk density of 2150 kg/m^3^ (material with a density contrast of − 350 kg/m^3^ to the Molasse bedrock) has a depositional age of MIS 8 and thus experienced the compaction due to several 100 m-thick ice bodies during at least 2 major glaciations.

We iteratively adjusted the model geometry on the lateral flanks (Additional file 4: Appendix D.2) until a best-fit between the modelled and measured residual anomalies was reached. The final model (Fig. 6c) is characterized by a V-shaped cross-sectional geometry with a > 60° steep flank on the SSE margin and a gently dipping flank (< 15°) on the opposite side. In addition, the overdeepening reaches a depth level of c. 350 m a.s.l. See Sect. 5.1 for more information.

### The Bremgarten profile (Sect. 2 on Fig. 1)

**Fig. 7:**
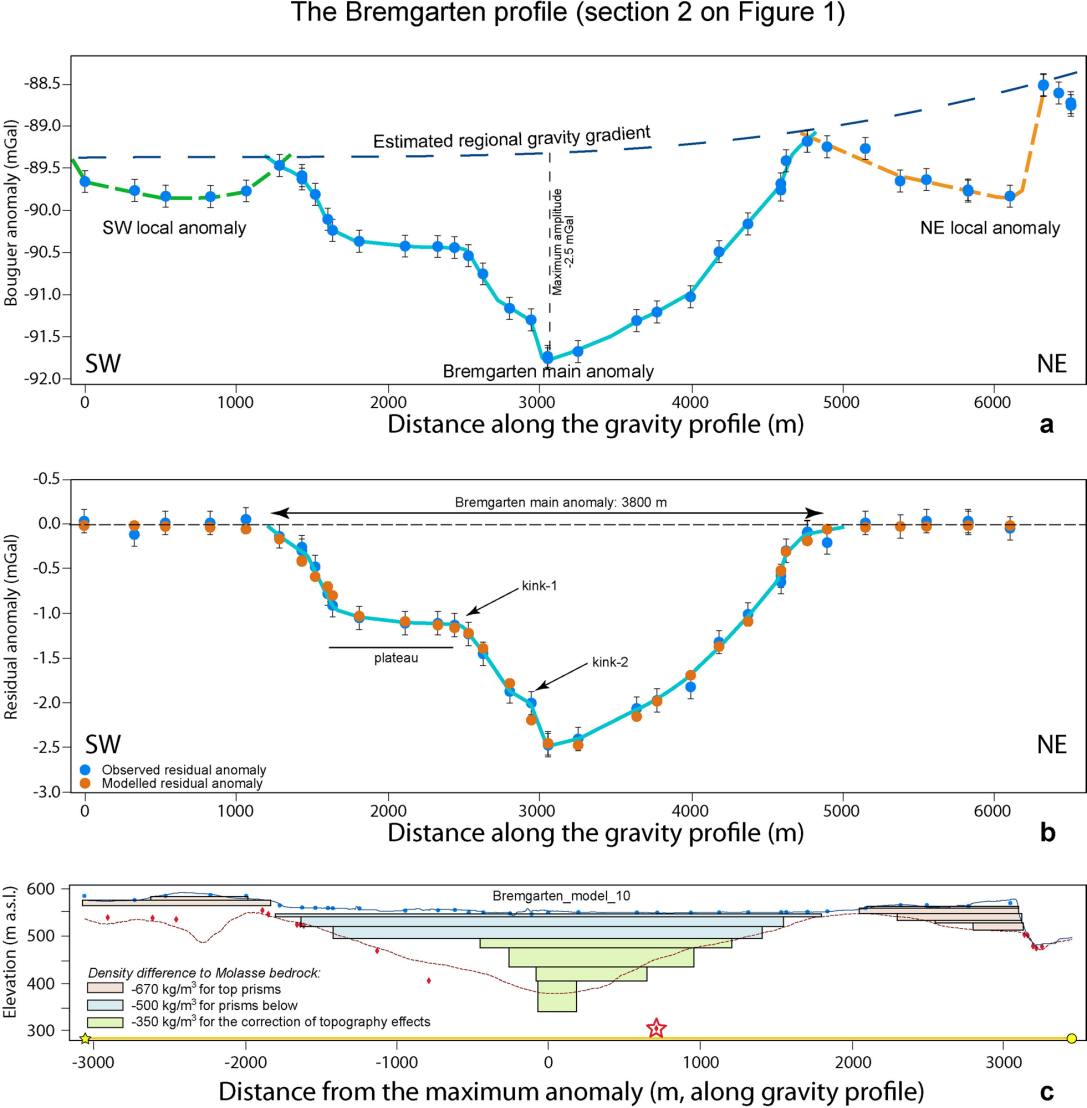
The Bremgarten profile. **a** Bouguer anomalies and regional trend of the gravity field along the profile. The blue dots represent the stations where gravity data was collected for this study. The blue line highlights the main anomaly, while the green and orange broken lines indicate the side anomalies. See Additional file 4: Figure D.3.1 for location of stations, Additional file 1: Appendix A for gravity data and Additional file 2: Appendix B for information on the drillings. **b** Final model for the Bremgarten profile, made with a total of 7 prisms with a density contrast of − 500 kg/m^3^ for the uppermost three prisms and − 350 kg/m^3^ for the rest of the prisms (see also Fig. 5). The blue dots represent the observed residual anomaly (the dot size corresponds to the average uncertainty of ± 0.04 mGal), and the orange dots are the modelled residual anomaly values for model 10. The black bars indicate our maximum uncertainty of ± 0.13 mGal. The light blue line highlights the main anomaly. The effect of the side anomalies was modelled, and the results were subtracted from the residual anomalies. **c** Elevation (SwissAlti3D 2 m DEM (© swisstopo)) along the profile (blue solid line). The red broken line illustrates the bedrock topography of the model by Reber and Schlunegger (2016). The blue dots mark the locations of the gravity stations, the red diamonds indicate drillings that reached the bedrock. The black rectangles show the cross sections of the 7 prisms used for modelling. The red star is the projected location of the Forsthaus drilling (see Additional file 4: Figure D.3.1 for location), though it is quite far off the profile (c. 500 m, see Additional file 4: Figure D.3.1). Figure 7c also shows the intersections of the prisms with the profile that were used to correct the gravity residual anomaly for the side topography effects. This was done using a density contrast of − 650 kg/m^3^ (see Fig. 4 for explanation)

For the Bremgarten profile, we observe a main wavelength anomaly with a maximum amplitude of c. −2.5 mGal that is most likely caused by the sedimentary fill of the tunnel valley, and two short-wavelength and low-amplitude local anomalies on either side of the main residual gravity anomaly (Fig. 7). In the main trough, the residual anomalies point towards a strong asymmetry between the NE and SW valley flanks, with the latter also having a more complex geometry. The base of the overdeepening seems to be narrow and V-shaped, steeper on the SW side than on the NE. Further up, the through widens with both flanks now seemingly having a similar slope, before the occurrence of a plateau on the SW side creating a much wider upper part. On the other side, the NE flank seems to keep a more uniform slope until the near surface. Figure 7 thus clearly defines the tunnel valley residual anomaly wavelength along the profile of approximately 3.8 km.

Upon modelling the gravity signal of the main anomaly (Fig. 5), we found that the residual anomalies along the Bremgarten profile can best be explained by the sedimentary fill where the upper part has a lower density than the basal part. This confirms the asymmetry of the two flanks: The NE flank is wide and U-shaped, while the SW flank has multiple steps and apparently a large plateau in the shallower part of the trough (Figs. 5c, 7b). The geometry documents at least three main storeys, a wide U-shaped upper section and a narrower and steeper middle storey especially on the SW flank. In addition, the lowermost part is narrow and V-shaped (Fig. 7c). All storeys appear to be separated by a kink in the pattern of the residual gravity anomalies. Note that because of the complex geometry of the overdeepening network (convergence between main and the Bümpliz channel), and since the main channel might be shallowing-up and narrowing towards the NW (Fig. 2, Reber and Schlunegger, 2016), the drillings on the SW flank are not fully representative of the general architecture of the overdeepening in the vicinity of the profile that is captured by gravity information.

### The Bern1 profile (Sect. 3 on Fig. 1)

**Fig. 8:**
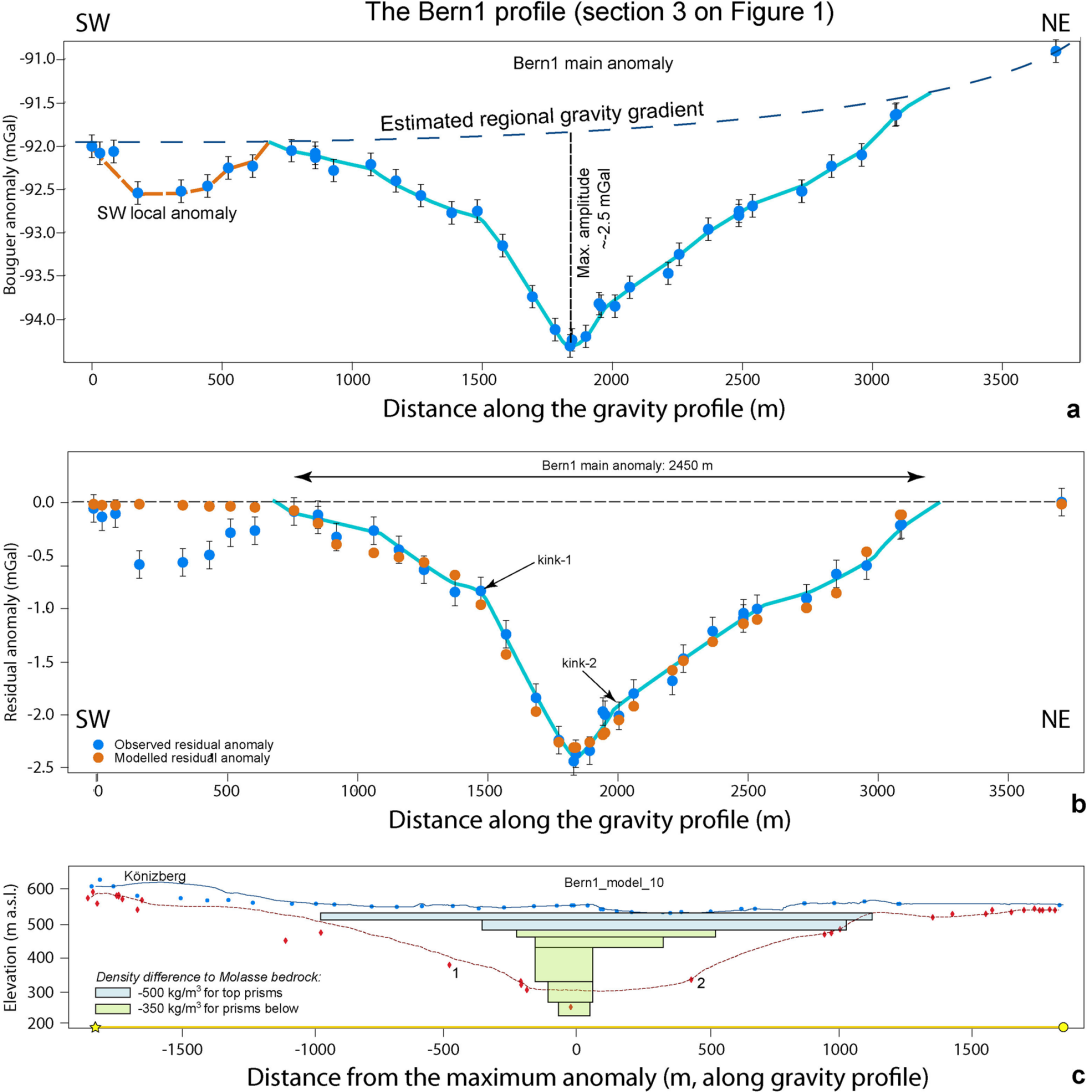
The Bern1 profile. **a** Bouguer anomalies and regional trend of the gravity field along the profile. The blue dots represent the stations where gravity data was collected for this study. The blue line highlights the main anomaly, while the orange broken line indicates the side anomaly on the SW. See Additional file 4: Figure D.4.1 for location of stations, Additional file 1: Appendix A for gravity data and B for information on the drillings. **b** Final model for the Bern1 profile, made with a total of 7 prisms with a density contrast of − 500 kg/m^3^ for the top two prisms and − 350 kg/m^3^ for the rest of the prisms. The blue dots represent the observed residual anomaly (the dot size corresponds to the average uncertainty of ± 0.04 mGal), and the orange dots are the modelled residual anomaly values for model 10. The black bars indicate our maximum uncertainty of ± 0.13 mGal. The light blue line highlights the main anomaly. **c** Elevation (SwissAlti3D 2 m DEM (© swisstopo)) along the profile (blue solid line). The red broken line illustrates the bedrock topography of the model by Reber and Schlunegger (2016). The blue dots mark the locations of the gravity stations, the red diamonds indicate drillings that reached the bedrock. The drillings labelled with 1 and 2 are discussed in Sect. 5.1. The black rectangles show the cross sections of the 7 prisms used for modelling

The gravity data along the Bern1 profile is characterized by a negative main anomaly between the profile distances of 650 m and 3100 m (Fig. 8) where the estimated regional gravity gradient matches with the locally observed gravity (Additional file 4: Appendix D.4). It has a maximum amplitude of − 2.5 mGal and a wavelength of c. 2450 m This main anomaly documents an asymmetric bedrock depression where the upper part has a U-shaped cross-sectional geometry characterized by a relatively wide and shallow depression with a residual anomaly of c. − 0.9 mGal, while the lower part is narrow and deep and displays a residual anomaly contriubution of c. − 1.6 mGal. The U-shaped cross-sectional geometry for the upper part mainly becomes visible when considering the residual anomaly pattern of the SW flank, where the values display a distinct break-in-slope at c. 1500 m profile distance (kink-1), after which the anomaly shows a trend towards increasingly negative values farther to the NE. Obviously, for this deeper part, the SW flank of the depression is steeper than its NE counterpart where the data implies the occurrence of a bedrock flank that continuously dips at a shallow angle towards the site with the largest residual anomaly amplitude, which is situated at c. 1800 m profile distance. It is only at profile distance 2000 m that the residual anomalies of the NE flank steeply dip towards the centre of the bedrock depression (Fig. 8b), marking a second break-in-slope (kink-2 on Fig. 8b). On the SW side, the main gravity anomaly is bordered by a side anomaly (Fig. 8). It has a wavelength of about 700 m and an amplitude of − 0.5 mGal. Apparently, this local anomaly is caused by the effect related to the density of the Molasse bedrock underlying the Könizberg (see Fig. 4 for explanation). In addition, the Quaternary fill of the side channel farther south could also contribute to this negative residual anomaly signal (Additional file 4: Figure D.1). This is the case for the stations located on the SE side of the Könizberg mountain ridge (Fig. 8 and Additional file 4: Figure D.1).

Upon modelling the gravity response of the Quaternary fill, we first explored the effects of different density contrasts between the bedrock and the sedimentary infill of the tunnel valley. The results show that although a higher density contrast of − 500 kg/m^3^ yields a maximum gravity response that is nearly twice the maximum amplitude of the observed residual anomaly (Additional file 4: Appendix D.4), the anomaly of the NE upper flank is perfectly reproduced by the model. This indicates that this density contrast might be applicable for the top part of the overdeepening, while a lower density contrast of -350 kg/m3 is likely appropriate for the lower part of the overdeepening fill (Additional file 4: Appendix D.4). The further modelling steps finally ended with a cross-sectional geometry that has a U-shaped top part where the material is likely to have a density contrast of − 500 kg/m^3^, and a deeper V-shaped segment filled with Quaternary material that has a density contrast of − 350 kg/m^3^ (in comparison to the density of the Molasse bedrock). The upper part reaches a depth level of c. 480 m a.s.l., while the base of lower part is situated at > 250 m a.s.l. and is bordered by flanks that are nearly vertical (Fig. 8c).

We note, however, that at some stations the residual anomaly values cannot fully be reproduced with the model (e.g., at 1100 m and 1550 m profile distance), which we explain by the fact that side channels and meanders introduce a complexity that cannot be considered by prisms alone. Furthermore, we also note that the depths of some drillings are far off the modelled solution, particularly for the deeper V-shaped part of the overdeepening. As outlined below, we explain this misfit by the erosional mechanism, which was most likely accomplished with water (due to the V-shaped character). Bedrock incision by water results in the formation of meanders, deep and narrow gorges, and sharp turns at short downstream distances, which cannot be reproduced by prisms simply following the longitudinal trend of the main channel’s depression. Therefore, while our gravity models perfectly reproduce the overall character of the overdeepening’s geometry for the cross section (i.e. wide and U-shaped top and a narrow V-shaped, and deep base), it fails to reproduce meanders, side channels and local features that occur over a short down-stream distance. While such features can accidently be hit by a drilling, in most cases their gravity effects are of too low an amplitude and too short a wavelength to be assessed by gravity data particularly if they occur at > 100 m depths beneath the surface (see Sect. 5.1 in discussion).

### The Bern2 profile (Sect. 4 on Fig. 1)

**Fig. 9:**
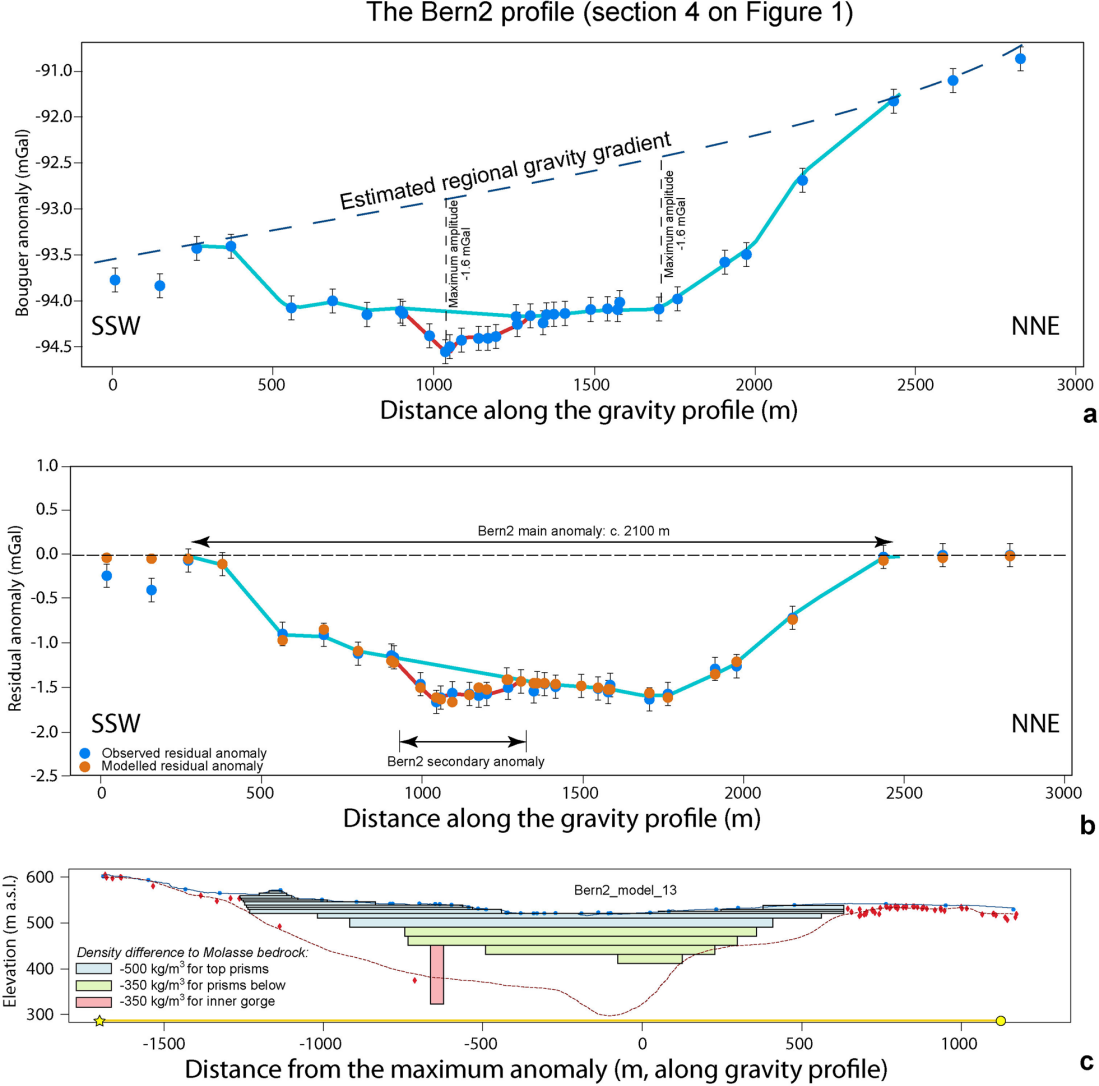
The Bern2 profile. **a** Bouguer anomalies and regional trend of the gravity field along the profile. The blue dots represent the stations where gravity data was collected for this study. The blue line hightlights the main anomaly, and the red line indicates the gravity effect of the inner gorge. See Additional file 4: Figure D.5.1 for location of stations, Additional file 1: Appendix A for gravity data and B for information on the drillings. **b** Final model for the Bern2 profile (including the topographic signal), made with multiple prisms with a density contrast of − 500 kg/m^3^ for the top prisms and − 350 kg/m^3^ for the rest of the prisms. The blue dots represent the observed residual anomaly (the dot size corresponds to the average uncertainty of ± 0.04 mGal), and the orange dots are the modelled residual anomaly values for model 13. The black bars indicate our maximum uncertainty of ± 0.13 mGal. The light blue line highlights the main anomaly, the red line shows the secondary anomaly of the inner gorge. **c** Elevation (SwissAlti3D 2 m DEM ( © swisstopo)) along the profile (blue solid line). The red broken line illustrates the bedrock topography of the model by Reber and Schlunegger (2016). The blue dots mark the locations of the gravity stations, the red diamonds indicate drillings that reached the bedrock. The black rectangles show the cross sections of the prisms used for modelling. Please refer to Additional file 4: Appendix D.5 for more information

For the Bern2 profile, we determine a local residual gravity anomaly that has a wavelength of about 2100 m and is approximately U-shaped. We additionally identify two locations separated by about 700 m that show a maximum amplitude of − 1.6 mGal (Additional file 4: Appendix D.5 and Fig. 9a). In addition, the SSW flank of the anomaly is less uniform (i.e., there are kinks and a plateau) than the NNE flank, where Bouguer gravity values change in steps.

On a closer inspection of the gravity field, we conclude that there probably exist two gravity anomalies of significantly different wavelengths that overlap (Fig. 9a). The longer wavelength anomaly (blue solid line) suggests the occurrence of a wide somewhat asymmetric trough reaching a maximum depth at around 1700 m profile distance. The shape of this long-wavelength anomaly on the NNE flank well corresponds to the geometry proposed by Reber and Schlunegger (2016). The short wavelength (width of approximately 350 m) local anomaly (solid red line) reaching an additional − 0.5 mGal (relative to the longer wavelength anomaly, see Fig. 9a) near profile distance 1000 m suggests that there exists a significant (deep and narrow) local bedrock depression underneath the otherwise rather smoothly and gently dipping SSW flank of the main trough.

Upon modelling (Additional file 4: Appendix D.5), we first considered the gravity signal exerted by the positive topography on either side of the valley (Bandou, 2023a), where the surface is underlain by Quaternary sediments as disclosed by drillings (Reber et al., 2016). After subtraction of this topography signal from the residual anomalies (Bandou, 2023a), we received the anomaly values that most likely resulted from the sedimentary fill of the overdeepening. For this correction, we used a density contrast of − 500 kg/m^3^, because the sediments are part of the overdeepening fill, therefore contributing to the main anomaly effect (see Fig. 4 for explanation of the modelling concept). Note that in Fig. 9, we illustrate the combined effect of the overdeepening fill and the contribution of the surface topography. Yet these results are also shown in the Additional file 4: Appendix D.5 where the topographic signal was removed. Upon modelling, we found that the main wavelength and amplitude of the remaining signal can be explained by the fill of an overdeepening that has a general U-shaped cross-sectional geometry with a deepest location situated at 1700 m distance from the start of the profile (Fig. 9b) or at 0 m if the LCS is used as reference (Fig. 9c). The general shape of the overdeepening corresponds well with the model by Reber and Schlunegger (2016) yet the maximum depth reached by this U-shaped depression is just half of it (Fig. 9c). In addition, the fill of this main trough reveals two storeys where the material of the upper part has a lower density than the sediments at the bottom of the overdeepening. However, the local gravity anomaly of about −0.3 mGal (after removal of the topography signal, Additional file 4: Figure D.5.5) and about 350 m wavelength remains unexplained by the sedimentary infill of the main trough. This signal could likely be caused by a sedimentary fill of an inner gorge structure in the bedrock. In order to model such a feature, we introduced a new prism, which we placed along the axis of the main channel. This prism has a length of c. 200 m in both directions from the profile (red prism on Fig. 9c). We first selected a width of 40 m, a value which is based on the largest width of the Aare inner gorge between Innertkirchen and Meiringen (measured on the LiDAR-based DEM of Swisstopo and described in Hantke and Scheidegger (1993)), and we used a thickness of 20 m (equivalent to the deepest prism of the main trough) as a first very conservative estimate. We then adjusted the width and thickness of the prism until a best-fit was reached between the model results and observations (Bandou, 2023a). This finally yielded model 13 (Figs. 9b, c), which would be a reasonable estimate for the geometry of the inner gorge. However, drilling data (e.g., at the Marzili c. 600 m farther to the SE, see Fig. 1) shows that the inner gorge could possibly be deeper. Indeed, further modelling considering a narrower but deeper inner gorge yields the same results. This shows the ambiguity of the gravity method and that we reached the limit offered by the selected approach (i.e., using prisms to approximate 3D structures).

### The Kehrsatz profile (Sect. 9 on Fig. 1)

**Fig. 10:**
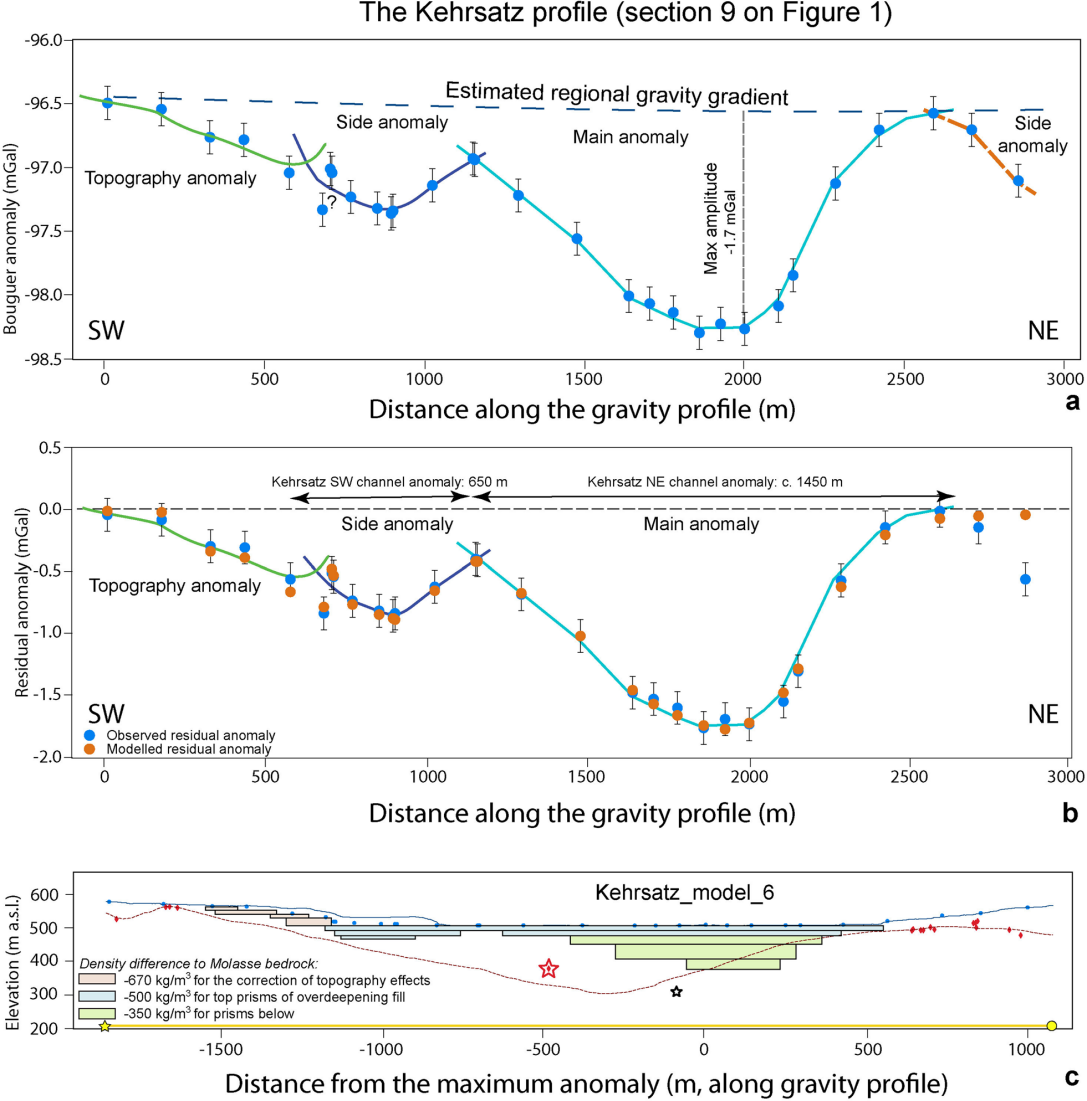
The Kehrsatz profile. **a** Bouguer anomalies and regional trend of the gravity field along the profile. The blue dots represent the stations where gravity data was collected for this study. See Additional file 4: Figure D.6.1 for location of stations, Additional file 1: Appendix A for gravity data and B for information on the drillings. Three MPs at c. 300 m profile distance show a zig-zag-pattern in the gravity signals (denoted with a question mark), which might be caused by local effects that we cannot fully explain. The green line indicates the topography effect, the dark blue line the gravity signal of the side anomaly, the pale blue line denotes the signal of the main trough, and the orange dashed line on the NE margin denotes the effect of a further side anomaly. **b** Final model for the Kehrsatz profile (including the topographic signal), made with seven prisms with a density contrast of -500 kg/m^3^ for the top four prisms and − 350 kg/m^3^ for the three remaining prisms. The blue dots represent the observed residual anomaly (the dot size corresponds to the average uncertainty of ± 0.04 mGal), and the orange dots are the modelled residual anomaly values for model 6. The black bars indicate our maximum uncertainty of ± 0.13 mGal. **c** Elevation (SwissAlti3D 2 m DEM (© swisstopo)) along the profile (blue solid line). The red broken line illustrates the bedrock topography of the model by Reber and Schlunegger (2016). The blue dots mark the locations of the gravity stations, the red diamonds indicate drillings that reached the bedrock. The red and white stars (both are > 900 m to the SE of the profile) indicate locations of two deep drillings, projected onto the profile. The red one reached the bedrock, the white one ended in the Quaternary sediments (see Figure D.6.1 for locations). The black rectangles show the cross sections of the prisms used for modelling. Please refer to Additional file 4: Appendix D.6 for more information

The 30 gravity stations of the Kehrsatz gravity profile provided us with a residual anomaly consisting of two parts (Additional file 4: Appendix D.6 and Fig. 10a): A shorter wavelength and lower amplitude anomaly of about − 0.8 mGal on the SW side between c. 500 m and c. 1150 m profile distance (side anomaly with dark blue colour on Fig. 10a), and a main anomaly between c. 1150 m to c. 2600 m profile distance reaching a maximum amplitude of − 1.7 mGal (pale blue line on Fig. 10a). This main anomaly shows a nearly U-shaped geometry with a pronounced asymmetry as the NE flank appears to be steeper.

Upon modelling (Additional file 4: Appendix D.6), we first considered the gravity effect exerted by Quaternary sediments underlying the topography on the SW margin of our profile (topography anomaly indicated with the green line on Fig. 10a, and Figs. 4 and 5 for methodological approach). We then iteratively modified the cross-sectional widths of our model prisms and adjusted the density contrast between the Quaternary fill and the Molasse bedrock until we found a best fit between the model results and our observations (Additional file 4: Appendix D.6, and Bandou, 2023a). We ended with model 6 (Figs. 10b, c), which consists of two depressions separated by a local bedrock ridge c. 15 m beneath the surface. The side depression on the SW side is shallow with a depth of 40 m, and it shows a U-shaped cross-sectional geometry. The cross-sectional geometry of the main depression is also U-shaped with a wide and flat bottom. It has an asymmetry with a flank that is steeper on the NE than on the SW side. Please note that similar to Bern2, the model results displayed in Fig. 10 include both the topography signal and that of the main and side channels (see also Additional file 4: Figure D.6.4 for the results where the topographic signal was removed). This final model 6 (and also the residual anomaly pattern) shows significant differences to the bedrock model of Reber and Schlunegger (2016), where the lack of deep borehole data at this locality leads these authors to propose a much wider and deeper overdeepening, with a maximum depth located farther to the SW. Note that similar to the other profiles farther downstream, the bottom part of the overdeepening hosts material that has most likely a lower density contrast than the upper part. Moreover, the thickness of the upper part with a higher density contrast (c. 30 m) is nearly the same as in Bern2 (c. 30 m as well). Note also that upon modelling, we could not exclude that the bedrock depressions at Kehrsatz are underlain and thus cut by a deep inner gorge.

### The Airport profile (Sect. 10 on Fig. 1)

**Fig. 11:**
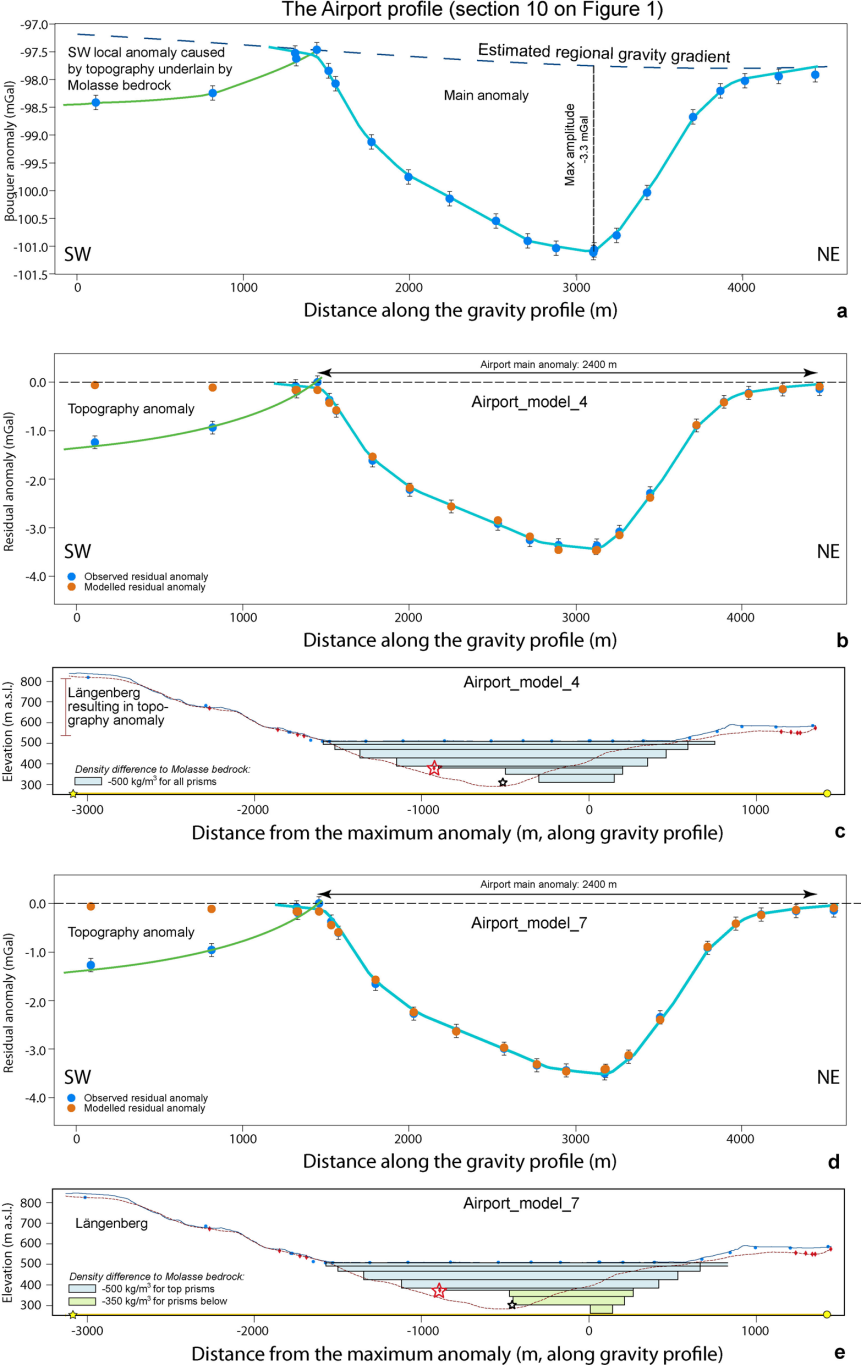
The Airport profile. **a** Bouguer anomalies and regional trend of the gravity field along the profile. The blue dots represent the stations where gravity data was collected for this study. The blue line indicates the main anomaly; the orange broken line refers to the side effect on the SW. See Additional file 4: Figure D.7.1 for location of stations, Additional file 1: Appendix A for gravity data and B for information on the drillings. **b** and **c** Model 4 for the Airport profile, made with seven prisms with a uniform density contrast of − 500 kg/m^3^. **d** and **e** Model 7 for the Airport profile, made with eight prisms with a density contrast of − 500 kg/m^3^ for the top five prisms and − 350 kg/m^3^ for the three bottom prisms. For all figures: The blue dots represent the observed residual anomaly (the dot size corresponds to the average uncertainty of ± 0.04 mGal), and the orange dots are the modelled residual anomaly values. The black bars indicate our maximum uncertainty of ± 0.13 mGal. The light blue line highlights the main anomaly. The figures also show the elevation (SwissAlti3D 2 m DEM (© swisstopo)) along the profile (blue solid line). The red broken line illustrates the bedrock topography of the model by Reber and Schlunegger (2016). The blue dots mark the locations of the gravity stations, the red diamonds indicate drillings that reached the bedrock. The red and white stars indicate locations of two deep drillings, projected onto the profile. The red one reached the bedrock, the white one ended in the Quaternary sediments (see Additional file 4: Figure D.7.1 for locations). The black rectangles show the cross sections of the prisms used for modelling. Please refer to Additional file 4: Appendix D.7 for more information

The Bouguer anomaly pattern of the Airport profile shows a wide U-shaped geometry, which is additionally asymmetric with a steeper NE flank. Two local gravity anomalies are identifiable along the Airport profile (Fig. 11). The first one, situated on the SW side, has an amplitude of c. − 1.2 mGal and is caused by the Molasse bedrock of the Längenberg (topography anomaly), which is a mountain ridge to the West (see also Fig. 4). A second anomaly with a maximum amplitude of − 3.3 mGal, which we consider as the main anomaly, documents the effect of the sedimentary infill of the valley’s overdeepening. This large local anomaly extends from c. 1500 m to c. 3900 m distance along the profile, and the maximum amplitude is measured at the MP 6008 situated at c. 3100 m distance (Additional file 4: Figure D.7.3 and Fig. 11a). This main anomaly has a relatively steep NE flank and a flatter but almost concave SW flank. The gravity data additionally suggest that the deepest part of the overdeepening is situated farther to the NE than what is proposed by the bedrock model of Reber and Schlunegger (2016). Moreover, contrarily to the bedrock topography model of Reber and Schlunegger (2016), the Bouguer anomaly shows that the flank of the overdeepened valley dips much more steeply on the NE side, and that the flank is not uniformly dipping towards the centre on the SW side.

The PRISMA modelling was conducted to reproduce an overall U-shaped geometry of the main anomaly with bedrock flanks that differ in their dip angles. As an initial model, consisting of 4 prisms with a cumulative thickness of 200 m as given by the drilling information, we selected a setup with a constant density contrast of − 500 kg/m^3^ between the Molasse bedrock and the overdeepening fill following Bandou et al. (2022). The results disclosed that the bedrock depth as encountered by the drillings could be reproduced upon modelling (Figs. 11d, e), but the models failed to reproduce the width of the trough at greater depths as inferred by the Rb9202 (white star) drilling (Fig. 11c). Subsequent modelling revealed that a slightly better fit between modelled and observed gravity is possible by using a density contrast of − 350 kg/m^3^ for the lower three prisms (Additional file 4: Figure D.7.7b; Fig. 11e). Apparently, this alternative solution for the geometry of the lower part of the Airport profile also correlates with the width as constrained by the Rb9202 drilling (Additional file 4: Appendix D.7).

We finally found two solutions (model 4 and model 7, Fig. 11c and e, respectively), where the calculated residual anomalies adequately fit the observed ones. Both model solutions show the same geometry for the upper section of the overdeepening pointing towards an asymmetrical U-shaped geometry for the bedrock topography underneath the overdeepening fill. This upper part reaches a depth level of c. 400 m a.s.l. However, the two modelled geometries differ regarding the lower sections. The first solution with one single density contrast of − 500 kg/m^3^ infers the occurrence of a flat and wide U-shaped deeper part with a maximum depth level of c. 320 m a.sl.. In contrast, the second model solution with a density contrast of − 350 kg/m^3^ for the lower part returns a geometry where the bedrock shape is narrow and V-shaped in the deepest part, reaching a depth level of c. 280 m a.s.l. Here, we preferred the second model because it is more consistent with that of the nearby Kehrsatz profile.

### The Bern3, Bern4, Wabern1 and Wabern2 profiles (Sect. 5, 6, 7 and 8 on Fig. 1)

**Fig. 12:**
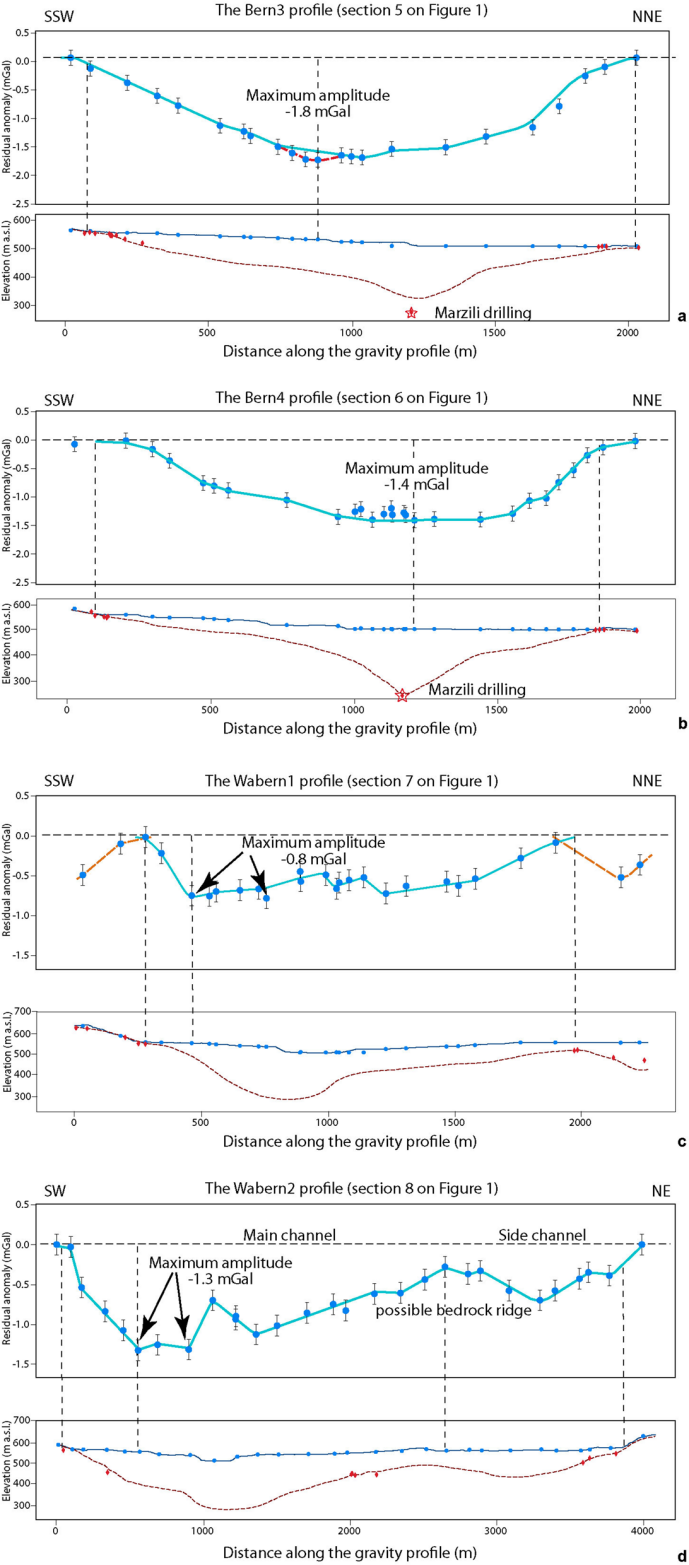
Residual anomalies of the **a** the Bern3, **b** the Bern4, **c** the Wabern1 and **d** the Wabern2 profiles. On the top plots, the blue dots represent the station locations where gravity data was collected for this study. For all profiles, the blue line highlights the main anomaly, whereas for Bern3, the red line indicates the inner gorge effect. For Wabern1, the broken orange lines on both sides denote the side anomalies. For the bottom plots, the elevation (SwissAlti3D 2 m DEM (© swisstopo)) along the profile is represented by the blue solid line. The red broken line illustrates the bedrock topography of the model by Reber and Schlunegger (2016). The blue dots mark the locations of the gravity stations, the red diamonds indicate drillings that reached the bedrock. Note that for the Bern3 and Bern4 profiles, the red star indicates the location of the Marzili drilling. Please see Figs. 3 and Figures D.8 for location of profiles and stations, Additional file 1: Appendix A for gravity data and B for information on the drillings

The complexity of the bedrock topography beneath the city of Bern, which is characterized by multiple side channels and inner gorges (Reber and Schlunegger, 2016) prevented us from modelling the residual anomalies of the Bern3, Bern4, Wabern1 and Wabern2 profiles (Fig. 12). We therefore present the residual anomalies of these profiles only (see Additional file 4: Appendix D.8 and Fig. 1 for location of profiles). Along the Bern3 profile (Fig. 12a), the residual anomalies show the occurrence of a typical U-shaped overdeepening. The anomaly wavelength is approximately 2 km, and we estimate a maximum amplitude of c. − 1.8 mGal. In addition, on the SSW flank, the signal of the overdeepening appears to be continuously dipping towards the base, which appears to be flat. The anomaly signal of the NNE flank, however, has a concave shape, suggesting a steeper middle section, which transitions into a flat base. In addition to this main anomaly with an amplitude of − 1.8 mGal, we also identified a possibly secondary short wavelength anomaly on the SW side, which is similar to the Bern2 profile. In comparison to the existing bedrock topography model of Reber and Schlunegger (2016), the residual anomalies suggest the occurrence of a wider trough with a deepest part that is shifted more towards the SSW.

The Bern4 profile (Fig. 12b) discloses a residual anomaly that has a U-shaped cross-sectional geometry and that is asymmetric. Similar to Bern3, the SSW flank of the overdeepening appears to be continuously dipping towards the base albeit in a less uniform way than at Bern3. The base appears to be generally flat, but locally with a possibly complex bedrock topography. The anomaly signal of the NNE flank, however, points towards a steeper side, which gradually tapers towards the base. Apparently, there is a short plateau at c. − 0.8 mGal on both flanks. The main anomaly has a wavelength of 1.7 km and a maximum amplitude of − 1.4 mGal, which is slightly less than at Bern3. Compared to the existing bedrock topography model of Reber and Schlunegger (2016), the residual anomaly suggests a wider and U-shaped trough. Because of this shape, the information offered by the Marzili drilling (bedrock encountered at 266 m depth) points towards the occurrence of a very narrow and possibly deep inner gorge, as suggested by the bedrock model of Reber and Schlunegger (2016). It appears that the inferred gorge is too narrow to be detected on a gravity profile or too complex to be resolved with a profile- and prism-approach.

The residual anomaly of the Wabern1 profile (Fig. 12c) point towards an overdeepening that has roughly a U-shaped cross-sectional geometry and that is asymmetric. The maximum amplitude is only c. − 0.8 mGal and measured at two locations. In comparison with all other profiles this denotes the shallowest trough encountered in this study. We suggest that on the SSW side, the bedrock is steeply dipping towards the base, which itself is generally flat. On the other side, the flank appears flatter, thereby gradually transitioning towards the basal part. The bedrock topography model of Reber and Schlunegger (2016) points towards the occurrence of multiple side channels in this area. Accordingly, we cannot exclude that one or several bedrock ridges occur underneath the Wabern1 profile, which would correspond to the zig-zagging shape of the residual gravity anomaly around 1000 m profile distance.

Finally, the Wabern2 profile (Fig. 12d) shows a residual anomaly pattern that is roughly U-shaped and asymmetric. The maximum amplitude is c. − 1.3 mGal and measured at two locations, similar to Wabern1. We suggest that on the SSW side, the bedrock is steeply dipping towards the base, which itself is generally flat and possibly shallow. On the NE side, the flank appears flatter, and its limit is difficult to define because of the occurrence of a side channel (see also Reber and Schlunegger, 2016). The main channel is c. 2.6 km wide, whereas the side channel is much narrower (1.2 km) and has a maximum anomaly of c. − 0.7 mGal. The inferred bedrock ridge at c. 2700 m distance is several hundred meters wide. In addition, the main channel could actually be split into two channels by a narrow ridge around 1200 m profile distance.

## Discussion

### Modelling framework: density contrasts, spacing of stations and resolution

Our revision of the existing bedrock topography model (Reber and Schlunegger, 2016) crucially depends on the constraints by the gravity values assigned to the Molasse bedrock and the Quaternary fill of overdeepenings, and on the spatial resolution of our gravity survey. We discuss these points in the following section together with the strengths and weaknesses of the modelling framework used in this paper.

#### Strength and limitations of our modelling framework

Our examples show that the strength of the selected strategy (determination of the residual gravity anomalies in combination with the application of the PRISMA model) lies in the precise geometric reconstruction of the overdeepenings’ flanks, particularly where these are steep (> 60°). However, our setup also has some limitations mainly where the overdeepening is not straight and displays meanders. We encountered such conditions for those profiles where the troughs are narrow and V-shaped and where the residual anomaly showed evidence for an inner gorge (see the Bern2 profile in Sect. 4.4, and discussion below). In such cases, the modelling with PRISMA fails to properly consider the constraints offered by drillings. For the Bümpliz profile, as another example, the deepest drilling in the region (Schwenk et al, 2022a, b; see the red star in Fig. 6c) is located in a bend within the trough and c. 200 m away from our gravity profile (Additional file 4: Figure D.2.4). In such cases where we have to combine local constraints offered by drillings with information about the overdeepenings over a larger scale such as gravity data, we preferentially based our interpretation on the gravity data because in a cross-section the drillings are likely not to document features that are representative at the wavelength scale of the overdeepened trough. Indeed, it was not the scope of our modelling exercise to reproduce geometric details such as meanders. We rather aimed at constraining the overall cross-sectional shape of this particular channel (e.g., at Bümpliz) and also that of the Aare main overdeepening, and we designed the field campaign and the modelling strategy accordingly.

#### Comparison between bedrock model based on drillings and our gravity survey

The comparison between the bedrock topography model that is based on drillings and our profiles, which we reconstructed using gravity data, shows substantial differences for nearly all profiles. This mainly concerns the size of the deepest channel, which in many places is much narrower according to our model than what was proposed in Reber and Schlunegger (2016) where the troughs appear much larger and wider. The reason for this difference can be explained by the lack of drillhole data to constrain the details of the overdeepenings’ flanks, as explained by Reber and Schlunegger (2016). In fact, while these authors had sufficient information to reconstruct the shallow parts of the overdeepened troughs at a high level of details, they did not have enough drilling information to determine the dip angle of the overdeepening’s flanks. Therefore, they inferred dip angles that continuously increase and then decrease with depth, with the consequence that the resulting cross-sectional width became larger and more concave towards the bottom than what we suggest based on gravity data.

Deviations from the bedrock topography model of Reber and Schlunegger (2016) and our solutions are not only visible at the scale of an entire profile, but also at the local scale. For the Bern1 profile, for instance, two drillings are situated either close to our profile with a distance < 100 m (drilling labelled with 1 on Fig. 8c) or slightly further away (> 200 m, drilling labelled with 2 on Fig. 8c). Both of them reached the bedrock at deep levels but we could not model a corresponding gravity signal. Indeed, the application of the PRISMA routine suggests that at a broader scale the bedrock is much shallower in this area (Fig. 8). While our modelling approach is not capable of fully solving this conflicting information, we tentatively consider (similar to the Marzili case, Bern4 profile in Fig. 12b) an interpretation where the shallow and flat bedrock shoulder inferred from our gravity survey reflects the occurrence of a bedrock ridge that is dissected by an inner gorge, which is too narrow to be detected by our survey but was obviously penetrated by a drilling.

#### Assignments of densities to the Molasse bedrock and the Quaternary fill of overdeepenings

Because gravity values provide information on the total mass of an object such as an overdeepening fill, we used bulk density values of the target sediments without lithofacies-specific density variations as presented by e.g., Schwenk et al. (2022a). In a sensitivity analysis, accomplished to assess the bulk density of the Quaternary fill of the Gürbe and the Aare overdeepenings (Sect. 11 on Fig. 1), Bandou et al. (2022) showed that the variations in the gravity signals measured directly above the structure in question depends on the volume and on the bulk density, and that such relationships are non-linear: For a small volume of sediments (e.g. expressed by a thickness of 50 m), a difference of 100 kg/m^3^ in the bulk density assigned to the sediments would yield a gravity signal of 0.2 mGal (Figure S4b in Bandou et al., 2022). A similar difference but with a 300 m-thick fill would yield a 1 mGal gravity signal. If these bodies would be placed at greater depths, then the signals would be lower as gravity decreases exponentially with distance (Li and Götze, 2001). Because the maximum depth of the target overdeepenings are in the range of several hundred meters (Preusser et al., 2011), we constrained the bulk density values to both the bedrock and the Quaternary sediments as precisely as possible either through (i) Nettleton profiling along the Gürbe-Belpberg-Aare profile (Figure S2 in Bandou et al. (2022), (ii) modelling the maximum gravity values of an overdeepening fill (see Figure S4b in Bandou et al., 2022), (iii) modelling well constrained structures underlain by Quaternary sediments of known ages (e.g., NE margin of the Bremgarten profile, Figs. 5, 7), and (iv) considering published information on lithofacies-specific density values (Schwenk et al., 2022a), but using an average value of such data. We converged such information to characterize sedimentary packages tens to hundred meters thick with bulk density values, which appears as a suitable approach to reconstruct the overarching architecture (e.g., the stacking of multiple storeys) as documented in Kissling and Schwendener (1990), Bandou et al. (2022) and in this contribution.

#### Effects related to the spatial resolution of the gravity survey

Most of the main anomalies disclose wavelengths that are several hundred meters to a few kilometres long. Such signals can be easily identified by a few gravity stations (e.g., ten). At locations where we aimed at obtaining more details about smaller-scale features such as plateaus and knickpoints, we had to reduce the spacing between the stations to < 100 m. In order to identify short-wavelength structures at greater depths (such as inner gorges), the signals of such features have to be recorded by several stations mainly in order to differentiate them from effects caused by shallow structures (such as a underground garage, cellars etc.). Yet in cases where the volumes of deep structures are not large enough, our method will not be able to conclusively detect them. This is documented with the Bern2 profile, where the identification of such a gorge has mainly been motivated through drilling information.

### Multi-storey cross-sectional architecture

**Fig. 13:**
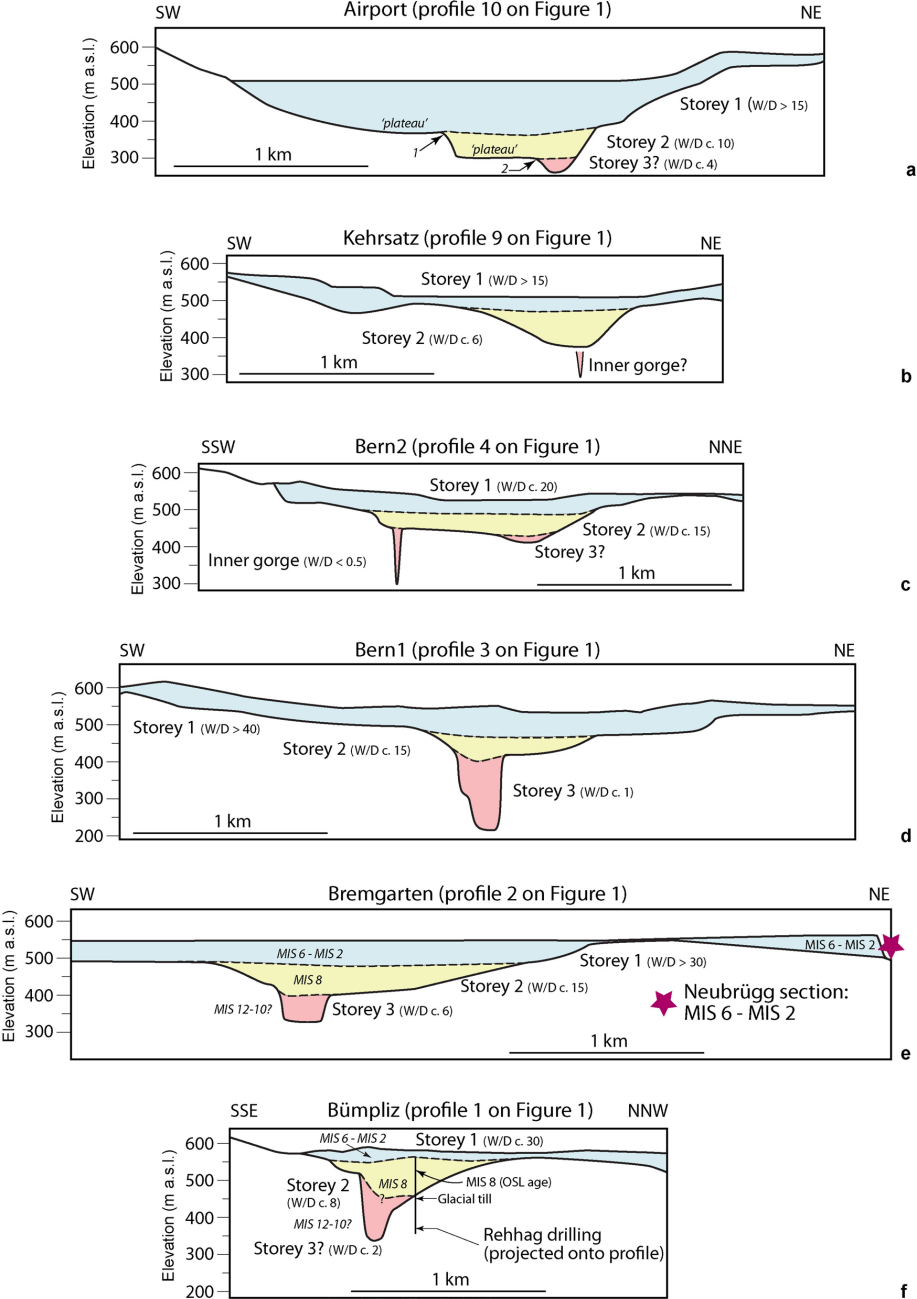
Proposed architecture of **a** the Airport profile, **b** the Kehrsatz profile, **c** the Bern2 profile, **d** the Bern1 profile, **e** the Bremgarten profile, and **f** the Bümpliz profile. The profiles can be categorized into 2–3 storeys, each of which have a flat base and steep lateral flanks. See text for further explanations

The residual anomalies and the modelling results show the occurrence of plateaus or break-in-slopes along nearly all profiles. This is visible, for instance, along the Bremgarten profile where a plateau and break-in-slopes in the residual anomaly patterns occur at − 1.0 and − 2.0 mGal on the SW side (break-in-slope denoted as kink-1 and kink-2 on Fig. 7b). Modelling shows that these correspond to depth levels where the cross-sectional shape of the overdeepenings change from a plateau towards a steeply dipping flank along the profile, and where in some cases the bulk density of the overdeepening fill increases (e.g., Fig. 7c). For the Gürbe-Belpberg-Aare profile (Sect. 11 on Fig. 1), Bandou et al. (2022) could document that such plateaus and kinks in the residual anomaly and modelling results correspond to depth levels where drillings (e.g., Brunnenbohrung and RB 9201, Fig. 1) document the occurrence of a till. This then allowed Bandou et al. (2022) to propose that such a plateau could have been formed by glacial carving, thereby marking the base of a storey that is overlain by a sedimentary sequence. Following this logic, we categorize the cross-sectional geometries of our profiles into storeys. We place the base of such a storey where a ‘plateau’ or a flat segment in a cross-section abruptly ends, giving way to a steeply dipping lateral boundary, such as at locations indicated with 1 and 2 in Fig. 13a. This allows us to categorize each profile into a succession of at least 2–3 storeys (Table 1), where the upper levels have larger width/depth ratios than the lower ones. Accordingly, we place the base of storey 1 at an elevation between 350 and 400 m a.s.l. along the Airport profile (Fig. 13a). Towards Kehrsatz, the elevation of this base rises to c. 500 m a.s.l. and remains at this altitude farther north (Fig. 13; Table 1). Likewise, the base of the inferred storey 2 is situated slightly above 300 m a.s.l. along the Airport profile (Fig. 13a), from where it rises to c. 350 m a.s.l. at Kehrsatz and then finally to an elevation of 400 m a.sl. in the profiles farther north (Fig. 13). A third unit (storey 3) with low width/depth ratios < 6 could be identified in nearly all profiles. This lowermost storey reaches a depth level of c. 300 m a.s.l. and appears to form a several tens of meters- to a few hundred meters-wide structure mainly beneath the Bern2 and Bern1 and possibly also at the base of the Bremgarten and Bümpliz profiles. The modelling results additionally show that the sedimentary infill of the uppermost storey 1 has a bulk density of 2000 kg/m^3^, whereas the underlying sedimentary sequences have a higher bulk density of 2150 kg/m^3^.

**Table 1:** Amplitudes of residual anomalies and morphometric properties of the overdeepening along the various profiles

Profile Name	Bümpliz	Bremgarten	Bern1	Bern2	Bern3	Bern4	Wabern1	Wabern2	Kehrsatz	Airport
Profile Nr. on Fig. 1	1	2	3	3	5	6	7	8	9	10
Width of main trough (m)	1250	3860	2450	2080	2000	1700	1600	3900	2380	2500
Maximum residual anomarly of main trough (mGal)	− 1.7	− 2.5	− 2.5	− 1.6	− 1.8	− 1.4	− 0.8	− 1.3	− 1.7	− 3.3
Base of storey 1 (m a.s.l.)	550	500	500	500	–	–	–	–	500	350–400
Base of storey 2 (m a.s.l.)	450–550	400	400	400	–	–	–	–	350	300
Base of storey 3 or inner gorge (m a.s.l.)	330–450	300–350	200–250	< 300	–	–	–	–	300?	250–300
Width/Depth ratio of storey 1	30	> 30	> 40	20	–	–	–	–	> 15	> 15
Width/Depth ratio of storey 2	8	15	15	15	–	–	–	–	6	10
Width/Depth ratio of storey 3	2	6	1	< 0.5	–	–	–	–	–	4
With/Depth of entire trough	5	19	7	8	–	–	–	–	12	10
Maximum thickness of the fill (m)	218	205	300	200	–	–	–	–	130	240
Density of fill, storey 1 (kg/m^3^)	2000	2000	2000	2000	–	–	–	–	2000	2000
Density of fill, storey 2 (kg/m^3^)	2150	2150	2150	2150	–	–	–	–	2150	2150
Density of fill, storey 3 (kg/m^3^)	2150	2150	2150	2150	–	–	–	–	–	2150
Slope angle of eastern margin storey 1 (°)	38.7	7.6	4.6	16.7	–	–	–	–	13	10.7
Slope angle of western margin storey 1 (°)	8.4	7.5	26.6	11.3	–	–	–	–	13.8	18.4
Slope angle of eastern margin storey 2 (°)	82.4	17.5	35.5	13.5	–	–	–	–	15.5	74.1
Slope angle of western margin storey 2 (°)	15.5	13.5	14	24.8	–	–	–	–	37.6	54.5
Slope angle of eastern margin storey 3 (°)	76	83.9	65.8	2.8	–	–	–	–	–	5.1
Slope angle of western margin storey 3 (°)	20	11.4	87.1	11.3	–	–	–	–	–	29.7

See Fig. 13 and text for further information

### Change of cross-sectional geometries of overdeepenings from upstream to downstream

#### Upstream of Bern: U-shaped and deep trough with a multi-storey architecture

Along the Gürbe-Belpberg-Aare profile (Sect. 11 on Fig. 1), Bandou et al. (2022) inferred the occurrence of overdeepenings (Gürbe and Aare) with a two-storey architecture where each storey has a typical U-shaped cross-sectional geometry with steep lateral flanks and a flat base (width/depth ratios between 5 and 12). Whereas the Aare overdeepening reaches a depth of c. 300 m a.s.l., the Gürbe trough is apparently much shallower (c. 360 m a.s.l.).

Approximately 4 km farther downstream, the overdeepening’s geometry is still wide (c. 3 km), asymmetric and mainly U-shaped (Fig. 13a; Table 1) as disclosed by the Airport profile, and a two- to possibly three-storey structure can additionally be identified. According to the modelling results, the depth of the valley at the Airport site reaches a level between c. 270–310 m a.s.l. (depending on the assignment of bulk densities to the overdeepening fill, see Fig. 11). This is slightly deeper than upstream in the Aare valley. The Kehrsatz profile (Sect. 9 on Fig. 1), which is situated an additional 1.8 km farther downstream, shares similarities to the Airport profile (Fig. 13b; Table 1). It it is made up of two storeys, each of which are U-shaped, asymmetric, but narrower than farther upstream, and we additionally identified a shallow (40 m deep) side channel on the SW side. Although our modelling infers the occurrence of a flat base situated at 390 m a.s.l. and thus at much shallower levels than at the Airport, we cannot exclude that one or multiple deep and narrow channels (storey 3?) occur at deeper levels (Fig. 13b).

#### City area of Bern: U-shaped, shallow trough with a multi-storey architecture on top and inner gorges below

The situation then changes between the Wabern2 (Sect. 8 on Fig. 1) and the Bern2 profiles (Sect. 4, Fig. 1) c. 1.6 kms farther downstream of Kehrsatz. There, we observe a much wider and shallower trough such as at Wabern2, where the main depression is characterized by a maximum residual anomaly of only − 1.3 mGal. This indicates that the bedrock reaches shallower levels than farther South such as at the Airport and Kehrsatz sites where the maximum residual anomalies are − 3.3 mGal and then − 1.7 mGal, respectively (Figs. 10, 11; Table 1). Downstream of Wabern2, the bedrock apparently becomes even shallower and the valley gets narrower at Wabern1, and then starts getting deeper again towards Bern4 (profile 6 on Fig. 1) and farther North, yet the overall U-shaped cross-sectional geometry is maintained. However, the Bern4 section crosses the site of the Marzili drilling (Fig. 12b) where the bedrock was encountered at a depth of 237 m a.s.l. We were not able to detect a clear corresponding gravity signal above the Marzili drilling site. This is the main reason why we suggest that the overdeepening beneath the Bern city area is characterized by a shallow bedrock ridge situated several tens of meters beneath the current surface, dissected by one or possible multiple inner gorges where the Quaternary mass was too small to be detected by our gravity survey. Yet, we propose that the occurrence of such an inner gorge can be confirmed by the Bern3 profile (Sect. 4 on Fig. 1, and Fig. 12a) and particularly along the Bern2 profile (Sect. 3 on Fig. 1, and Figs. 9 and 13c) where we detected secondary anomalies beneath the main wavelength depression. At Bern2, for instance, we observe two storeys with U-shaped cross-sectional geometries (width to depth ratios > 15) similar to the other profiles in the Bern area. These storeys have steep flanks and a flat base that slightly dips towards the NE. In addition, also at Bern2, we do observe a secondary anomaly on the SW flank that has a short wavelength, for which the maximum amplitude is the same as the main trough (Fig. 9). Modelling shows that such a condition can only be reached if this secondary anomaly is caused by a narrow and deep structure.

#### Downstream of Bern: U-shaped trough with a multi-storey architecture on top and a V-shaped channel below

North of Bern at a c. 1 km downstream distance of the Bern2 profile, the main overdeepening maintains the two-storey architecture. In particular, the upper part of the Bern1 and Bern2 profiles share a similar overall U-shaped, two-storey geometry in cross-section (Fig. 13c, d). The lowermost part (storey 3), however, is narrow and V-shaped with a width/depth ratio that is lower than 1. Interestingly, if we consider the main direction of the overdeepening as reference, then the location of the deepest site at Bern1 is aligned with the location where we identified the inner gorge farther upstream. If such a spatial correlation is appropriate, then it is possible that the lower V-shaped part of Bern1 is the downstream continuation of the inner gorge. Interestingly, the model predicts a base at an elevation of c. 230 m a.s.l., which is nearly equivalent to the depth at which the Marzili drilling reached the bedrock (Figs. 8c and 12b). Yet a PRISMA model of the residual anomaly pattern implies that a bedrock at a deeper level would also be possible (Additional file 4: Appendix D.4). However, such an interpretation conflicts with the information offered by a drilling situated at < 20 m distance of our gravity station, for which we measured the maximum anomaly. There the bedrock is apparently shallower (274 m a.s.l) than what the modelling implies. We use this observation to infer a complex pattern for the V-shaped part of the inner gorge, which includes the occurrence of meanders and side channels (please see Sect. 5.1).

Along the Bremgarten profile (Fig. 13e) and upon approaching the distal termination of the main Aare overdeepening, the uppermost part (storey 1) has a Quaternary fill with a similar thickness and shape as the corresponding part farther upstream, but it is wider. The bottom part appears as a composite of an upper storey that tends to be more U-shaped (storey 2), and a basal section which is much narrower (Fig. 13e). Upon modelling, we assigned a minimum depth of 340 m a.s.l. to the base of the Bremgarten section. This depth is shallower than farther upstream, but we cannot exclude the possibility where the inner gorge, which we identified based on gravity data (Bern2 profile, Fig. 9) and drilling information (Marzili drillhole in the Bern4 profile, Fig. 12b) continues as far north as Bremgarten. Finally, the Bümpliz side channel has a similar architecture in the sense that the upper part (storey 1) is wide and U-shaped, while the lower part displays a V-shaped cross-sectional geometry (width to depth ratio of c. 2), reaching a depth level of 340 m a.s.l.

#### Bedrock ridge with one or multiple inner gorges buried beneath Bern, and a modern analogue

**Fig. 14:**
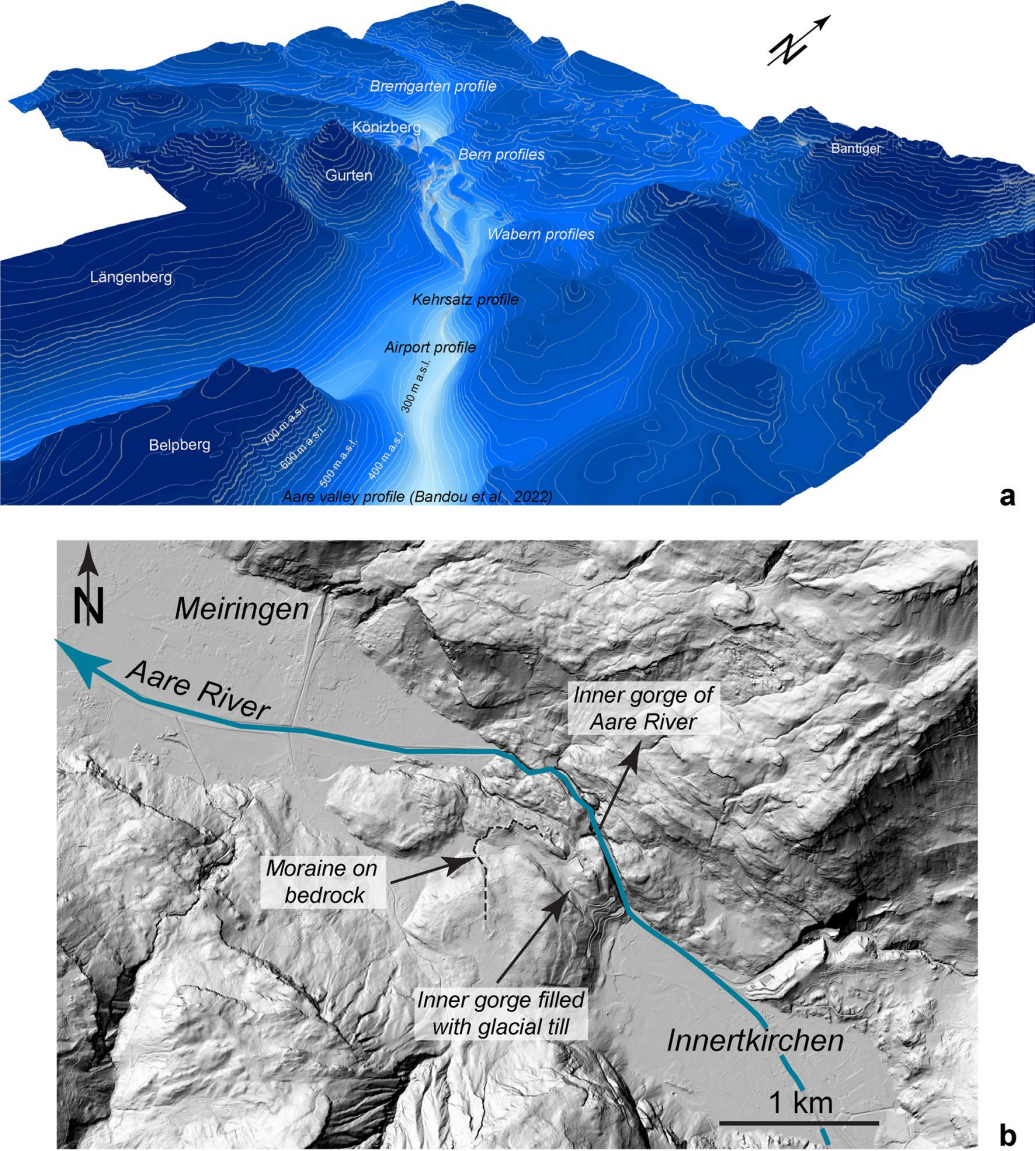
**a** Perspective view looking downstream along the thalweg of the Aare main overdeepening. Note that the valley is U-shaped upstream and then transitions in a cross-section characterized by a bedrock riegel (currently buried by several tens of m-thick Quaternary sediments), which is dissected by one (or multiple) V-shaped inner gorges. Contour lines are shown for every 20 m elevation. The elevation of c. 500 m a.s.l. corresponds to the lowest surface elevation encountered in this survey.** b** LidarDEM of the region surrounding the current Aare gorge downstream of Innertkirchen at c. 2′658′000/1′174′000 (Swiss coordinate system), which is situated approximately 75 km upstream of Bern. The bedrock ridge is c. 160 m high and dissected by multiple inner gorges. Most of them are filled with glacial till. Currently, the Aare River flows through an inner gorge on the NE margin of the bedrock ridge. We consider the area surrounding the Aare gorge between Innertkirchen and Meiringen as a modern example of the bedrock topography beneath the city of Bern

**Fig. 15:**
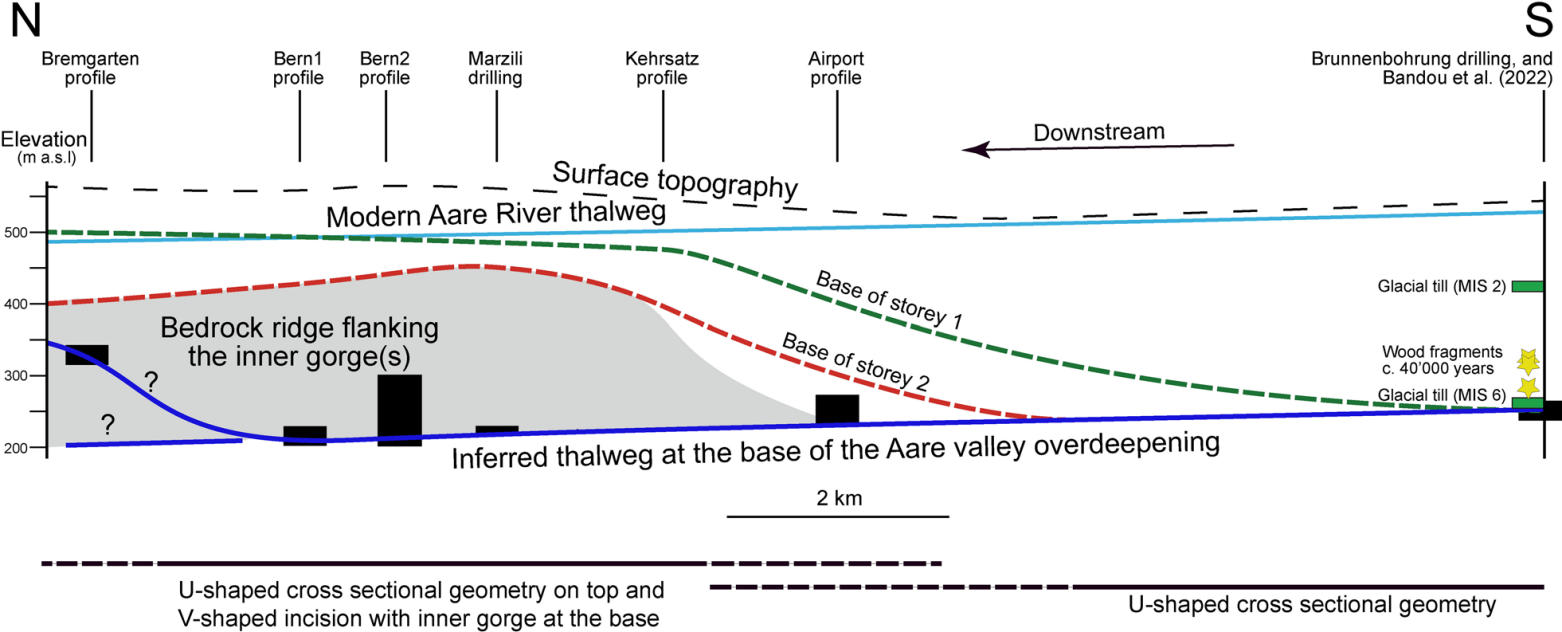
Schematic section from upstream to downstream showing the modern surface topography as black dashed line, the thalweg of the modern Aare River (pale blue), the base of storeys 1 and 2, and the age of the Quaternary fill at the Brunnenbohrung drillsite (see Fig. 1 for location of drilling). The figure also shows the bedrock ridge beneath the city of Bern (grey area) and the inferred thalweg at some time before the MIS 8. The black rectangles indicate the uncertainty associated with the assignment of the corresponding depth. This uncertainty is derived from modelling

As outlined above, the main Aare overdeepening evolves from a U-shaped cross-sectional geometry farther upstream towards a setting where the basal part is dominated by a V-shaped structure upon approaching Bern (Figs. 13, 14a). This latter structure appears to narrow to the extent that mass is getting so small that a corresponding gravity signal cannot be identified with our approach. However, we note that drillings might eventually penetrate such structures as documented by the Marzili borehole. Modern analogues of such a bedrock feature could eventually be found between Innertkirchen and Meiringen c. 75 km upstream of Bern (Fig. 14b). There the Aare River cuts through a bedrock riegel and flows in a V-shaped inner-gorge where the width ranges from 40 m to < 2 m in some locations (Fig. 14b) and where the depth of incision is > 140 m (Hantke and Scheidegger, 1993). If translated to the Bern situation, then the equivalent of the bedrock ridge is situated right beneath the city area of Bern below c. 50 m of the modern surface (Fig. 15; north of Kehrsatz). Furthermore, the stoss-side of the bedrock ridge is likely to be situated between the Wabern2 and Wabern1 profiles, and the lee-side gives way towards the Bremgarten profile. Similar to the current Aare gorge at Meiringen, we infer that the thalweg was most likely connected from upstream to downstream. We propose this interpretation because the maximum depths, both encountered by drillings and recovered through modelling, are situated at nearly the same elevation. Whether the thalweg will shallow upwards approaching the Bremgarten profile (Fig. 15) is still the topic of further ongoing research (see also above). Moreover, own field inspections showed that the Aare gorge south of Meiringen displays features such as glacial mills, which point towards an origin beneath a glacier. In addition, the current depth of the bedrock beneath the Aare gorge in Meiringen is not known. Therefore, we suggest that the inner gorge system beneath Bern was also formed in a glacial environment.

### Inferred chronological framework

North of Bern, published chronological constraints were presented for the Neubrügg section situated on the NE end of the Bremgarten profile (Fig. 13e). For this section, Lüthy et al. (1963) proposed a stratigraphic framework with what they considered as a ‘Riss’ moraine at the base, followed by the Karlsruhe Schotter, and a ‘Würm’ moraine on top. The ‘Riss’ moraine is additionally overlain by fine-grained sediments and a gravel layer. Pollen fragments in the sand layer could suggest a transition from a possibly warm to a cool-mountainous climate, which we tentatively consider as pointing towards the end of MIS 5e thereby following Lüthy et al. (1963). Given this information, we assign the ‘Riss’ moraine of Lüthy et al. (1963) to MIS 6, and the ‘Würm’ moraine to MIS 2, but we acknowledge that such age interpretations need to be constrained by absolute dating methods in the future. Accordingly, the base of storey 1 on the NE margin of the Bremgarten profile might have an age of MIS 6.

For the Bümpliz profile (Fig. 13f), Schwenk et al. (2022a) used OSL (optically stimulated luminescence) signals to date the topmost sediments encountered in the Rehhag drilling to a minimum age of 250′000–300′000 yrs, which could correspond to MIS 8 (Hagenholz glaciation) and possibly older. As illustrated in Fig. 13f and disclosed by gravity modelling, the drilled sequence is most likely part of storey 2 and possibly 3. In addition, this sedimentary sequence has a bulk density of c. 2150 kg/m^3^, which is the average of the density values measured along the Rehhag drillcore (Schwenk et al., 2022a). Because this sequence was deposited during MIS 8 or possibly before, it experienced a glacial loading and thus a compaction during at least two major glaciations (i.e., during MIS 6 and MIS 2). Apparently, the material encountered in the Rehhag drilling appears to be denser than the sedimentary fill of storey 1 in the Bremgarten profile. Indeed, these sediments were most likely deposited between MIS 6 and MIS 2 and thus experienced a compaction (Bini et al., 2009) during one major glaciation only (i.e., during MIS 2).

Approximately 10 km farther South, wood fragments were encountered in the Brunnenbohrung (site illustrated in Fig. 1) at drilling depths between 200 and 230 m and thus a few tens of meters above the base of the Aare main overdeepening (Bandou et al., 2022; Fig. 15). The material, which was embedded in lacustrine sediments, was ^14^C-dated to c. 40′000 years BP (Kellerhals and Häfeli, 1984), which is MIS 3. The lacustrine sequence itself is overlain and underlain by a glacial till at c. 100 and > 250 m drilling depths, respectively (Fig. 15). Bandou et al. (2022) used these constraints to assign a depositional age between MIS 6 and the Holocene to the fill of the main Aare overdeepening along the Belpberg-Aare profile (Sect. 11 on Fig. 1), and they assigned a bulk density of the 2000 kg/m^3^ to the entire sedimentary fill. These constraints allow us to tentatively correlate the sequence along the Belpberg-Aare profile to the fill of storey 1 farther downstream (Fig. 15).

### Implications for understanding the formation of overdeepenings in the region

If our tentative age assignments are correct, then the main overdeepened trough in the Aare valley (but also in the Gürbe valley according to Bandou et al., 2022) is filled with Quaternary sediments of which the majority of the material has an age that is MIS 6 and younger. These sediments rest on a depression that is mainly U-shaped in cross-section, and they are mainly found south of Bern where they fill nearly the entire trough (Bandou et al., 2022). Suites for which we assigned ages of MIS 8 and older are the fill of incisions that are encountered beneath Bern as inner gorges and in the V-shaped lower sections farther downstream. Such a morphology indicates that upstream of Bern, glacial carving was the dominating mechanism to shape the bedrock depression (e.g., Moreau et al., 2012), and that could have overprinted a presumably V-shaped topography. We suggest that this possibly original and older topography is still preserved underneath the city area of Bern and farther downstream, and that it was formed through erosion by water. This water could have originated from glacial melt and circulated underneath a glacier during a glacial period, thereby causing the incision (e.g., Herman et al., 2011). Alternatively, a large supply of glacial meltwater towards the end of a glacial period (Cohen et al., 2023) could also have promoted a rapid downwearing of the Molasse bedrock, particularly at the ice margin (e.g., van der Vegt, 2012). Such a mechanism was invoked, for instance, to explain the breaching of the bedrock ridge at the Dover Strait and the carving of deep channels on the floor of the eastern English Channel (e.g., Collier, 2015; Benvenuti et al., 2017; Gupta et al., 2017; Lohrberg et al., 2022). We consider such a scenario not unlikely given the nearly continuous paleo-thalweg at the base of the overdeepening system beneath the city of Bern (Fig. 15). Accordingly, while large water fluxes during the aftermath of a major glaciation (Möhlin glaciation?) could have resulted in the V-shaped carving of the bedrock, the subsequent glaciations mainly resulted in the widening of the trough but not necessarily in a further deepening, at least in the Bern area.

## Conclusions

Our study shows that the framework developed in this paper, consisting of a gravity survey paired with high-precision elevation data (such as the GNSS and the SwissAlti3D 2 m-DEM) allows for a reconstruction of the cross-sectional geometry of overdeepened valleys. This data served as input for our 3D gravity modelling software PRISMA, the results of which allowed us to investigate the erosional mechanisms leading to the formation of these bedrock depressions. We presented a setup consisting of: (i) measuring gravity data along profiles perpendicularly to the overdeepening’s flanks and far beyond the limits of the overdeepenings to link with the regional gravity field; (ii) extracting the residual gravity anomalies from these profiles, and (iii) gravity modelling. We documented that such a strategy yielded information on the general cross-sectional shape of the depression. Yet an imaging of very narrow and deep structures such as inner gorges and side channels can be very challenging or nearly impossible in some situations. This is the case because the gravimetric signals of such structures are pushing the entire workflow and thus the method to its limits. Despite these hurdles, we were able to document how the cross-sectional geometry of the Aare main overdeepening changes downstream from a U-shaped morphology to V-shaped structures in the deeper part. We consider the U-shaped geometry as a response to the glacial carving during the most recent glaciations (MIS 6 and MIS 2), whereas available stratigraphic data imply that the age of the material filling the V-shaped lower sections could be MIS 8 or older. This has implications for our understanding of the erosional processes leading to the formation of these troughs. We thus envisage an origin by water dissection either underneath a glacier or during the aftermath of a major glaciation when large meltwater supply contributed to the fluvial downcutting into the Molasse bedrock. Strong evidence for the inferred water control on erosion is offered by the occurrence of inner gorges underneath the city of Bern, which underlie the main overdeepening. Subsequent glaciations resulted in a widening of the already existing trough without further deepening them particularly downstream of the Bern area.

### Supplementary Information

**Additional file 1: Appendix A.** Gravity data used as initial input for each profile upon modelling with the PRISMA routine. Gravity data used to calculate the Bouguer anomaly, and gravity stations that we considered to determine the uncertainties associated with the measurements of the gravity. Elevation and bedrock data for each gravity profile. This data was used as constraint for the modelling with the PRISMA routine.

**Additional file 2: Appendix B.** Drilling information, regional gravity stations and isolines used for each gravity profile.

**Additional file 3: Appendix C.** Projection of the data onto the profiles and rotation of the coordinates' axes so that the PRISM modelling could be performed.

**Additional file 4: Appendix D.** Profiles that were measured and modelled for this paper, and details on the modelling procedure that we applied to each profile.

## Data Availability

All data used for this paper can be downloaded from the appendices. DEMs are available from swisstopo. The bedrock and drilling information is available from the openly accessible database of the Canton Bern. The datasets generated and/or analysed during the current study are available in the BORIS repository, https://boris-portal.unibe.ch/.
