# Supplementary material for: Overdeepenings in the Swiss plateau: U-shaped geometries underlain by inner gorges
Source: Swiss J Geosci. 2023 Dec 5;116(1):19. doi: 10.1186/s00015-023-00447-y (PMC10698152; doi:10.1186/s00015-023-00447-y)
Supplement: Supplementary file 3 — Additional file 3: Appendix C. Projection of the data onto the profiles and rotation of the coordinates' axes so that the PRISM modelling could be performed. [file 15_2023_447_MOESM3_ESM.pdf]

Appendix C:
Projection of the data onto profiles and rotation of the axes for the PRISM routine

C.1 Projection of data onto profiles

Our gravity survey strategy relied on measuring gravity stations along a profile, which is oriented perpendicularly to the targeted tunnel valleys' flanks. The analysis along a profile is much more straightforward than the analysis of the data on a map. It starts with the estimation of the regional gravity gradient along a gravity profile, and ends with the comparison between the residual anomaly and the calculated gravity effect of the model (see main text). We exemplify the projection approach for the example of the Bümpliz profile (see main text and Appendix D for location and further information).

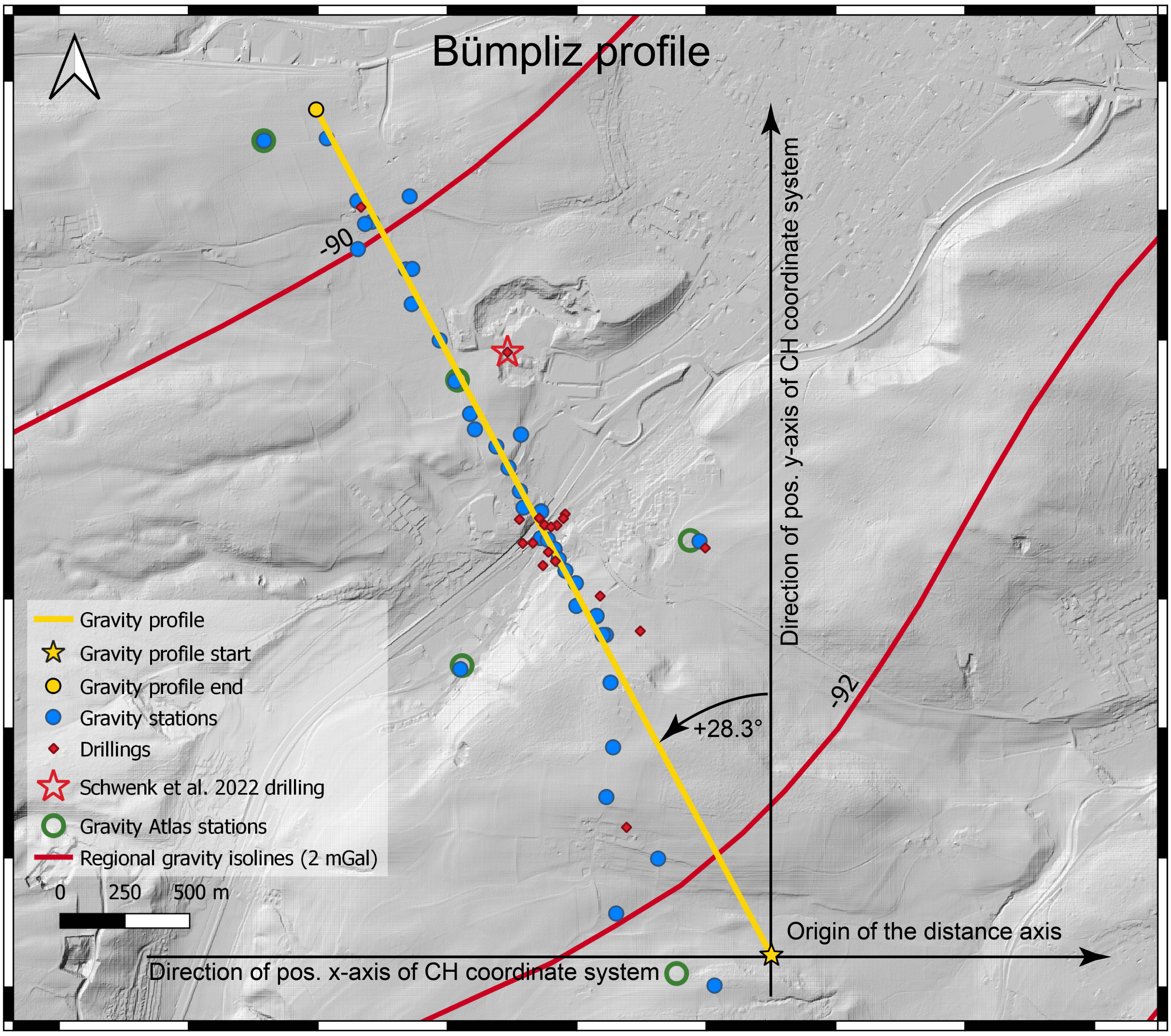

Figure C.1 *Definition of a profile's origin and the projection of points onto the profile. In the background, this map shows the 2 m LidarDEM (©swisstopo) of the area of the Bümpliz profile (see main text for location). The yellow line shows the gravity profile in the direction that is rotated 28 degrees counter-clockwise relative to the North. The blue dots are the newly measured gravity stations, the green circles represent the stations of the existing gravity atlas of Switzerland, and the red lines show the regional gravity gradient interpolated from the atlas stations with an interval of 2 mGal. The red dots indicate the location of drillings that have reached the bedrock, and the yellow star shows the position of the deepest drilling (Schwenk et al., 2022). The black axes give the directions of the Swiss coordinates system.*

The origin of a gravity profile is always located on its Southern/Western border. All points within a predefined lateral window from the profile are projected perpendicularly onto the profile (Figure C.1) and shown in the profile cross section (Figure C.2).

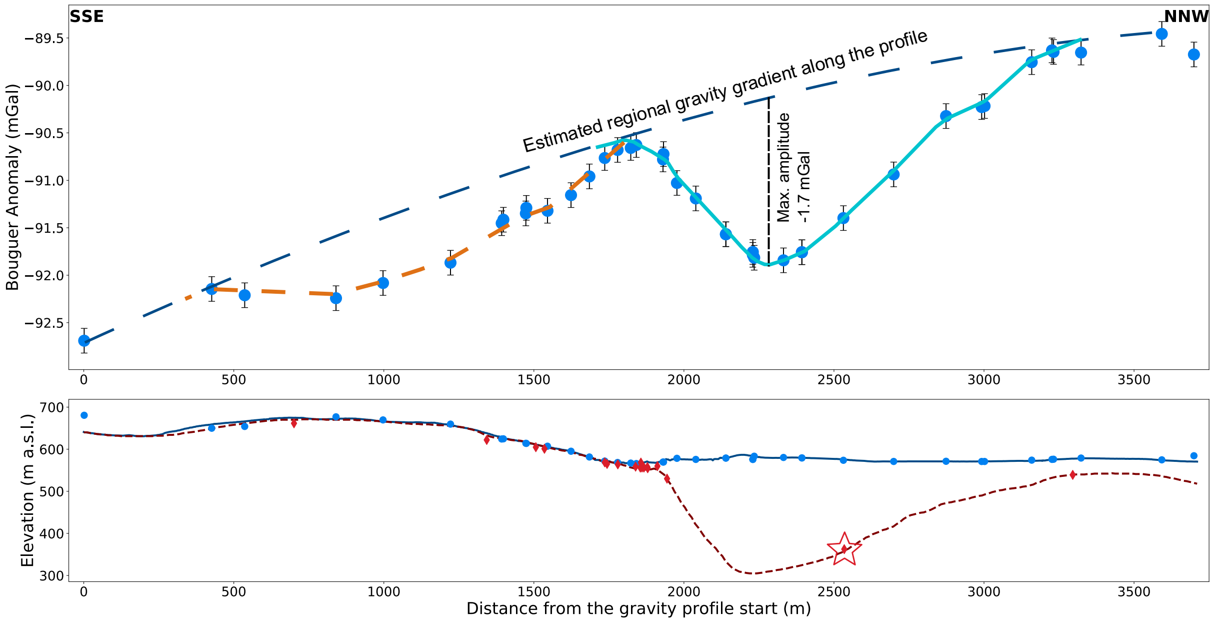

Figure C.2 *Bouguer anomaly, surface topography and bedrock topography model for the Bümpliz profile. Top: The blue dots denote the newly measured gravity stations and the blue broken line represents the regional gravity gradient. The light blue line indicates the local anomaly caused by the overdeepened valley, with a maximum anomaly of ca. 1.7 mGal. This main gravity anomaly caused by the tunnel valley is bordered by a separate anomaly on the SSE side, likely caused by the surface topography and near-surface bodies (broken orange line). They differ in their densities from the standard value of 2670 kg/mm3 that is commonly used to calculate the Bouguer gravity. Bottom: The surface elevation along the profile is represented by the blue line, taken from the 2 m DEM. The blue dots document the stations' elevations. The red broken line denotes the bedrock topography model by Reber & Schlunegger (2016) while the red diamonds indicate the drillings that have reached the bedrock, with the yellow star indicating the position of the deepest drilling (from Schwenk et al., 2022).*

In order to project the points (gravity point MP1 as an example) onto the profile and to calculate the distance D_{MP1} along this profile we use the formulae outlined below. First, we calculate the CH-coordinate distances in x- and y-directions between the MP1 and the start of the profile (Figure C.2):

$Dx_{MP1} = x_{CHMP1} - x_{CHStart}$ and $Dy_{MP1} = y_{CHMP1} - y_{CHStart}$ (C.1)

Considering the direction of the profile being oriented counter-clockwise rotated by +28 degrees from the North (corresponding to the CH-coordinate system y-axis, see Figure C.1), we obtain for the distance along the profile (D_{MP1}):

$$D_{MP1} = -Dx_{MP1} * \sin(\alpha) + Dy_{MP1} * \cos(\alpha) \quad (C.2).$$

In our case, α is +28.3° as the profile direction is rotated anticlockwise from the North. In the case where the profile direction is rotated clockwise from the North and thus in the opposite direction, the value of the angle α must be given as negative in the formula C.2.

The projected data are the CH-coordinates of: (i) the bedrock model points (from Reber and Schlunegger, 2016), (ii) the locations of the gravity stations, (iii) the surface topography points, and if available of (iv) the profile cross points with the prism borders. Thus, the cross section may show the

bedrock model including the drill hole locations, the Bouguer gravity and regional gravity gradient along the profile in combination with the DEM surface topography (Figure C.2). Alternatively, after the gravity modelling has been conducted, the cross section may show the bedrock model of Reber and Schlunegger (2016) with the selected drill hole locations, the residual gravity and the calculated model gravity at each MP, the DEM surface topography and the profile cross sections for each of the prisms in the model (for an example see Figure C.7 below).

C.2 Definition of a rotated Local Coordinate System (LCS) for calculations with the PRISM routine

The sedimentary infill of the valleys is modelled using prisms. Due to the complex geometry of overdeepenings, for 3D gravity modelling along profiles we can either use up to a few dozen horizontally stacked prisms, all of which follow the direction of the flanks of the valley, or we can use thousands of vertical prisms following the cells of the DEM, to best approach the geometry. Here we selected horizontally stacked prisms as they are more advantageous than vertical ones. Indeed, vertical prisms are more time consuming to adjust when tweaking the geometry, and they present a limitation concerning possible variations of the density with depth, which might be necessary if sedimentation compaction in response to glacial overburdens have to be considered. If one wants to use horizontally stacked prisms though, the prisms must be parallel to the valley flanks. This means that we need to define a local coordinate system (LCS) with the y-axis rotated to parallel the valley axis.

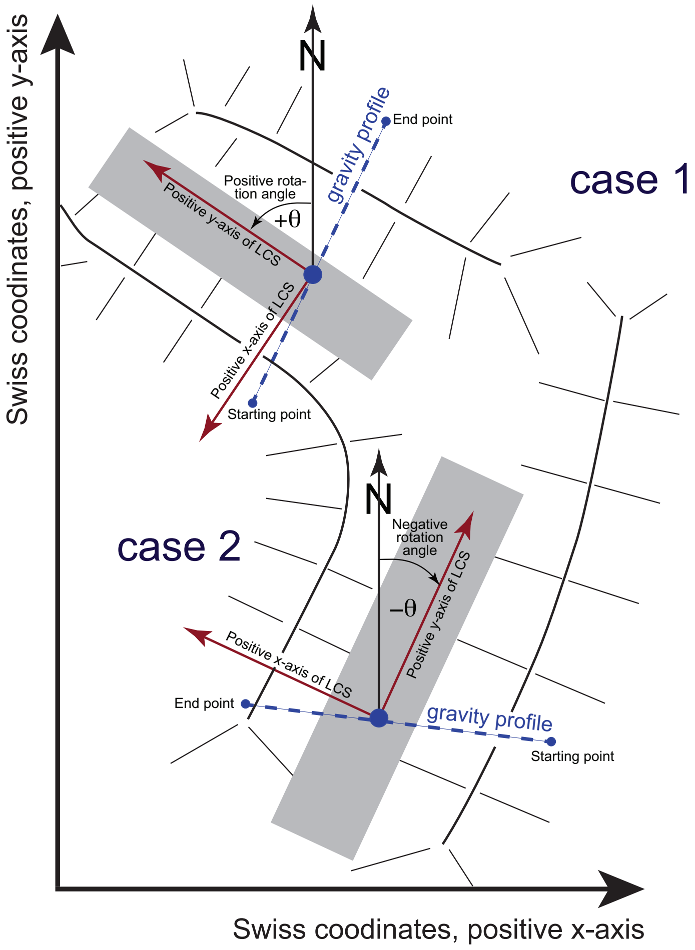

Figure C.3 *Two cases, which illustrate the orientations of the tunnel valley and thus the geometries of the prisms relative to the Swiss coordinate system. Because the PRISMA routine was designed for a right-handed cartesian coordinate system with z-axis towards the Earth's centre (Nagy, 1966), and since the analytical solutions for prisms are only applicable for a local cartesian coordinate system (LCS), we must define an LCS that is rotated*

relative to the Swiss coordinate system with the y-axis of the LCS paralleling the tunnel valley flanks. This requires a transformation of all data points from the Swiss coordinate system into the LCS.

The PRISMA routine (Bandou et al., 2022) allows to calculate the gravity effect of a series of prisms in freely distributed points (e.g., Nagy, 1966) and in a right-handed cartesian coordinate system with the z-axis oriented towards the Earth's centre. For a successful application of the PRISMA routine, we have to define a local right-handed coordinate system (LCS; Figure C.3) where the y-axis follows the general direction of the overdeepening, whereas the x-axis crosses the valley. Note that the gravity profiles cross the valleys but in general they will be oriented at an angle to the direction of the x-axis (Fig. C.3). We define the origin of the LCS to be located at the station where the maximum residual anomaly was determined, as this simplifies the subsequent forward modelling steps allowing to readily adjust the flanks on either side to reach a best fit with the measurements. The origin of this LCS has the following coordinates: $x_{CHorigin}, y_{CHorigin}, z_{CHmin}$

z_{CHmin} is defined as the elevation of the lowest MP along the profile. In order for PRISMA routine to work properly, the top level of the highest prism must be located below the lowest point for which the gravity effect is calculated.

The transformation from the Swiss to the LCS coordinate system is accomplished following the principle illustrated in Figure C.3. It considers first a translation of the origin (subtraction of the Swiss coordinates of the station at the origin from the other coordinates), yielding an intermediate coordinate system referred to LCS1, and then a rotation:

For any given point X_{CH}, Y_{CH}, Z_{CH}, the first intermediate coordinates (LCS1), which we get as a result of this translation are as follow:

$$x_{LCS1} = x_{CH} - x_{CHorigin} \; ; y_{LCS1} = y_{CH} - y_{CHorigin} \; ; z_{LCS1} = z_{CH} - z_{CHmin} \qquad \text{(C.3)},$$

The subsequent rotation of the coordinate system around the z-axis leading to the secondary intermediate coordinates (LCS2) is then accomplished as following (counterclockwise rotation with a positive angle, and clockwise rotation with a negative angle, see Figure C.3):

$$x_{LCS2} = x_{LCS1} * \cos(\theta) + y_{LCS1} * \sin(\theta) \qquad \text{(C.4)},$$

$$y_{LCS2} = -x_{LCS1} * \sin(\theta) + y_{LCS1} * \cos(\theta) \qquad \text{(C.5)}.$$

$$z_{LCS2} = z_{LCS1} \qquad \text{(C.6)}.$$

For the subsequent modelling with PRISMA we need a right-handed Cartesian coordinate system with the z-axis positive downward. Consequently, in the final step of the coordinate transform from the Swiss system to the LCS we need to flip both the z-axis and the x-axis, while the y-axis will not change. This then yields the final LCS:

$$z_{LCS} = z_{LCS2} * (-1) \qquad \text{(C.7)},$$

$$x_{LCS} = x_{LCS2} * (-1) \qquad \text{(C.8)},$$

$$y_{LCS} = y_{LCS2} \qquad \text{(C.9)}.$$

For these equations, $(x_{CH}, y_{CH}, z_{CH}, x_{CHorigin}, y_{CHorigin}, z_{CHmin})$ are the coordinates in the Swiss coordinates, $(x_{LCS1}/x_{LCS2}, y_{LCS1}/y_{LCS2}, z_{LCS1}/z_{LCS2})$ are the coordinates the first intermediate coordinate systems (LCS1/LCS2), and $(x_{LCS}, y_{LCS}, z_{LCS})$ are the final coordinates of the LCS that are used for the subsequent modelling with PRISMA. θ is the angle of rotation (Figure C.3). Consequently, in the case of the Bümpliz profile, this involves a rotation of the axis by -36° (in equations C.4 and C.5) from the North for all points to be considered upon using the position of the maximum anomaly along the gravity profile as the origin of the LCS (Figure C.4).

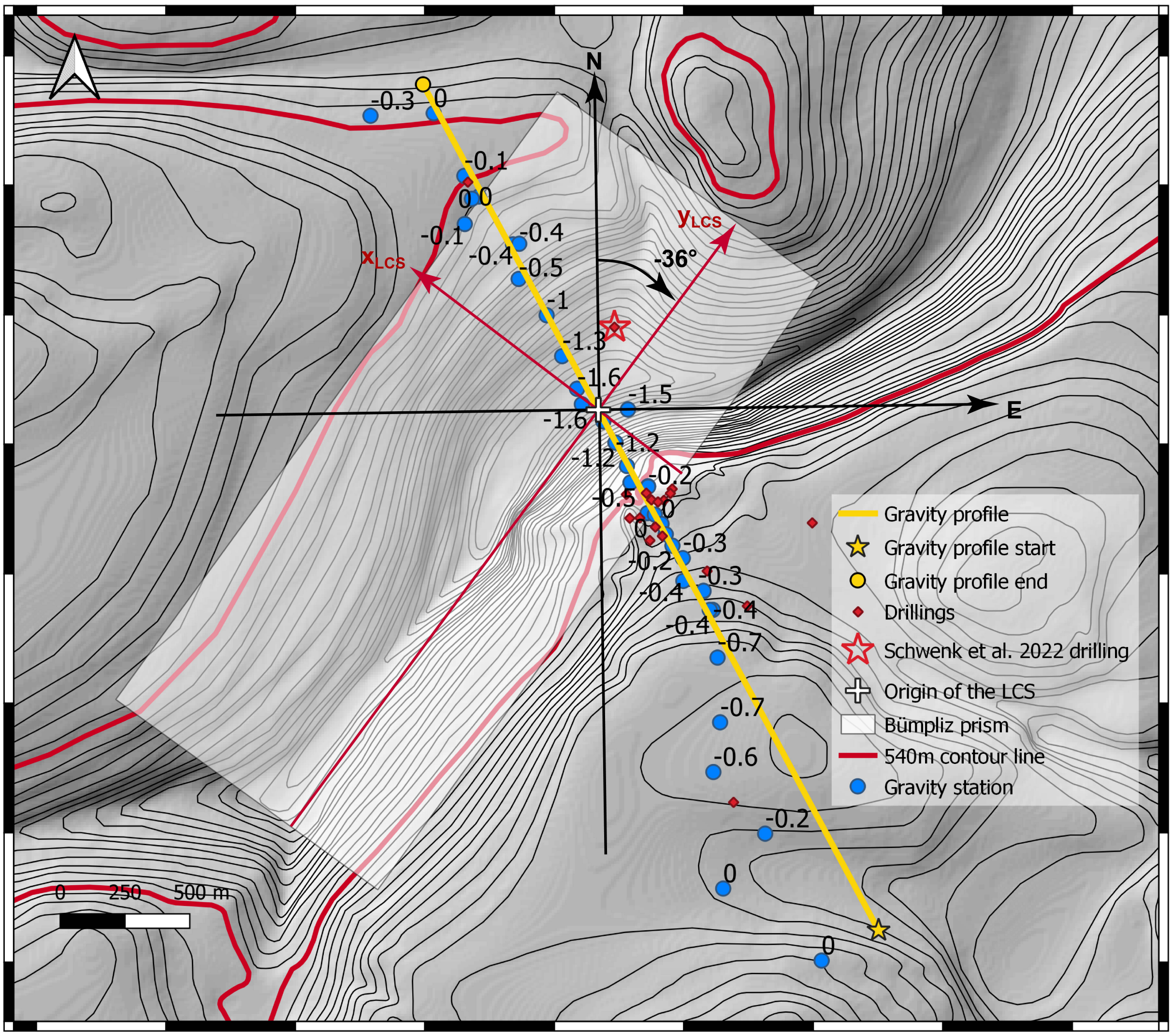

Figure C.4 *Map of the bedrock model from Reber and Schlunegger (2016) along the Bümpliz gravity profile area, showing the angle and axes that were used for the rotation of the gravity data, the drilling locations and the prism's coordinates. The blue dots are the gravity stations with the numbers indicating the residual gravity anomaly in mGal, the red diamond are the drillings. The white star denotes the origin and the red lines indicate the axes of the LCS. The Swiss coordinates system is represented by the black lines. Following the translation, a rotation of -36° and then a flipping of the x- and z-axes are needed to transform the points coordinates from the Swiss system into the LCS.*

C.3 Calculation of prism intersection points along the profile

During gravity modelling with the PRISMA routine we define a number of prims by their 8 corner points directly in the LCS. Once the gravity effect of the ensemble of prisms constituting a 3D model has been calculated for all MPs, we would like to display the calculated gravity in comparison with the observed residual gravity along the profile and in the same context we want to visualize the geometries of the prisms along the profile cross section. For this purpose, we need to calculate the intersection points between the prisms and the profile.

The LCS coordinates of the points at the intersections are calculated as outlined in the following. Here, we employ the line equation:

$$y = a * x + b \qquad \text{(C.10),}$$

where (x, y) are the coordinates and a and b are coefficients.

We determine a using two known points of our segments:

$a = \frac{y_{end} - y_{start}}{x_{end} - x_{start}}$ (C.11).

For the Bümpliz profile, $a = 0.48$. Then the b coefficient can be obtained through:

$b = y_{end} - a * x_{end}$ (C.12).

The value for the gravity profile is $b = 0.61$.

Once the a and b coefficients have been determined, the intersection between the gravity profile and the prisms' borders can be determined in a simple way, as the prisms' sides are always parallel to the LCS axes. If the crossing border is parallel to the y-axis, then only the *y* coordinates have to be determined, because the *x* coordinate is constant:

x_{prism} is constant, $x_{intersection} = x_{prism}$ and $y_{intersection} = a * x_{prism} + b$ (C.13).

Likewise, if the crossing border is parallel to the x-axis:

y_{prism} is constant, $y_{intersection} = y_{prism}$ and $x_{intersection} = \frac{y_{prism} - b}{a}$ (C.14).

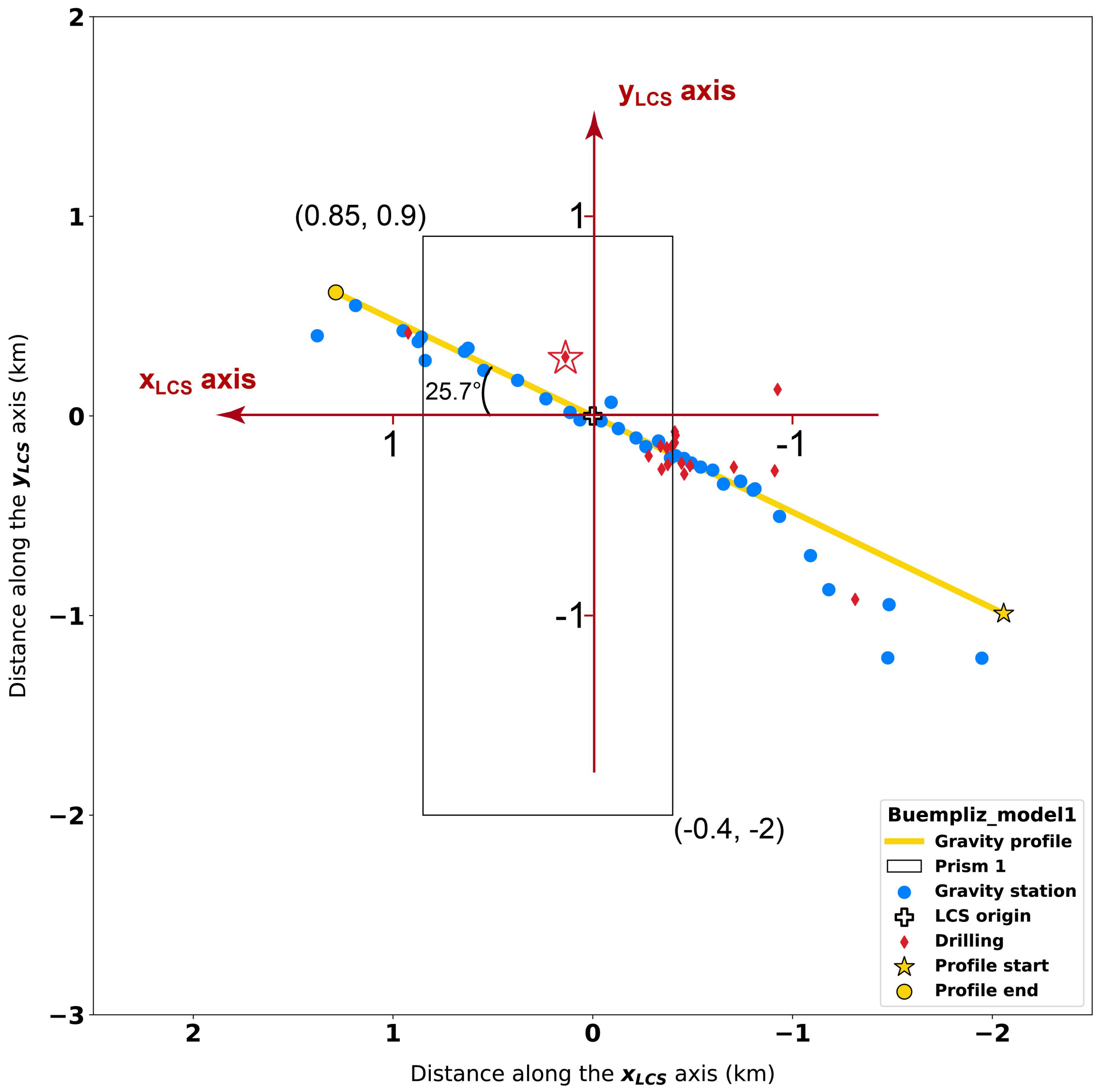

Figure C.5 *Map view of drillings (red diamonds), gravity stations (blue dots), gravity profile (yellow line) and drilling of Schwenk et al. (2022, yellow star) in local coordinate system (LCS). Corner coordinates of top prism (light grey) are given in km. The figure also shows a secondary prism (dark grey) representing the bend in the tunnel valley according to Reber and Schlunegger (2016). Int1 and Int2 are the LCS coordinates of the intersections between the top prism and the gravity profile. The white plus marks the origin of the LCS.*

In the case of the Bümpliz profile, for each prism x_{prism} is constant. We therefore determine the following coordinates for the intersection of the gravity profile and the borders of the top prism (Figure C.5): Int1 (-500, -240) and Int2 (700, 337.45), for the western and eastern borders respectively.

C.4 Situation where the gravity profile and the x-axis of the LCS have a large angle between them

All of our data is projected onto the gravity profile, as we measured and calculated the gravity anomaly along it (see Bremgarten, Bern-1, Bern-2, Kehrsatz and Airport profiles). However, for profiles that are oblique to the long axis of the target overdeepening where **ɣ** is thus >5° (as is the case for the Bümpliz profile), such a geometric configuration becomes a problem. This is illustrated in Figure C.7. The MP 1030, for instance, would appear on top of the prism upon modelling if projected perpendicularly onto the profile (purple line, Figure C.7), while in reality it should be aside of it. Therefore, to avoid such a situation particularly for profiles with a large **ɣ** angle (>5°), the points are projected following the LCS y-axis and therefore parallel to the prisms' boundaries (green line on Figure C.7) onto the gravity profile. This allows to show the position of the data relative to the prisms.

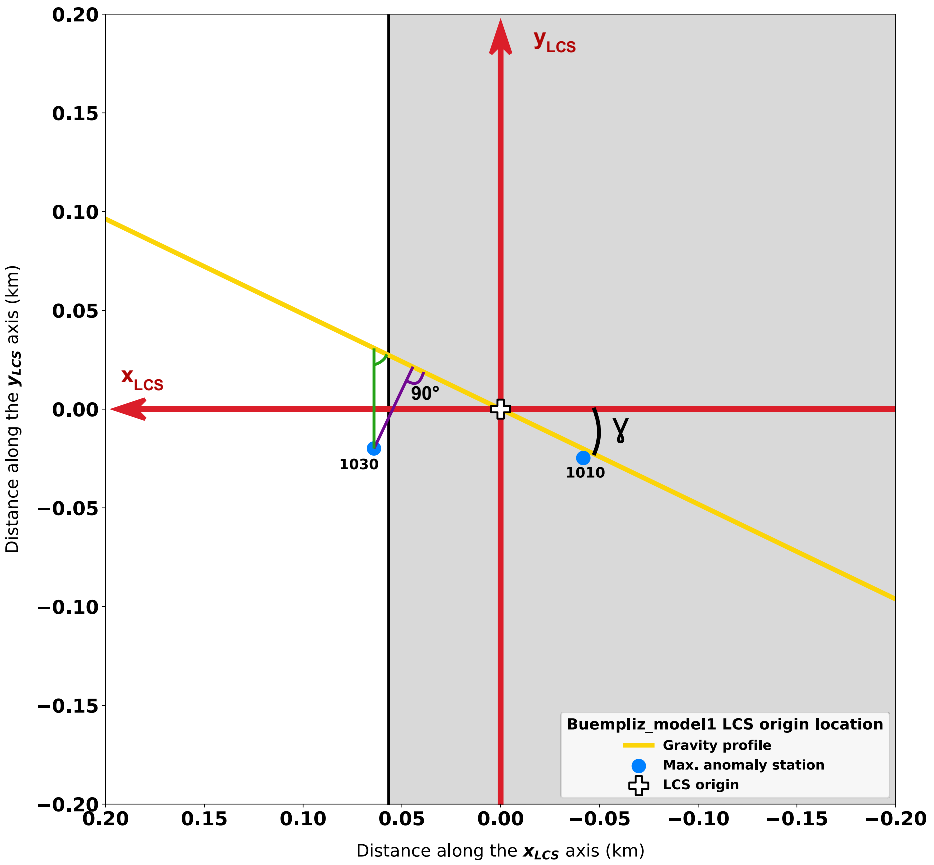

Figure C.6 *Close-up view from the site where the LCS origin is located for the case of the Bümpliz profile (taken from Figure C.5). It shows the LCS origin indicated by the white cross, the gravity stations near the LCS origin with the maximum anomaly marked by the blue dots, the gravity profile represented by the yellow line, and the LCS axes in red. The black line and the light grey background indicate the position of the side of one of the prisms used during modelling. The angle between the gravity profile and the* x_{LCS} *axis is referred to as* **ɣ.** *The purple line shows how the projection is done for all profiles where the* **ɣ** *is less than 5° (e.g., for the Bümpliz, Bern-1, Bern-2, Kehrsatz and Airport profiles), which is perpendicular to the gravity profile, while the green line shows a projection on the gravity profile parallel to the long axis of the prism (for Bümpliz). The points' coordinates are then expressed in the LCS coordinate system where the LCS x-axis is oriented perpendicular to the orientation of the tunnel valley thalweg.*

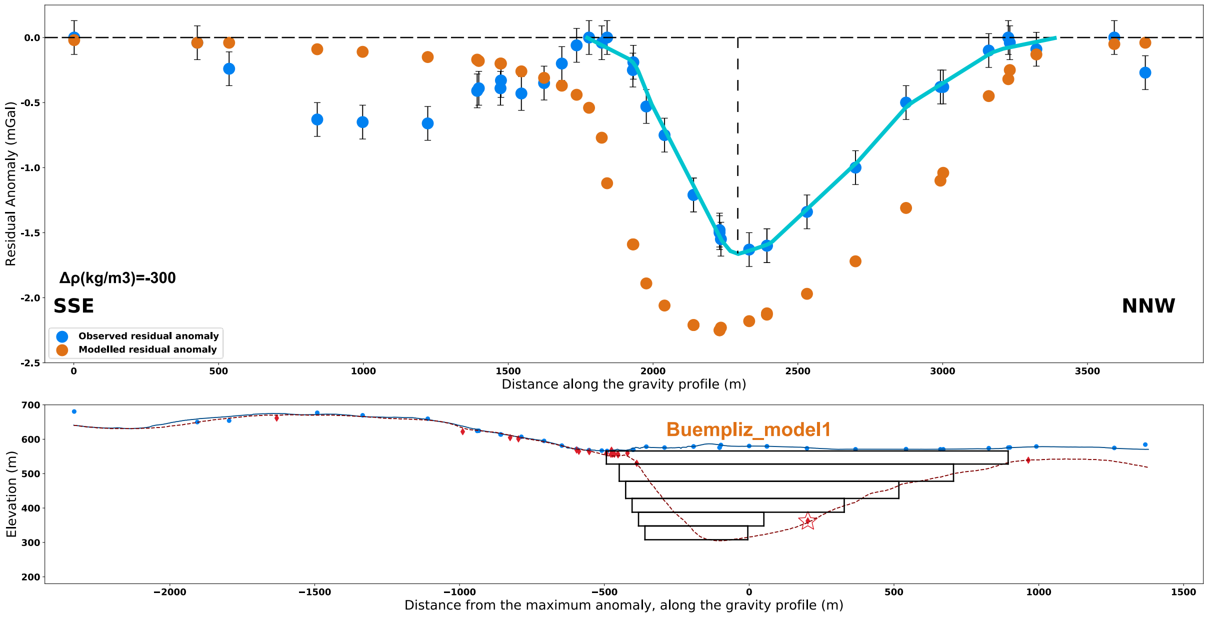

Figure C.7a *Result of the multi-prism model 1 for the Bümpliz profile, where the data has been projected perpendicularly to the gravity profile (projection following the purple line on Figure C6). Top: The blue dots represent the observed residual anomaly, and the orange dots represent the calculated gravity effect in each station for model 1. The black bars indicate our maximum uncertainty of +/- 0.13 mGal (Bandou et al., 2022). Bottom: Elevation and bedrock profile of the Bümpliz profile. The small blue dots document the locations of the gravity stations. The red diamonds indicate drillings that have reached the bedrock and the yellow star shows the position of the deepest drilling from Schwenk et al. (2022). The black rectangles document geometry of the prisms at the intersection with the gravity profile, as shown on figure C.6.*

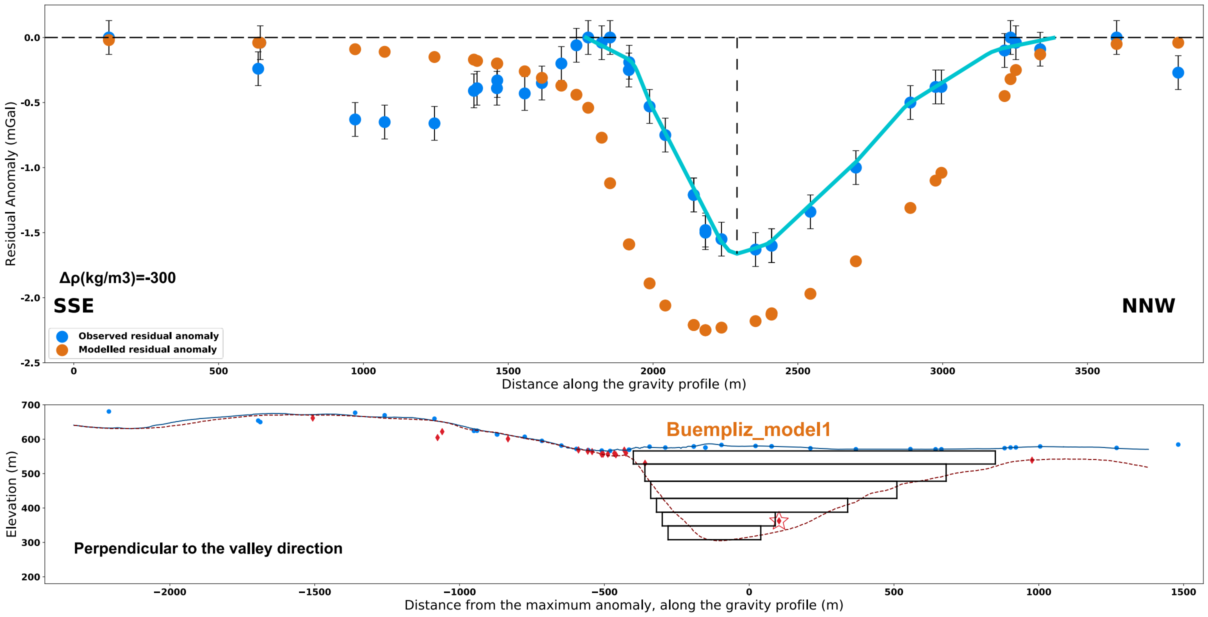

Figure C.7b *Same as Figure C.7a, but exemplified for the case where the projection was accomplished following the green line in Figure C.6a. We used such a projection for the Bümpliz profile where the gravity profile is strongly oblique with respect to the orientation of the long-axis of the overdeepened trough.*

C.5 Back-transformation and projection of the modelling results onto profile

The LCS coordinates of the results of the gravity model calculations (calculated gravity effects, prisms geometries with intersection points) are back-transformed to the Swiss-coordinates following the same steps as in chapter C.2 but in the reverse order and in the opposite sense.

First, we have to flip the z- and x-axes:

$z_{BackLCS2} = z_{LCS} * (-1)$ (C.15),

$x_{BackLCS2} = x_{LCS} * (-1)$ (C.16),

$y_{BackLCS2} = y_{LCS}$ (C.17).

Then we have conduct a back-rotation around the z-axis (compare Figure C.4), but in the opposite sense than before (angle has been multiplied by -1 and thus been flipped in formulae):

$x_{BackLCS1} = x_{BackLCS2} * \cos[\theta * (-1)] + y_{BackLCS2} * \sin[\theta * (-1)]$ (C.18),

$y_{BackLCS1} = -x_{BackLCS2} * \sin[\theta * (-1)] + y_{BackLCS2} * \cos[\theta * (-1)]$ (C.19).

$z_{BackLCS1} = z_{BackLCS2}$ (C.20).

Finally, through a back-translation, we get:

$x_{Back} = x_{BackLCS1} + x_{CHorigin}; y_{Back} = y_{BackLCS1} + y_{CHorigin}; z_{Back} = z_{BackLCS1} + z_{CHmin}$ (C.21).

If all transformations have been accomplished in a correct way, then Swiss *x, y* and *z* coordinates of all stations, locations of drillings, elevation information and coodinates of prisms should be identical with the values after the transformations, i.e.: $x_{CH} = x_{Back}, y_{CH} = y_{Back}, z_{CH} = z_{Back}$.

References

Bandou, D., Schlunegger, F., Kissling, E., Marti, U., Schwenk, M., Schläfli, P., Douillet, G., & Mair, D. (2022). Three-dimensional gravity modelling of a Quaternary overdeepening fill in the Bern area of Switzerland discloses two stages of glacial carving. *Scientific Reports*, 12, 1441. https://doi.org/10.1038/s41598-022-04830-x.

Nagy, D. (1966). The gravitational attraction of a right rectangular prism. *Geophyscis*, 31, 362-271.

Schwenk, M., Schläfli, P., Bandou, D., Gribenski, N., Douillet, G., & Schlunegger, F. (2022). From glacial erosin to basin overfill: a 240 m-thick overdeepening-fill sequence in Bern, Switzerland. *Scientic Drilling,* 30, 17-42. https://doi.org/10.5194/sd-30-17-2022.

Reber, R., & Schlunegger, F. (2016). Unravelling the moisture sources of the Alpine glaciers using tunnel valleys as constraints. *Terra Nova,* 28, 202-211. https://doi.org/10.1111/ter.12211.
