# Supplementary material for: Overdeepenings in the Swiss plateau: U-shaped geometries underlain by inner gorges
Source: Swiss J Geosci. 2023 Dec 5;116(1):19. doi: 10.1186/s00015-023-00447-y (PMC10698152; doi:10.1186/s00015-023-00447-y)
Supplement: Supplementary file 4 — Additional file 4: Appendix D. Profiles that were measured and modelled for this paper, and details on the modelling procedure that we applied to each profile. [file 15_2023_447_MOESM4_ESM.pdf]

Appendix D:
Profiles that were measured and modelled for this paper

D.1 Overview of profiles used for this paper

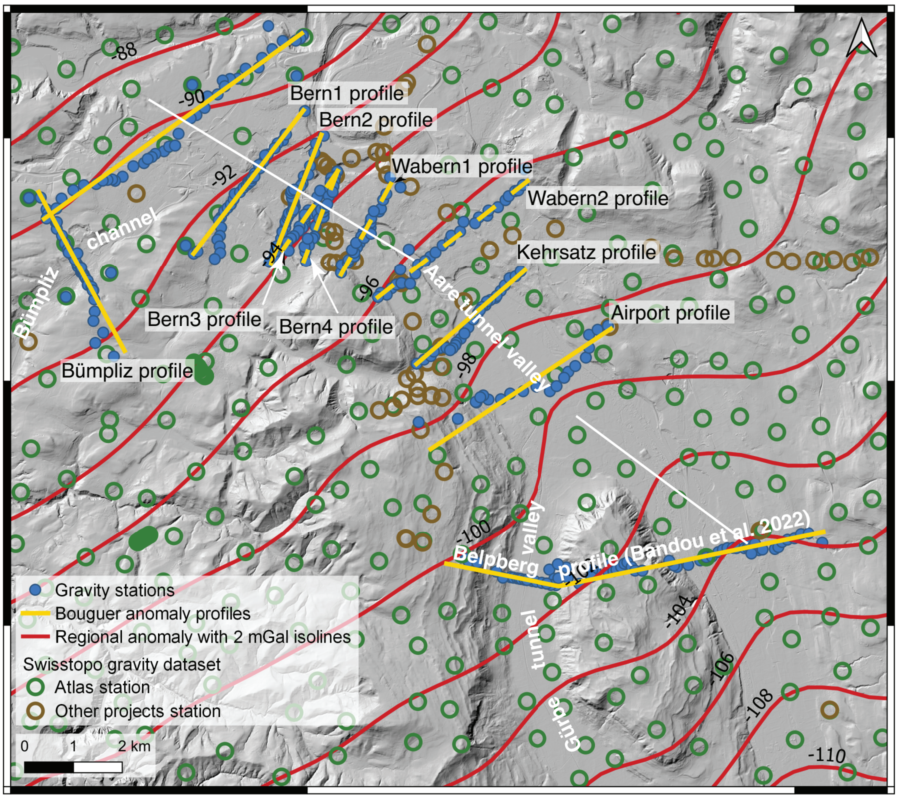

Figure D.1a *Map showing the regional Bouguer gravity map of the study region (extract from the Gravimetric Atlas of Switzerland © swisstopo (Olivier et al., 2008; 2011)) with 2 mGal isolines (solid red lines, with their respective values) and gravity stations (green circles). The brown circles are stations from the swisstopo gravity database which belong to other projects (levelling projects or bases network). The blue dots are the new gravity stations measured during this study and the 7 yellow lines indicate the gravity profiles that were modelled, while the 4 broken yellow lines mark those profiles that weren't modelled. The SwissAlti3D 2m DEM is in the background (© swisstopo).*

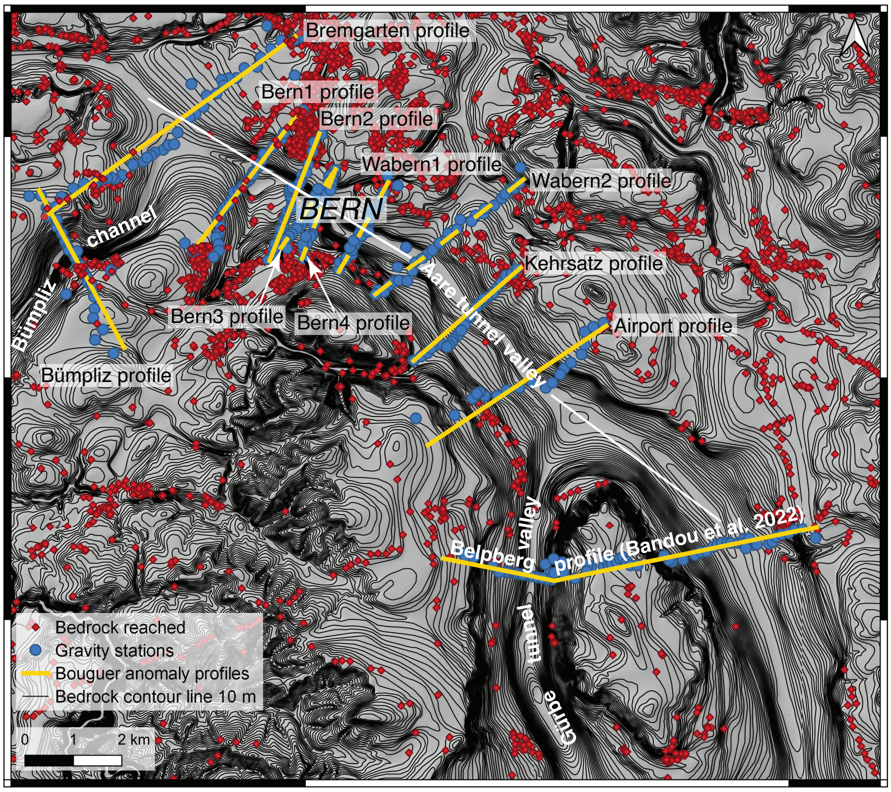

Figure D.1b *Map of the Molasse bedrock topography (Reber and Schlunegger, 2016) including location of drill holes (solid red diamonds) where the bedrock has been reached upon drilling. Superimposed are locations of newly measured gravity stations (solid blue dots), most of them aligned along 7 profiles (solid yellow lines) for primary 2.5D analysis. The Belpberg profile was already analyzed in Bandou et al. (2022). While 6 profiles traverse the main channels of the Aare – Gürbe tunnel valley system, the Bümpliz profile crosses a narrow side channel.*

D.2 The Bümpliz profile

Data collection, estimation of regional gravity gradient, and estimation of residual anomalies

The Bümpliz profile runs SSE – NNW across the proposed bedrock depression according to Reber and Schlunegger (2016, Figure D.1). It closely by-passes the Rehhag drillhole (Schwenk et al. 2022) with a regional Bouguer gravity station near either end of the profile (Figure D.2.1). The profile runs nearly perpendicularly to the Bouguer gravity isolines, thereby crossing the -90 mGal and the -92 mGal isolines. Within the near vicinity and along the profile there exist 5 regional gravity stations that document a local gravity low (Figure D.2.2, green broken line), which suggests the occurrence of a bedrock depression beneath the NNW part of the profile.

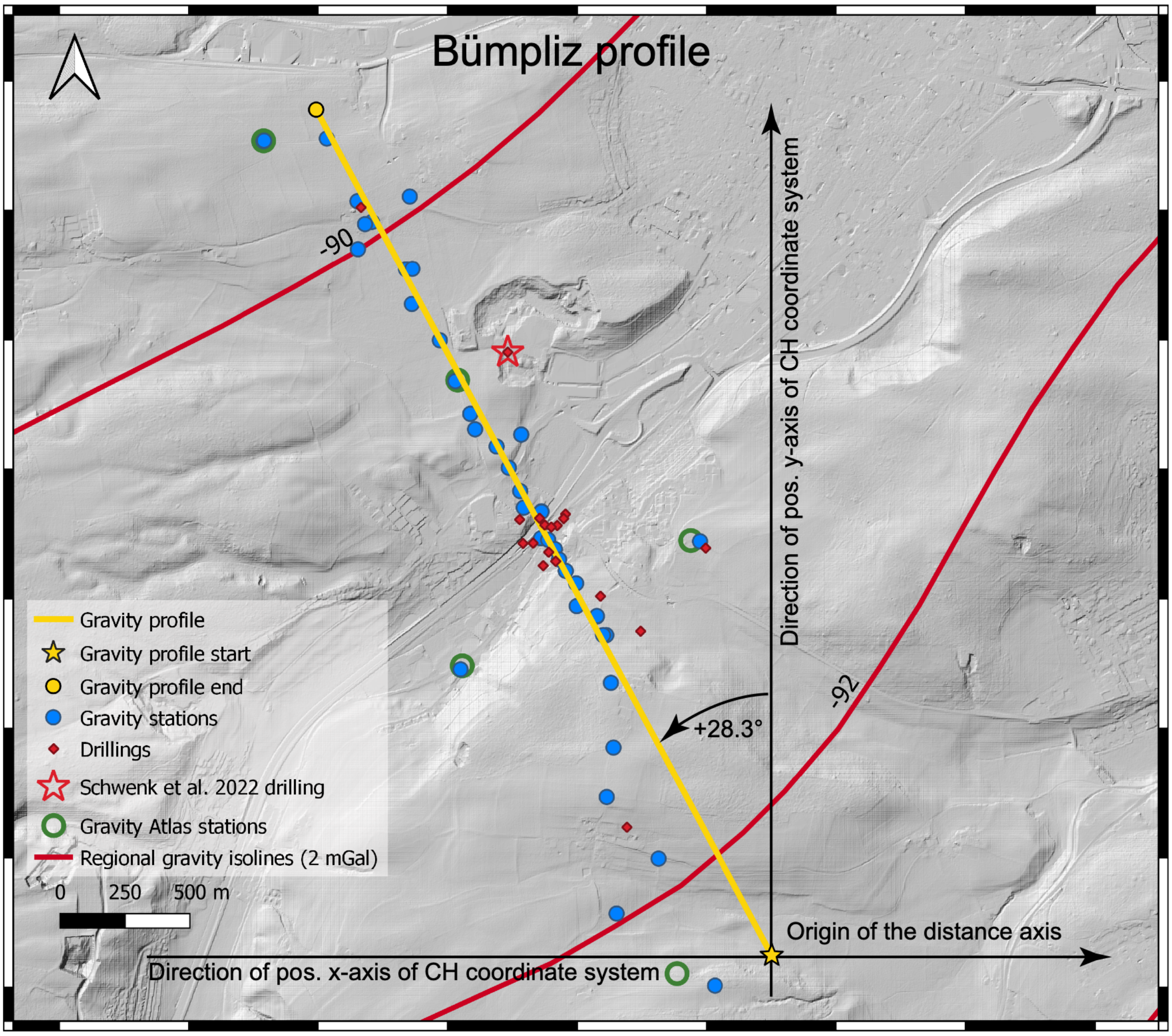

Figure D.2.1 *Enlarged extraction of regional Bouguer gravity map (green circles representing the stations of the Gravimetric Atlas of Switzerland, and the red lines denote the regional gravity isolines), which documents the gravity field in the vicinity of the newly measured Bümpliz gravity profile (yellow line). The SwissAlti3D 2m DEM is shown in the background (© swisstopo). The blue dots are the newly measured gravity stations. The red diamonds show the locations of the drillings that reached the Molasse bedrock. Note that the profile runs almost perpendicularly to the regional gravity isolines with -90 mGal and -92 mGal. This will lead to a clear regional gravity gradient for this profile. This figure also shows the calculated angle between the Swiss coordinates' y-axis (North direction) and the gravity profile.*

Stations that are located more than 500 m distant from the profile (Figure D.2.1) are ignored for the display of the final Bümpliz Bouguer gravity anomaly as the projection distance is too large. The remaining total of 40 gravity stations allow to more precisely estimate the regional gravity gradient along the profile (blue broken line in Figure D.2.2) connecting not only the end points of the profile but

also the margins of the Bümpliz bedrock depression. These are documented by drill hole data that reached the bedrock at shallow levels (Reber and Schlunegger, 2016).

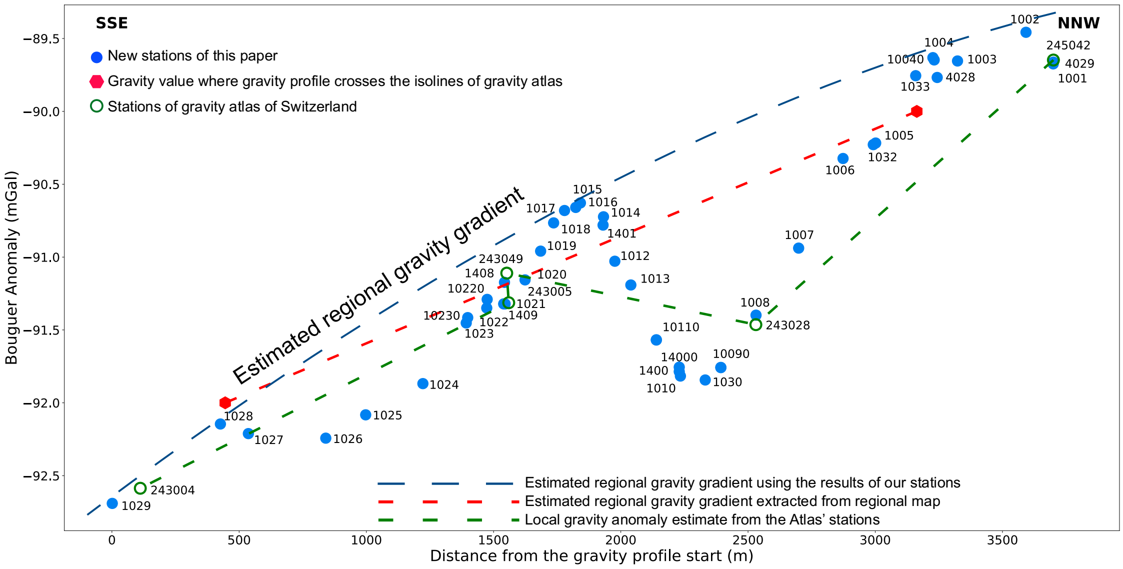

Figure D.2.2 *Bouguer anomaly along the Bümpliz profile. The blue dots indicate the gravity stations measured for this profile (see Appendix A for gravity data) while the green circles are gravity stations from the Gravimetric Atlas of Switzerland (Olivier et al. 2008; 2011). The green broken line denotes the local gravity anomaly along the profile. This is estimated based on the 5 points of the Gravimetric Atlas of Switzerland only. The red broken line denotes the gravity gradient extracted from the regional map (isoline -90 mGal and on NNW profile end -92mGal, see Figure D.2.1). The broken blue line shows the regional gravity gradient along the profile that estimated from all points of the Gravimetric Atlas of Switzerland, which are close to the profile and the newly measured gravity points.*

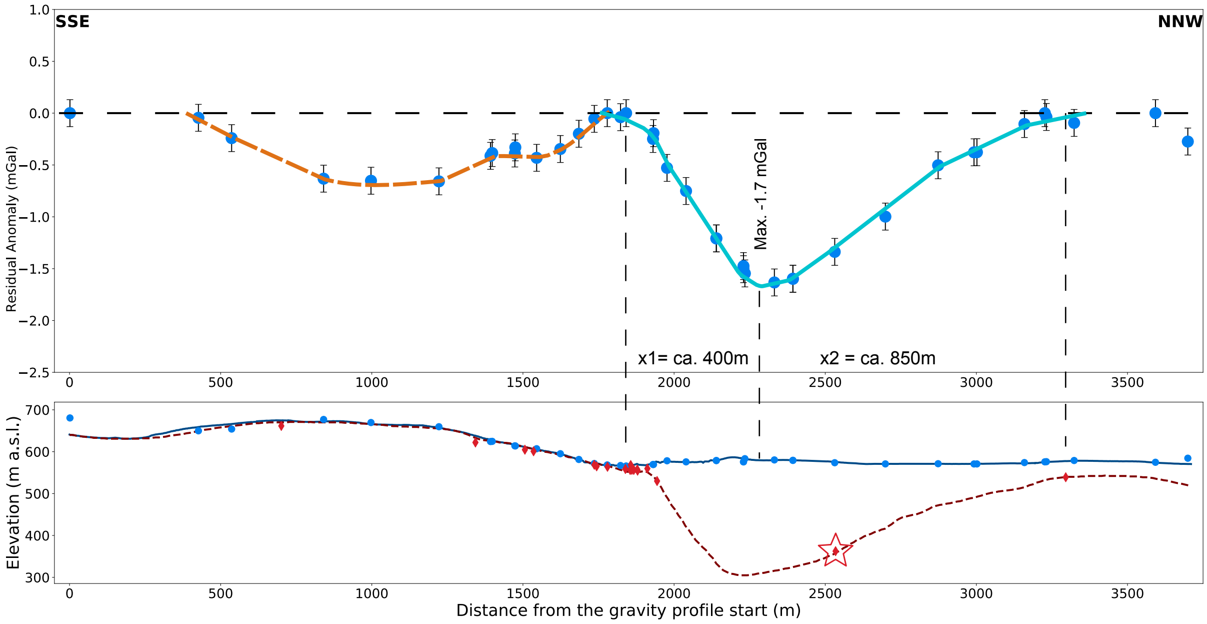

Figure D.2.3 *Residual anomalies and bedrock topography model by Reber and Schlunegger (2016) for the Bümpliz profile. The residual anomaly was calculated by subtracting the regional gravity gradient from the Bouguer anomaly values at each station (blue dots with black error bars of +/- 0.13 mGal). Bottom: Surface topography (SwissAlti3D 2m DEM (© swisstopo)) along the profile (black solid line) and elevation of the gravity stations (solid blue dots). The red broken line illustrates the bedrock topography of the bedrock model of Reber and Schlunegger (2016), and the red star indicates the key drilling information of Schwenk et al. (2002) to constrain the bedrock*

depth of ca. 210m. The main residual anomaly has a V-shaped pattern and reaches a maximum value of -1.7 mGal. It also shows an asymmetry between the two flanks.

Based on the fact that the gravity denotes a potential field, we interpolated the Bouguer gravity values along the profile by a continuous line (dashed light blue line in Figure D.2.2). Upon subtracting the estimated regional gravity gradient from the Bouguer gravity anomaly we obtain the residual gravity anomaly along the Bümpliz profile (Figure D.2.3). This allows us to document a negative local main anomaly with a maximum amplitude of -1.7 mGal. This main anomaly is situated between the profile distances of approximately 1700 m and 3400 m where the values of the estimated regional gravity matche with those of the locally observed gravity. The main anomaly is flanked on the SSE side by a secondary anomaly that most likely reflects the gravity signal of the Molasse bedrock, which has a density of 2500 kg/m^3. This is lower than the standard density of 2650 kg/m^3 used for Bouguer anomaly calculations. Therefore, the positive topography made up of Molasse bedrock appears as a negative residual anomaly in our profile.

<u>Modelling the residual anomalies of the Bümpliz profile</u>

Preparation of the models

The valley direction (the white line, Figure D.2.4) is defined by the general direction of the valley flanks. This allows to define the orientation of the prisms for modelling purposes, which will be parallel to this line. The valley width (white broken line) is defined by using the bedrock model information by Reber and Schlunegger (2016). Here, we used the contour line of 540 m as drillings directly document that the depression starts at this elevation on the NNW flank.

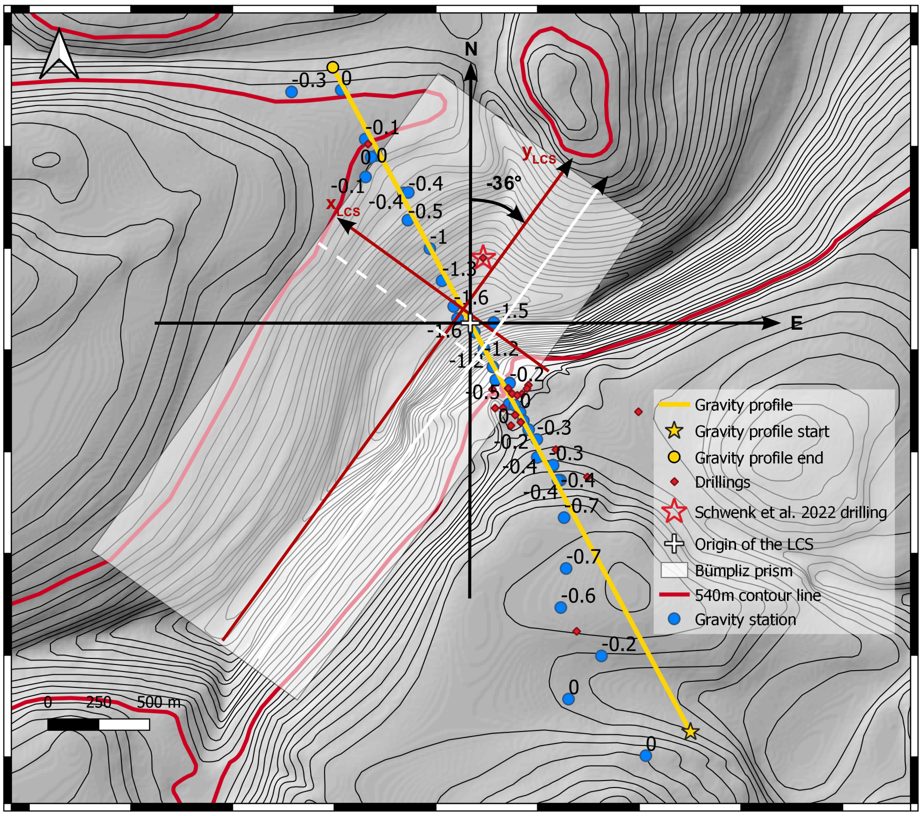

Figure D.2.4 *Map of the bedrock in the region of the Bümpliz profile, using the bedrock model from Reber and Schlunegger (2016). The yellow line indicates the location of the gravity profile, the blue dots show the gravity stations with their residual anomaly values (in mGal) and the red dots are drillings that reached the bedrock. The red x- and y-axes indicate the LCS used for the modelling. The LCS origin, marked by the white cross, denotes the projection of the site onto the profile where the residual anomalies have their maximum amplitude. This location is situated between two gravity stations. Note that the directions of the LCS x-axis and the gravity profile differ significantly. The y-axis is obtained by a clockwise rotation of 36° from the North that denotes the y-axis of the*

Swiss coordinate system. The white rectangle indicates the dimensions of the top prism used to model the overdeepening infill for this profile.

For modelling purposes, however, we defined the width of the top prism using (i) the wavelength of the residual anomaly (Figure D.2.3) and (ii) the distances from the LCS origin for each half width of the prism on the SSW and NNE side, respectively. Therefore, the top prism's width that is based on the residual anomalies and the valley width that we defined from the bedrock model of Reber and Schlunegger (2016) show a slight difference.

Conducting the modelling

For the first model, we employed a model geometry that is characterized by an asymmetry with a much steeper SSE flank compared to the NNW side. Schwenk et al. (2022) inferred density contrasts ranging from ca. -270 kg/m^3 to ca. -420 kg/m^3 between the overdeepening fill and the Molasse bedrock based on the results of measurement accomplished with a Multi Sensor Core Logger (MSCL; Geotek Ltd.). Accordingly, we started with a model where we used a value of -300 kg/m^3 as a first estimate for characterizing the density contrast between the Quaternary deposits and the Molasse bedrock.

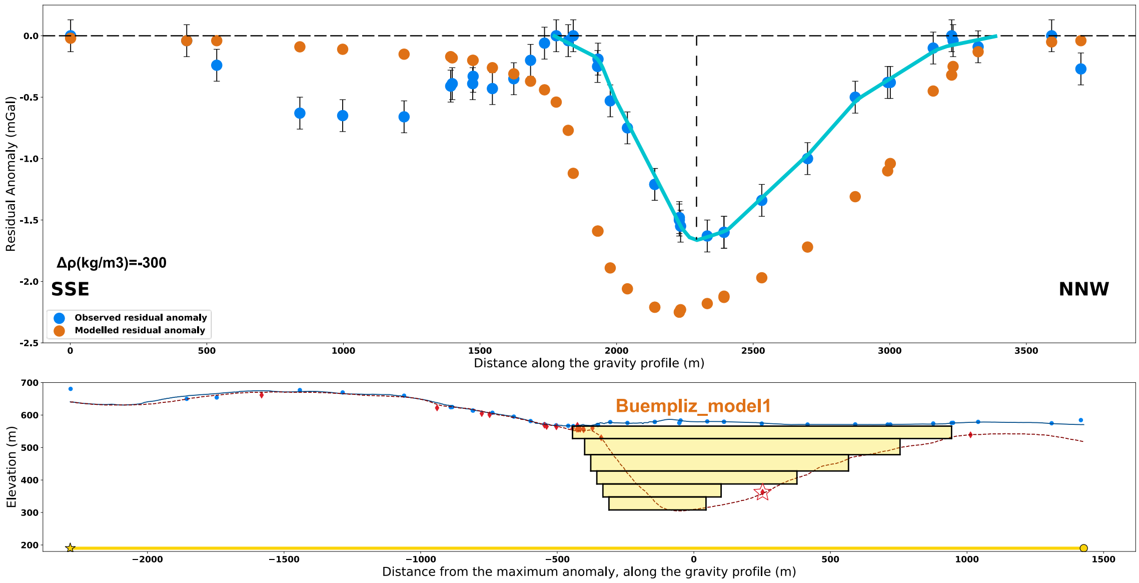

Figure D.2.5 *The figure shows the initial model 1 for the Bümpliz gravity profile, made with 6 prisms with a density contrast of -300 (kg/m^3). Top: The blue dots represent the observed residual anomaly, and the orange dots are the modelled residual anomaly values for model 1. The black bars indicate our maximum uncertainty of +/- 0.13 mGal. Bottom: Elevation profile (SwissAlti3D 2m DEM (© swisstopo)) and bedrock profile (Reber and Schlunegger, 2016) for the Bümpliz section. The blue dots are the gravity stations, the red diamonds indicate drillings that have reached the bedrock. The red star indicates the position of the drilling (Schwenk et al., 2022) in the gravity profile. The black rectangle shows the cross sections of the 5 prisms used. The yellow start is the start of the profile, and the yellow circle denotes the end.*

The results show that while the shape of the anomaly has been well captured, the wavelength of the modelled anomaly is too wide and extends too far towards the flanks in particular on the SSE extremity of the main anomaly (Figure D.2.5). We note that the maximum anomaly is slightly shifted to the SSE, indicating that the deepest section is too wide. Furthermore, the differences between the measured and modelled anomalies are smaller in the very upper part of the trough compared to the deepest section. This indicates that the inferred geometry in the upper part was too wide in comparison to the lower one. The next models 2 to 4 focus on moving towards a more V-shaped geometry (as inferred by the shape of the residual anomaly) by significantly reducing the widths of the prisms on the SSE flank and to a lesser extent on the NNW flank (Figure D.2.6). We additionally adjust the density contrast values, with -500 kg/m^3 for the top prism of the subsequent models, and we increased the density contrast to -350 kg/m^3 for the rest of the prisms. This was done to comply with the average value of

density contrasts between Molasse bedrock and Quaternary sediments measured by Schwenk et al. (2016) and Bandou et al. (2022). We proceeded through modelling the residual anomalies in the upper part, before tackling the deeper levels.

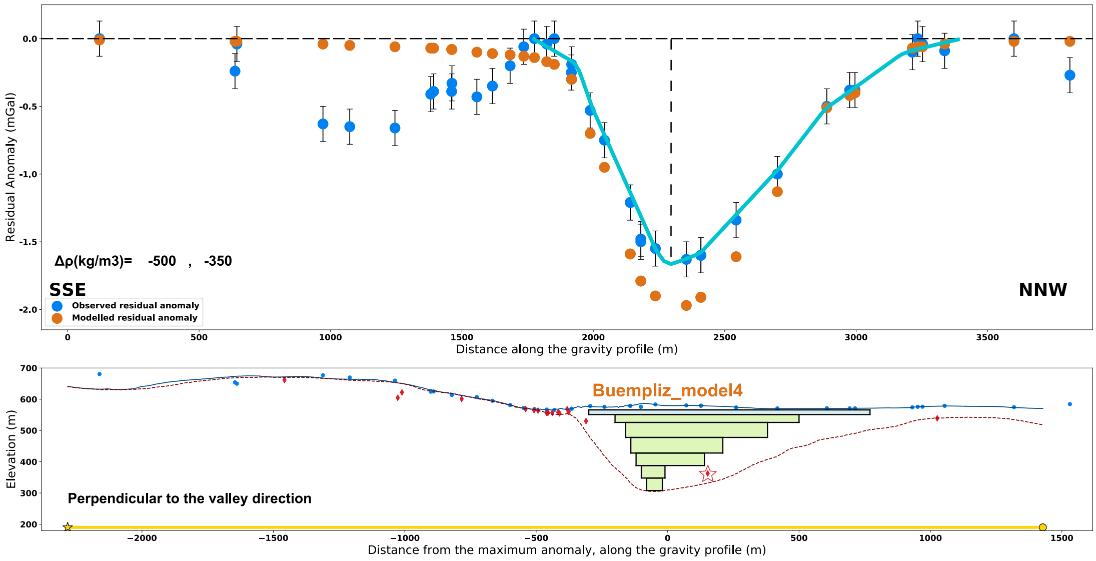

Figure D.2.6 *Plot perpendicularly to the valley direction (LCS y-axis). Model 4 with 7 prisms. Top: The blue dots represent the observed residual anomaly, and the orange dots are the modelled residual anomaly values for model 4. The black bars indicate our maximum uncertainty of +/- 0.13 mGal. Bottom: Elevation profile (SwissAlti3D 2m DEM (© swisstopo)) and bedrock profile (Reber and Schlunegger, 2016) the Bümpliz section. The blue dots are the gravity stations, the red diamonds indicate drillings that have reached the bedrock. The red star indicates the position of the drilling (Schwenk et al., 2022) in the gravity profile. The black rectangle shows the cross sections of the 7 prisms used. The light blue colour indicates the prism with a density contrast of -500 kg/m^3 while the light green one shows prisms with a density contrast of -350 kg/m^3.*

In order to further improve the fit between the model results and the residual anomalies for the lower half geometry, we proceeded by splitting the prisms towards the maximum depth. This also allowed us to adjust the geometry on the NNW flank during modelling steps 5 through 8. This then resulted in the final model illustrated in Figure D.2.7 where the modelled and measured residual anomalies perfectly fit within the uncertainties. For a discussion of the misfit between the bedrock topography model of Reber and Schlunegger (2016), the depths of some drillings and our results, the reader is referred to the main text.

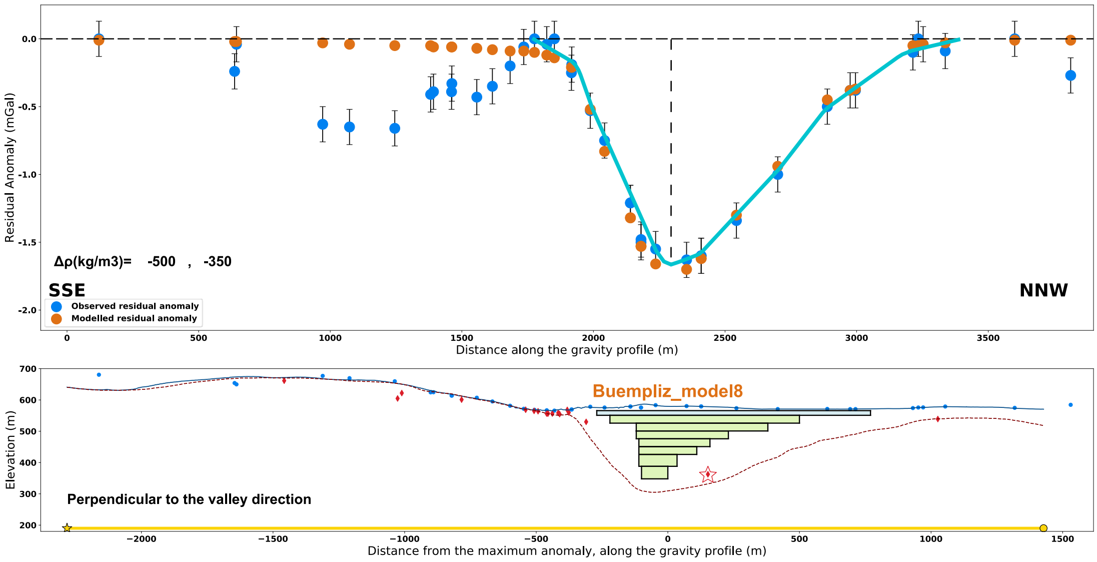

Figure D.2.7 *Plot perpendicular to the valley direction (LCS y-axis). Final model, model 8 with 8 prisms. For explanations of symbols see legend to Figure D.2.6.*

D.3 The Bremgarten profile

We placed the Bremgarten profile in the NW corner of the study region (Figure D.1) first to retrieve more information on the downstream end of the Aare tunnel valley and second to connect outcropping Molasse bedrock on either side of the main overdeepening where the occurrence of shallow bedrock locations was documented by drillhole information.

The 46 newly measured gravity stations along the Bremgarten profile complement the 5 stations of the regional Bouguer map (Olivier et al., 2008, 2010) (Figure D.3.1). While the previously known and the newly measured Bouguer values at nearby stations correspond very well, the Bremgarten gravity profile allows us to assess the local Bouguer gravity field in unprecedented detail. The existence of a bedrock depression beneath the Bremgarten profile is already suggested by the five stations of the regional gravity map (green points and broken green line in Figure D.3.2) though only the new gravity points allow us to assess the details of the trough's geometry. Unsurprisingly, the newly measured gravity stations document the need to update the isolines of the regional Bouguer gravity map (see red dots connected by broken red line in Figure D.3.2).

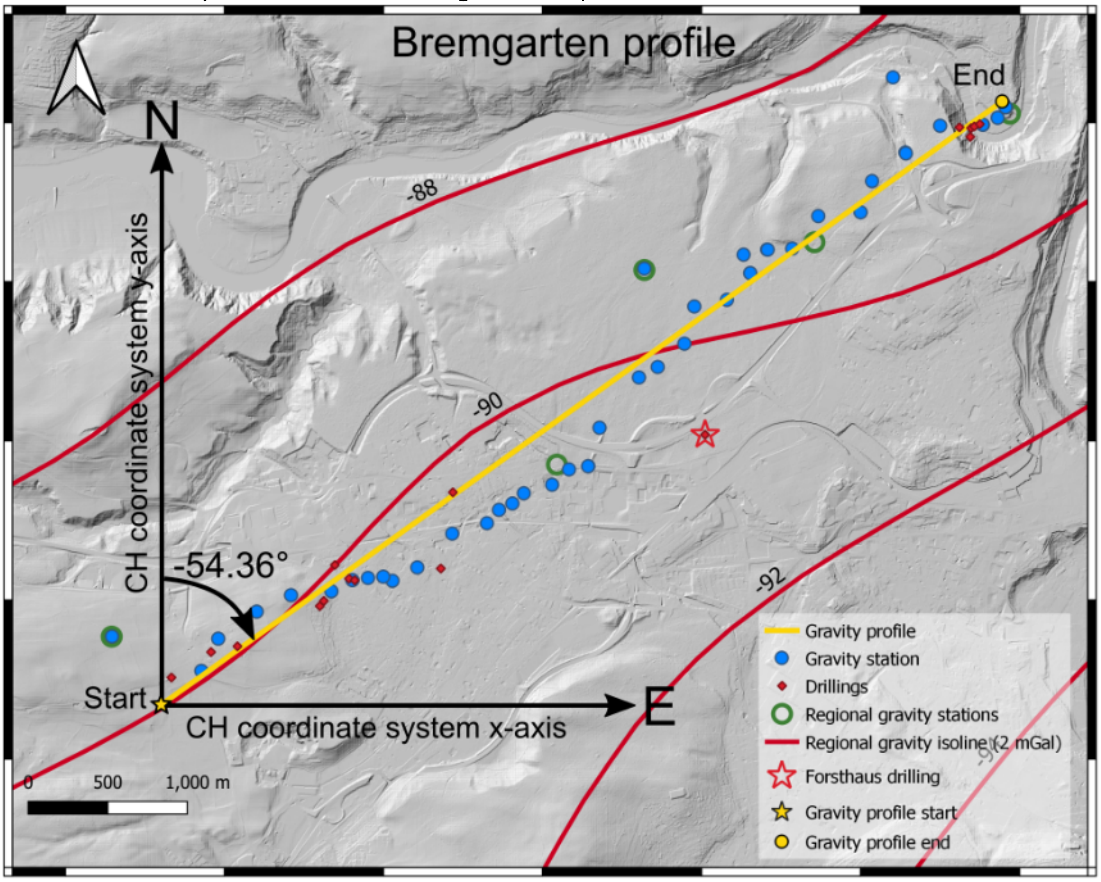

Figure D.3.1. *Details of the regional Bouguer gravity map (green circles representing the gravity stations and red lines denote the regional gravity isolines) documenting the gravity field in the vicinity of the Bremgarten gravity profile (yellow line).* The SwissAlti3D 2m DEM is in the background (© swisstopo). *The blue dots are the newly measured gravity stations. The red diamonds show the locations of the drillings that reached Molasse bedrock. Note the profile runs nearly parallel to the regional gravity isoline -90 mGal except near the NE end of the profile, where we may estimate a regional gravity value of -89 mGal.*

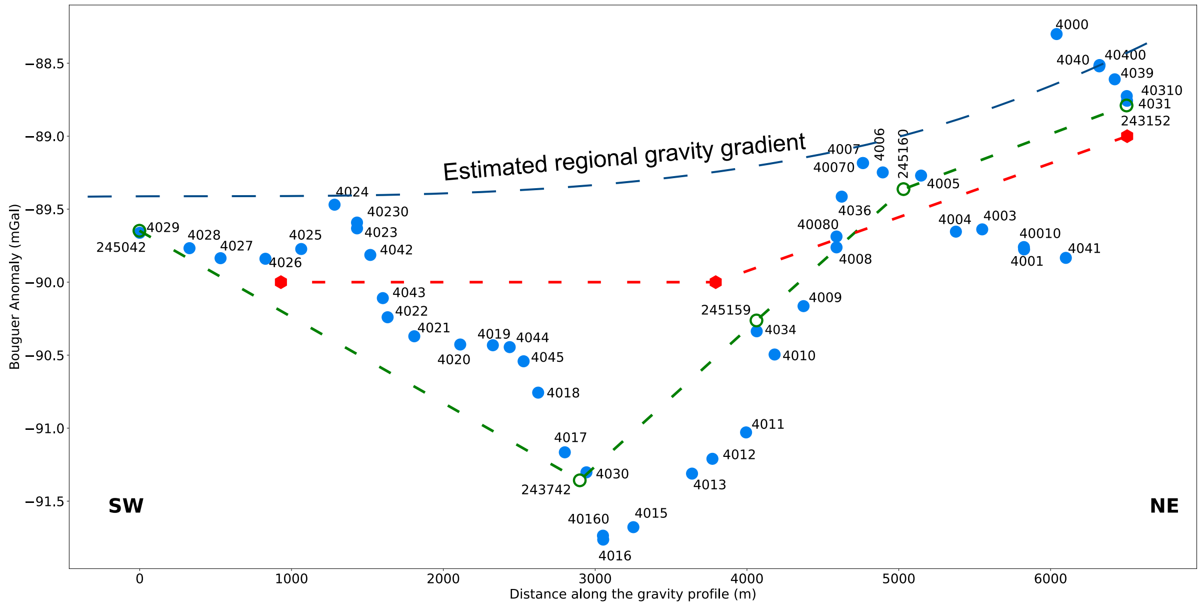

Figure D.3.2 *Bouguer anomaly along the Bremgarten profile with newly measured gravity stations (MP, blue solid dots). The green circles are gravity stations from the Gravimetric Atlas of Switzerland (Olivier et al. 2008; 2011). The green broken line denotes the local gravity anomaly along the profile as estimated using the 5 points of the Gravimetric Atlas of Switzerland only. The red broken line denotes the gravity gradient extracted from the regional map (isoline -90 mGal and on NE profile end -89 mGal, see Fig. 3). The broken blue line shows the regional gravity gradient along the profile estimated from all gravimetric atlas points in vicinity of the profile and the newly measured gravity points. Based on this regional gradient we estimate a maximum amplitude of the residual gravity anomaly of about 2.5 mGal at the MP 4016.*

Based on surface geology and drill hole information, the bedrock in the vicinity of our gravity profiles in the Bern region is rather uniformly made up of consolidated Molasse sediments with a bulk rock density of 2500 kg/m^3 (Bandou et al. 2022). Quaternary sediments in the region though are known to be of significantly lower densities. Consequently, we may expect various local anomalies with reduced gravity values while local anomalies with an increased gravity (albeit with a short wavelength) will be lacking. Using these as boundary conditions as constraints, we estimate the regional gravity gradient as illustrated by the broken blue lines in Figure D.3.2. Finally, we obtain the residual anomaly for the Bremgarten profile (Figure D.3.3) by calculating the difference between the estimated gravity gradient and the Bouguer anomaly values. Again, we observe two short-wavelength and low-amplitude local anomalies on either side of the main residual gravity anomaly. This main anomaly has a maximum amplitude of ca. -2.5 mGal that is caused by the sedimentary fill of the tunnel valley. This main residual gravity anomaly points towards a strong asymmetry between the NE and SW flanks of the tunnel valley, with the later also having a more complex geometry. The base of the overdeepening seems to be narrow and V-shaped, and the flank appears to be steeper on the SW side than on the NE. Further up, the through widens, and both flanks appear to have a similar slope. Finally, towards the top, the SW part of the residual anomaly describes a plateau, which is the base of a much wider upper segment with a depth that is constrained by a drilling. We will use this depth level as a key information for the modelling. On the other side, the NE flank seems to have a more uniform slope until the near surface. Figure D.3.3 thus clearly defines the wavelength of the tunnel valley's residual anomaly along the profile, which is approximately 3.85 km. Note that the residual anomaly (shown by the solid light blue line in Figure D.3.3) reaches the predefined regional gravity gradient along the profile (zero residual gravity effect) shortly beyond the location where the bedrock depth becomes negligible on either valley side.

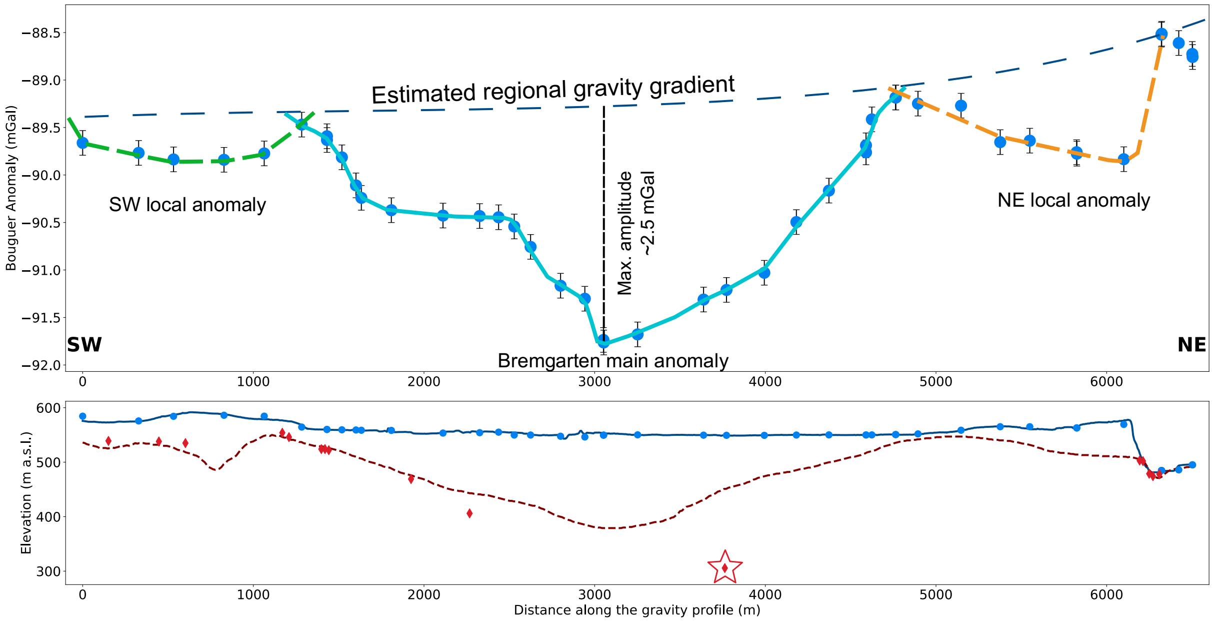

Figure D.3.3 *Residual anomalies and bedrock topography model by Reber & Schlunegger (2016) for the Bremgarten profile. By subtraction of the estimated regional gradient from the Bouguer gravity, we obtain an asymmetrically V-shaped anomaly between 1150 m and 5000 m profile distance with a maximum amplitude of -2.5 mGal at ca. 3050 m. This residual anomaly is located in the center part, and it is bordered by two shorter wavelength anomalies on either side caused by a local low-density topography and near-surface bodies (broken orange lines). The blue dots represent the residual anomaly calculated for the stations; the black bars represent the estimated uncertainty of the newly measured gravity values of +/- 0.13 mGal. The residual anomaly not only documents a strong asymmetry between its two flanks, but it also indicates a difference between the lowest part, more V-shaped, and the upper part, more U-shaped, of the overdeepening. Indeed, the SW flank not only shows a stronger gravity gradient, indicating a steeper flank, but also presents a plateau, absent in the NE flank. The red stars indicate locations of drillings with bedrock information providing important constraints for the subsequent gravity modelling. The light blue line shows the anomaly caused by the overdeepening.*

<u>Modelling the residual anomalies of the Bremgarten profile</u>

We placed the prims for modelling using the same strategy as for the Bern1 profile (see next section). In order to establish an initial multi-prism model for the Bremgarten profile, we rely on the drilling constraints (Reber and Schlunegger 2016) and the shape of the residual anomaly (Figure D.3.3). The latter allows us to determine the steepness of a flank, and it offers constraints to infer if the cross-section geometry is asymmetric and characterized by plateaus. The aim of the first models was to define a general geometry for the bedrock, where the resulting gravity effect fits the main features of the observed residual anomaly. In model 1, we employed a uniform density contrast of -300 kg/m3 for the sedimentary volume. Yet this model returned a model geometry where the general wavelength of the residual anomaly is decently well reproduced, but where the maximum amplitude doesn't match our observed value. In addition, the resulting effect of such an overdeepening fill was overestimated on the SW flank. A reduction of the widths of the lowermost prisms would reduce the maximum anomaly even more, which then confirmed that a density contrast of -300 kg/m^3 is too small. In model 2, we tested the effect where a density contrast of -500 kg/m^3, which proved to be adequate for modelling Gürbe-Aare situation (Bandou et al., 2022). In a third model, we considered as scenario where the where the uppermost two prisms have a higher density contrast (-500 kg/m^3) than the lowermost prisms, where a density contrast of -350 kg/m^3. The modelling results indicate that the modelled geometry is still too wide on the NE flank. This also concerns the situation at greater depths on the SW flank. Also on the SW flanks but on its shallow part, the third prism appears to be too short, while the second prism is still slightly too wide (Figure D.3.4).

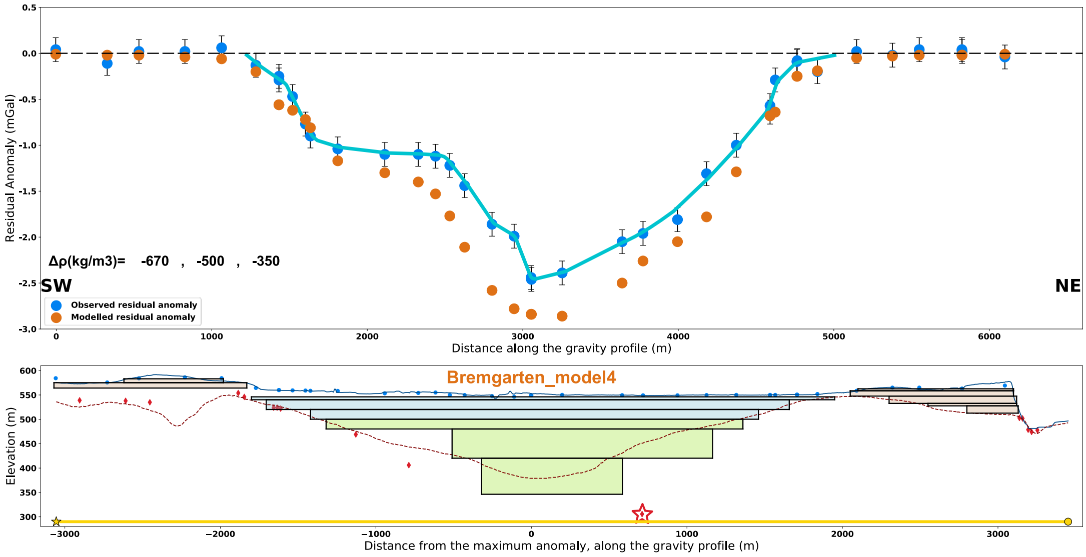

Figure D.3.4 *Observed residual anomaly and calculated gravity effect for model 4 for the Bremgarten section. Top: The blue dots represent the observed residual anomaly, and the orange dots represent the calculated gravity effect at each station for model 4. The black bars indicate our maximum uncertainty of +/- 0.13 mGal. Bottom: Elevation profile (SwissAlti3D 2m DEM (© swisstopo)) and bedrock profile (Reber and Schlunegger, 2016) of the Bremgarten profile. The small blue dots document the locations of the gravity stations. The red diamonds indicate drillings that have reached the bedrock, and the red star is the Forsthaus drilling. The black rectangles document the cross sections of the 6 prisms used for modelling. The top three prisms have a density contrast of -500 kg/m^3 (light blue) while the remaining bottom three have one of -350 kg/m^3 (light green). Modelling conclusions: The general shape of the observed anomaly is now rather well reproduced by the model. The match between model results and observation is good for the upper part but still requires adjustments as the gravity effect is still slightly overestimated. The lower part of the trough is still too wide and too U-shaped.*

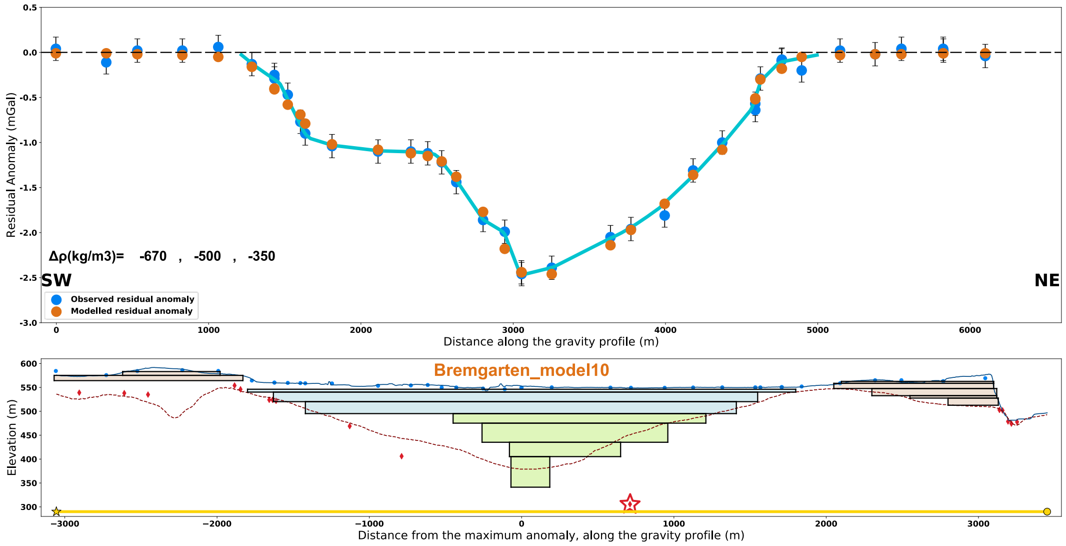

Figure D.3.5 *Observed residual anomaly and calculated gravity effect for model 10 for the Bremgarten section. Top: The blue dots represent the observed residual anomaly, and the orange dots represent the calculated gravity effect at each station for model 10. The black bars indicate our maximum uncertainty of +/- 0.13 mGal. Bottom: Elevation profile (SwissAlti3D 2m DEM (© swisstopo)) and bedrock profile (Reber and Schlunegger, 2016) of the Bremgarten profile. The small blue dots document the locations of the gravity stations. The red diamonds indicate drillings that have reached the bedrock, and the red star is the Forsthaus drilling. The black rectangles document the cross sections of the 7 prisms used in this work. The top three prisms have a density contrast of -500 kg/m^3, while the remaining four prisms at the bottom have one of -350 kg/m^3. Modelling conclusions: The modelled gravity signals now perfectly fit with the observed ones at almost all stations.*

This leads us then to model 5 to 10, where we split the prisms to better adjust the prism's geometry for the upper part of the trough and where we shortened the prisms at greater depths to reduce the modelled wavelength and to better reproduce the plateau. We also adjusted the prism's geometry at greater depth to get a better fit between the model results and the observations.
The final model 10 (Figure D.3.5) discloses a complex geometry for the Bremgarten profile. The overdeepening's cross-section shows a strong asymmetry between the two flanks. The NE flank is wide and U-shaped, while the SW flank has multiple steps and apparently a large plateau in the shallower part of the trough. The geometry documents at least three main sections, a wide U-shaped upper section and a narrower and steeper middle section especially on the SW flank. In addition, the lowermost part is narrow and V-shaped.

D.4 The Bern1 profile

<u>Data collection and estimation of regional gravity gradient, and estimation of residual anomalies</u>

The Bern1 profile runs SW-NE within the city of Bern and crosses perpendicularly the wide overdeepened valley located beneath it (Figure D.1). It starts in the SW between the -92 mGal and the -94 mGal isolines on a mountain ridge referred to as the Könizberg. It runs obliquely to the direction of the gravity isolines and crosses the -92 mGal isoline after c. 2 km, and it finally ends in the NE between and -92 mGal and the -92 mGal isolines within the city of Bern (Figure D.4.1). The bedrock model from Reber and Schlunegger (2016) shows that the overdeepening has a side channel in the South, located next to the start of the gravity profile, and it suggests that the trough has a rather triangular shape (Figure D.1).

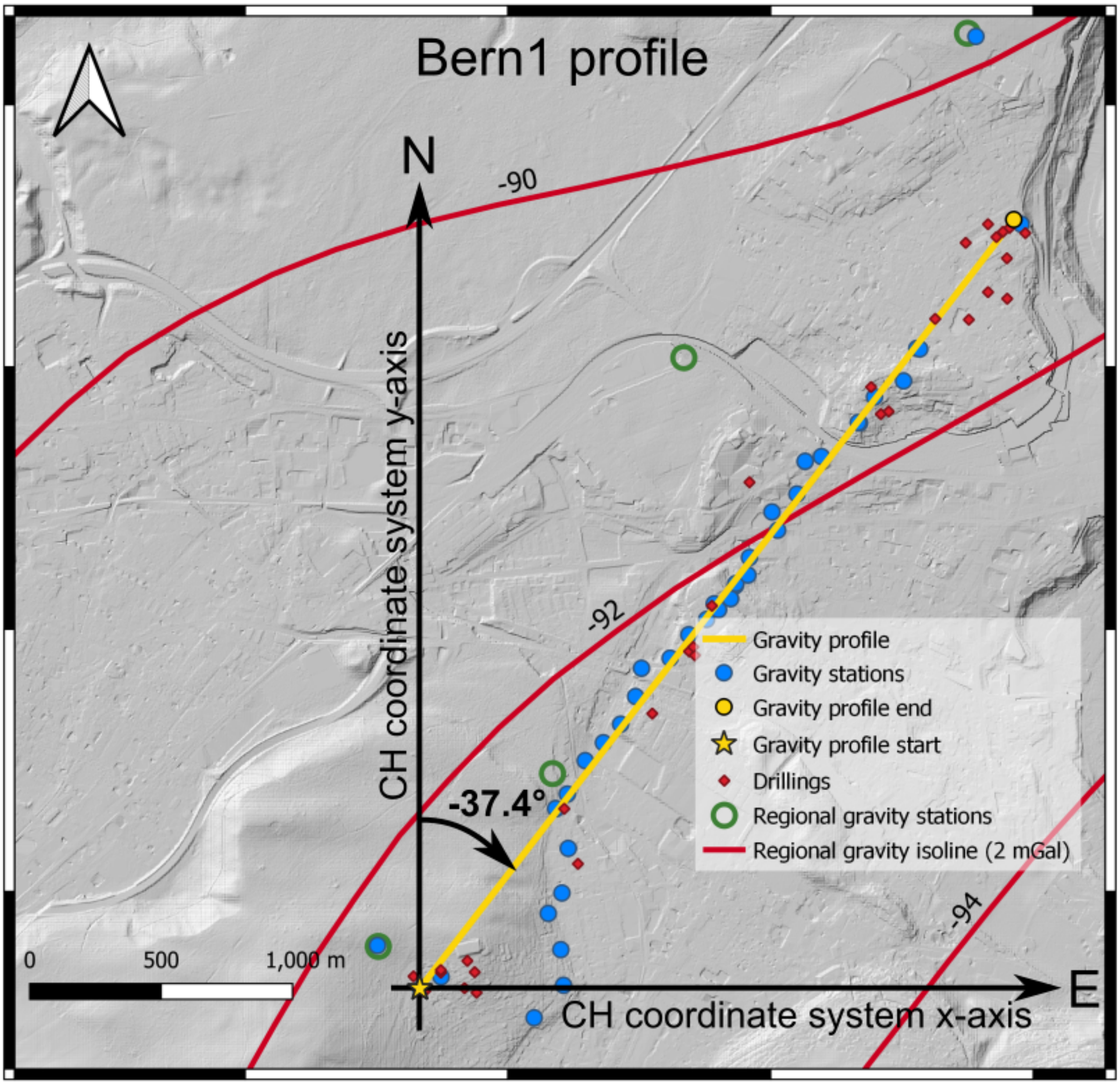

Figure D.4.1 *This map shows the SwissAlti3D 2m DEM (© swisstopo) of the area of the Bern1 profile. The yellow line denotes the gravity profile in the direction rotated 37.4 degrees clockwise relative to the North. The yellow star and dot indicate the start and the end of the profile, respectively. The coordinates of the profile start are (2'597'665.3, 1'197'629.9) in the Swiss coordinate system LV95. The blue dots are the newly measured gravity stations, the green circles are the stations of the Gravimetric Atlas of Switzerland; the red lines show the regional gravity isolines interpolated from the atlas' stations with an interval of 2 mGal (Olivier et al. 2008; 2011). The red dots indicate the location of drillings that have reached the bedrock. All the drillings within 150 m distance to the profile are shown. The black axes indicate the directions of the Swiss coordinate system.*

A total of 41 stations were measured to assess the Bouguer gravity anomaly along the Bern1 profile. We estimated the regional gravity gradient using MP 1126 on the NE profile end, which is a point that is well constrained by drillings (Figure D.4.2), and MP 1127, which is a station far from the profile, but which helps integrating our stations into the regional context. We also used the green station 242741, situated at c. 2700 m distance from the profile start, as constraint. On the SW flank, we used MPs 1202 and 1201 as they are located at the foot of the Könizberg, a mountain ridge made up of Molasse bedrock, and measured additional points on top of the Könizberg. Among these stations MP 1129 is close to the regional gravity stations 245026 (Figure D.4.2). These stations were thus also used to constrain the regional gravity gradient. We observe that the coverage of stations from the atlas of Switzerland that was used to calculate the regional gravity isolines (Olivier et al., 2008, 2011) does not disclose the occurrence of an overdeepening. However, because the bedrock topography map of Reber and Schlunegger (2016) does suggest the occurrence of a major bedrock depression in this region (Figure D.1), we saw the need for collecting further data to increase the coverage of gravity stations.

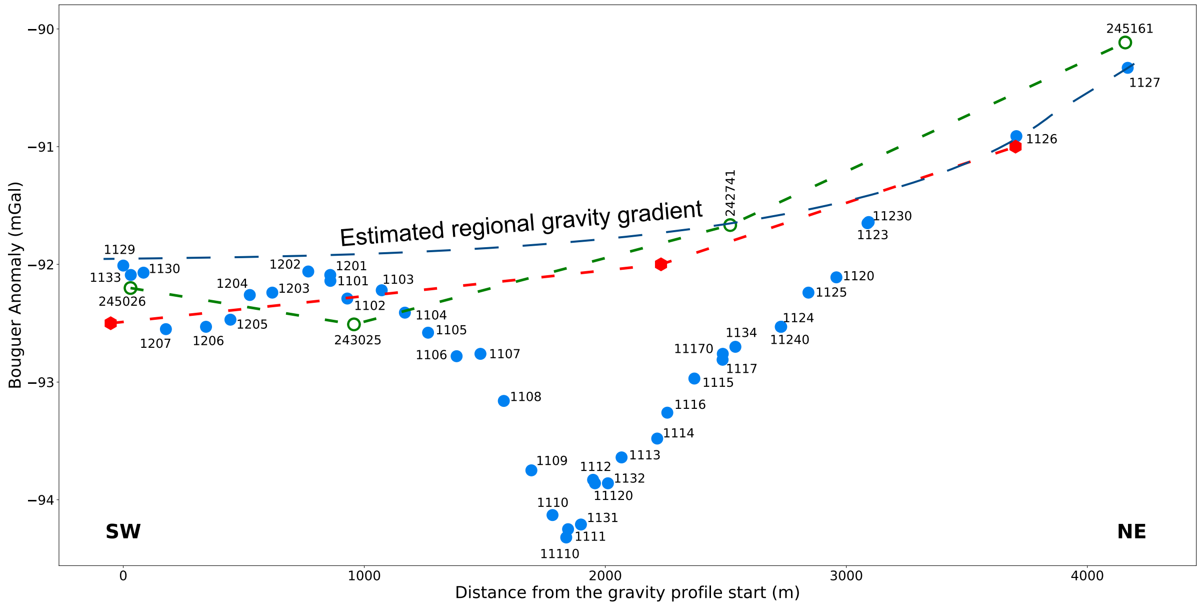

Figure D.4.2 *Bouguer anomaly plot of the Bern1 profile. The blue dots represent the 41 newly measured gravity stations. The green circles denote the gravity stations from the Gravimetric Atlas of Switzerland (Olivier et al. 2008; 2011) that were used to calculate the regional gravity field (Figure D.4.1). Based on this regional gravity map one may estimate the broad tendency of the regional gravity gradient along the Bern1 profile. The red broken line represents a linear interpolation around the -92 mGal isoline (Figure D.4.1) and the green broken line denotes a linear interpolation between the few nearby gravity stations of the atlas. In general accordance with these first-order estimates we define the best estimate of the regional gravity gradient (blue broken line) on the basis of the newly measured stations in combination with the data of the Gravimetric Atlas of Switzerland.*

For the final plot of the Bouguer anomaly (Figure D.4.3), station MP 1127 was removed as it is located too far off the profile, reducing the number of stations used for further processing to 40. The local anomaly of about 700 m wavelength and -0.5 mGal amplitude identified on the SW end of the profile is likely caused by the effect related to the density of the Molasse bedrock underlying the Könizberg. Because the Molasse bedrock has a lower density (2500 kg/m^3) than the one used for the topography correction in standard Bouguer anomaly calculations (2670 kg/m^3), such mountain structures will result in a negative residual anomaly signal. In addition, the Quaternary fill of the side channel farther south could also contribute to this negative residual anomaly signal (Figure D.1).

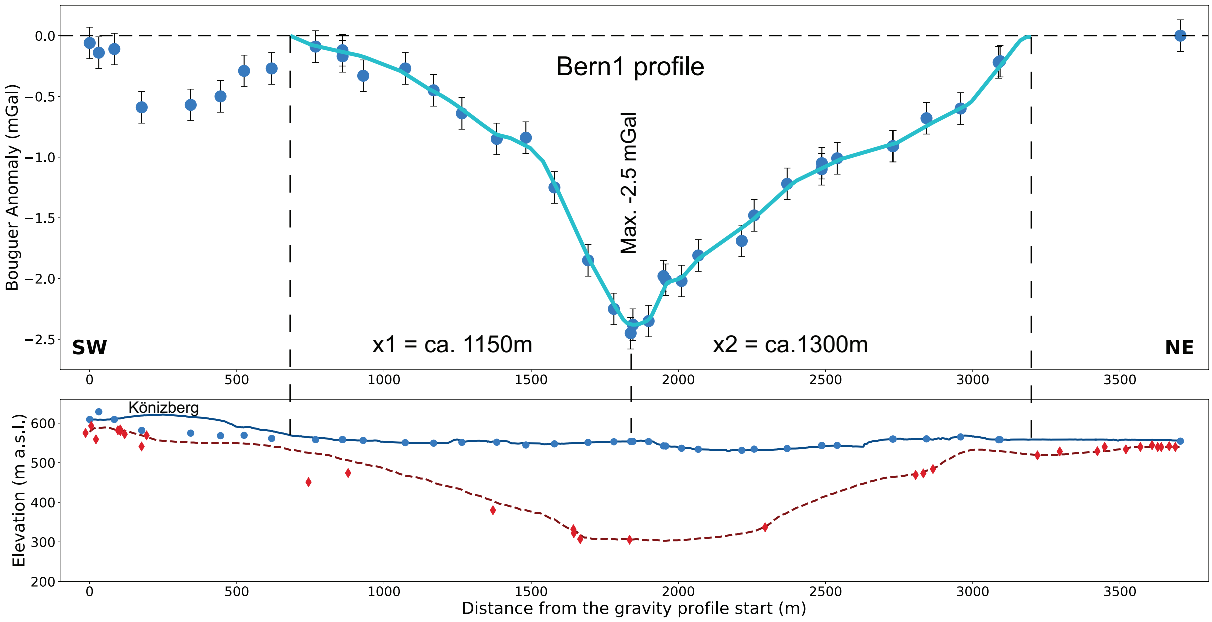

Figure D.4.3 *Residual anomaly for the Bern1 profile. The residual anomaly was calculated by subtracting the regional gravity gradient from the Bouguer anomaly values at each station (blue dots with black error bars of +/- 0.13 mGal) (see Figure D.4.2). The light blue line highlights the main anomaly of the Bern1 profile. Bottom: Surface topography (SwissAlti3D 2m DEM (© swisstopo)) along the profile (blue solid line) and elevation of the gravity stations (solid blue dots). The red broken line illustrates the bedrock topography of the model Reber and Schlunegger (2016). The red diamonds indicate the position of the drillings that reached the bedrock. The drilling located directly beneath the location where we determined the maximum amplitude of the residual gravity anomaly provides information about the maximum depth. The V-shaped residual anomaly of -2.5 mGal (maximum amplitude) and a wavelength of nearly 2500 m shows a slight asymmetry between the two flanks and a wide top with a narrow deeper part of the overdeepened valley.*

Modelling the residual anomalies of the Bern1 profile

Preparation of the models

The white line with the black arrowhead (Figure D.4.4) denotes the general tunnel valley‘s direction, defined to be parallel to the flanks of the overdeepening as proposed by the bedrock model of Reber and Schlunegger (2016). This line will define the y-axis of the LCS y-axis (see Figure D.4.4), which is oriented parallel to the flanks of the top prism. The white dashed line indicates the valley width, which is constrained by the bedrock topography model of Reber and Schlunegger (2016) and set here to the 530 m contour line. Please note that the location and width of the top prim used for modelling deviates from the white dashed line that delineates the valley width based on the 530m contour line. This is explained by the pattern of the residual anomaly, which indicates that the geometry of an overdeepening can deviate from the bedrock topography map of Reber and Schlunegger (2016).

Based on the bedrock model information by Reber and Schlunegger (2016), we estimate a prism length of 2 km to the NW and 1.5km to the SE. A further increase in the length leads to a negligible augmentation of the gravity signal along our profile (Bandou et al., 2022).

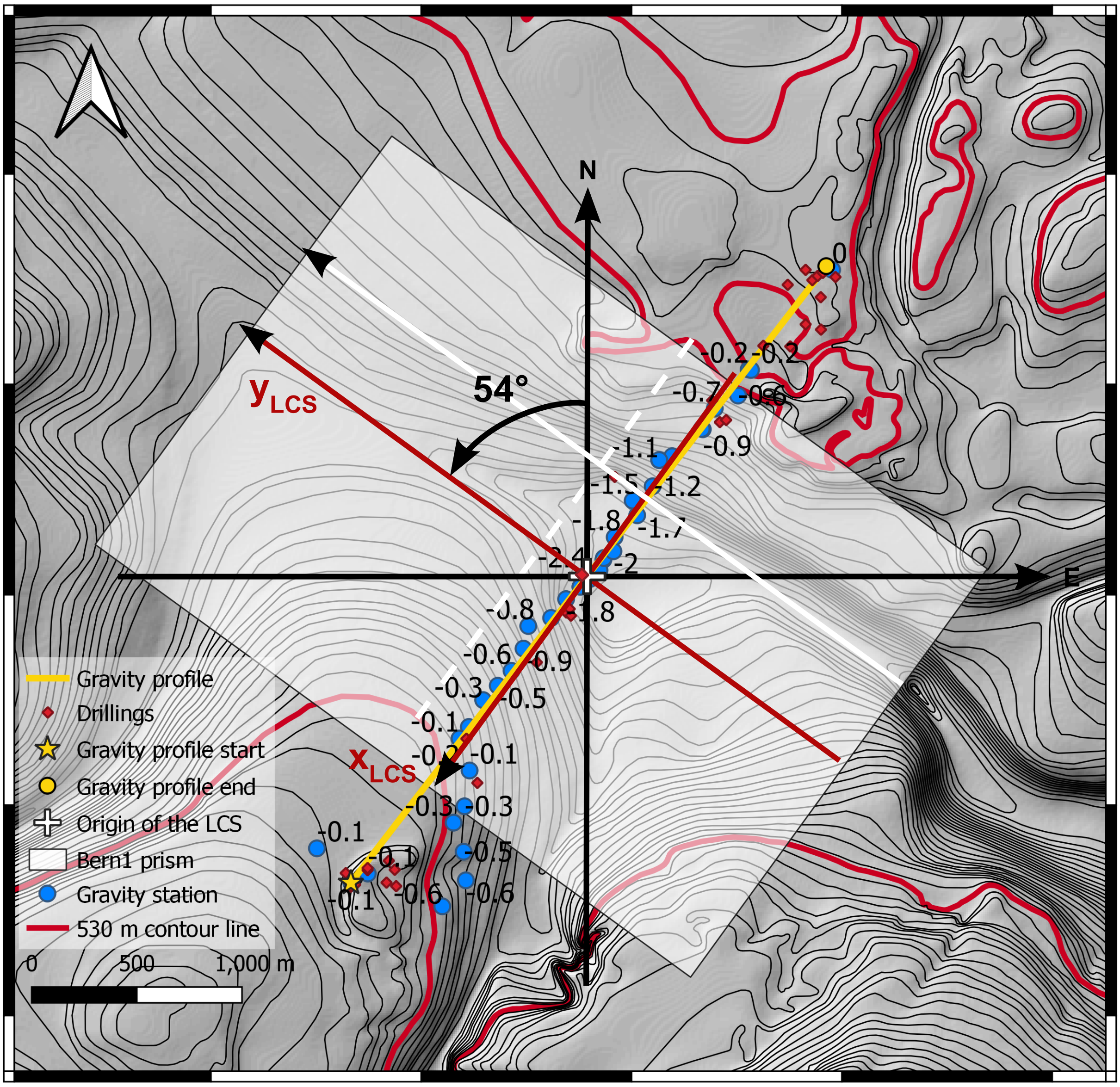

Figure D.4.4 *Map of the bedrock in the region of the Bern1 profile, using the bedrock model from Reber and Schlunegger (2016). The yellow line indicates the location of the gravity profile, the blue dots show the gravity stations with their residual anomaly values and the red dots are drillings that reached the bedrock. The red x- and y-axes indicate the LCS used for the modelling. The LCS y-axis is oriented parallel to the tunnel valley main direction, indicated by the white line, and the direction of the LCS x-axis (red arrow) parallels the white broken line and is oriented perpendicular to the direction of the overdeepening. Both axes are crossing the LCS origin, marked by the white cross. This location denotes the projection of the gravity station with the maximum amplitude onto the profile. Note that the LCS x-axis is not exactly parallel to the gravity profile. The y-axis is obtained by a counterclockwise rotation of 54° from the North that denotes the y-axis of the Swiss coordinate system. The white rectangle indicates the dimensions of the top prism used to model the overdeepening infill for this profile. The prism dimensions are 2.4 km in width and 3.5 km in length along the valley axis (2 km towards the NW and 1.5 km towards the SE). The latter prism dimensions are supposed to approximate the main valley volumes following the model of Reber and Schlunegger 2016.*

Conducting the modelling

As a first test, we explored the effects of different density contrasts between the bedrock and the sedimentary infill of the tunnel valley. Accordingly, the first two models 1 and 2 were accomplished using the same geometry but were done with different density contrast values of -300 kg/m^3 (Figure D.4.5a) and -500 kg/m^3 (Figure D.4.5b) respectively. Note that in this context, we infer an identical density for the Lower Freshwater und Upper Marine Molasse bedrock of 2500 g/cm^3 as determined in

Bandou et al. (2022). We justify this inference because both stratigraphic groups have a comparable sedimentary architecture in the study area, where alternations of sandstones and mudstones predominate. However, the higher density contrast applied to model 2 yields a maximum gravity effect that is nearly twice the maximum amplitude of the observed residual anomaly (Figure D.4.5b). Yet also for model 2, the anomaly of the NE upper flank is perfectly reproduced by the model, which would indicate that this density contrast might be applicable for the top part of the overdeepening. This comparison suggests that a slightly higher density contrast than -300 kg/m^3 is more likely for most of the overdeepening fill (similar to Schwenk et al., 2022 for the Rehhag drilling in the Bümpliz side channel), while a density contrast of -500 kg/m^3 (inferred by Bandou et al., 2022, for the sedimentary fill of the Gürbe trough) is likely appropriate for the upper part of the overdeepening.

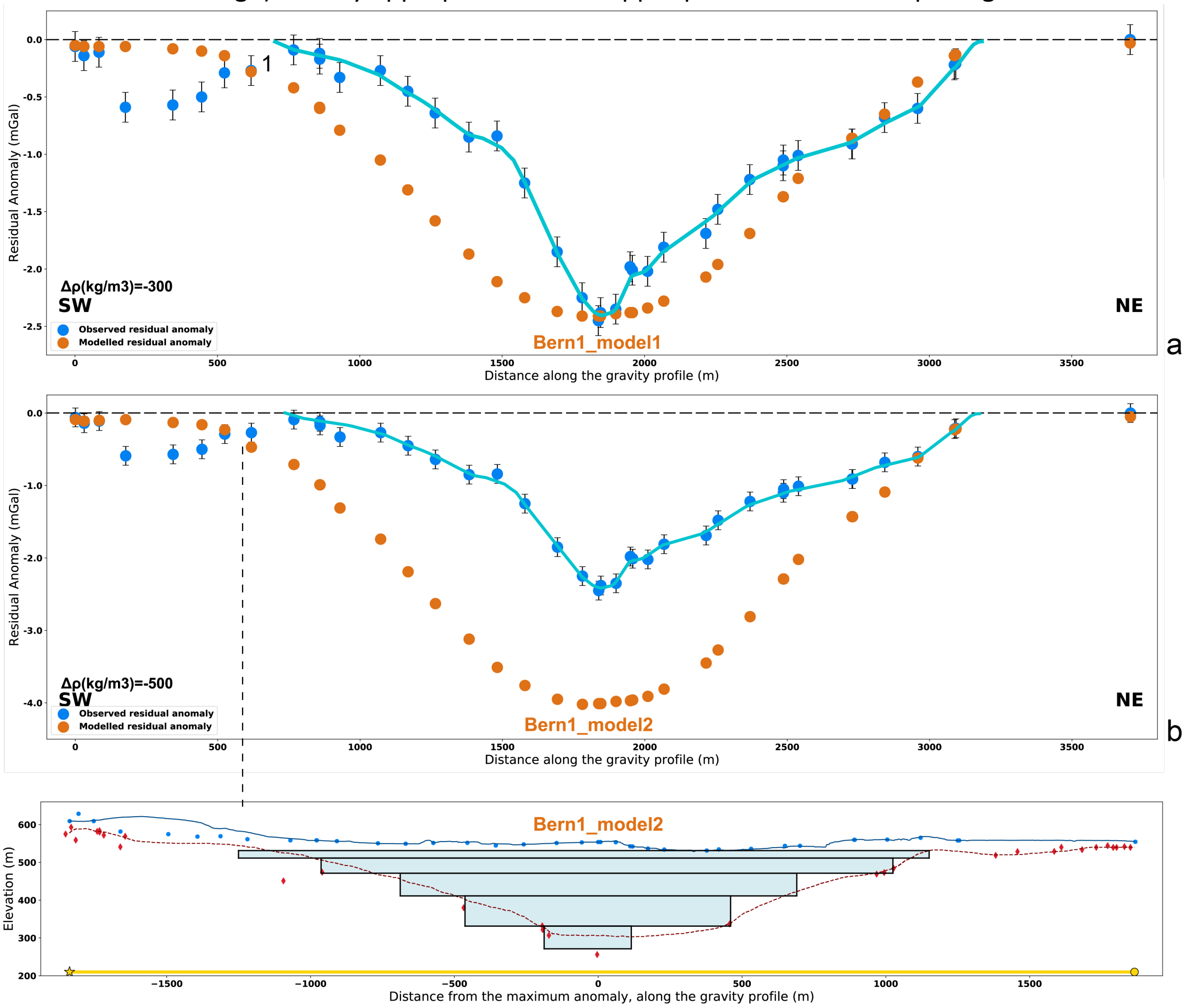

Figure D.4.5. *Initial model1 (above) and 2 (below), for the Bern1 gravity profile, made with 5 prisms with a uniform density contrast for the whole model of -300 kg/m^3 (a) and -500 kg/m^3 (b). Top: The blue dots represent the observed residual anomaly, and the orange dots are the calculated gravity values for models 1 and 2. The black bars indicate our maximum uncertainty of +/- 0.13 mGal. The light blue line highlights the main anomaly. Bottom: Elevation (SwissAlti3D 2m DEM (© swisstopo)) along the profile (blue solid line). The red broken line illustrates the bedrock topography of the model Reber and Schlunegger (2016). The blue dots are the gravity stations, the red diamonds indicate drillings that have reached the bedrock. The black rectangle shows the cross sections of the 5 prisms used. Modelling conclusions: The general U-shaped geometry used with this model shows a much wider amplitude than the measured data specially on the SW flank, while the NE flank is too wide in its deepest portion and not wide enough for its shallower section. Moreover, while the maximum anomaly is fit, our density contrast of -300 kg/m^3 is too low for the model displayed in (a). However, the use of a density contrast of -500 kg/m^3 (b) yields a modelled anomaly where the general U-shape geometry wavelength is too wide. Moreover, the higher density contrast yields a maximum amplitude that is nearly twice the observed one. However, the measured anomaly pattern on the NE upper flank is perfectly reproduced, which would indicate that this density*

contrast might be applicable for the top part of the overdeepening. The yellow start indicated the start of the profile, and the yellow circle is the end.

The approach we followed for further modelling consisted of constraining the top geometry of the trough before moving to the bottom geometry. This is visible for model 5 where the upper section has a more complex modelled geometry (Figure D.4.6). This fifth model shows a good overall fit to the wavelength and amplitude of the gravity anomaly, and to the well-defined general geometrical features of the trough. In model 5 all prisms are assumed to have the same density contrast of -350 kg/m^3. However, we note the underestimation of the gravity effect at the end of the NE flank, and the slight overestimation of the effect in the middle of the SW flank, where the gravity signal decreases sharply. This seems to indicate the end of a plateau. Yet, the modelled geometry characteristics point to the occurrence of a wide and shallow upper section and a narrow and deep lower part of the overdeepening. The misfit documents that in reality the uppermost part of the trough has a larger gravity effect than currently calculated, especially on the NE flank. This could be easily achieved by increasing the density contrast to -500 kg/m^3 for the top 2 prisms as seen in model 2 above (Figure D.4.5b) to accommodate the increase in the gravity effect on the NE flank. In addition, a reduction in the thickness of the uppermost section will contribute to the effect that the maximum gravity amplitude will not increase too much. For the narrower part of the trough, the fit can be improved by reducing the width of the prisms. The maximum anomaly is now slightly underestimated, and a further reduction of the width of the prisms will require to deepen the overdeepening to get a perfect between the modelled location of the maximum residual anomaly and the observed one.

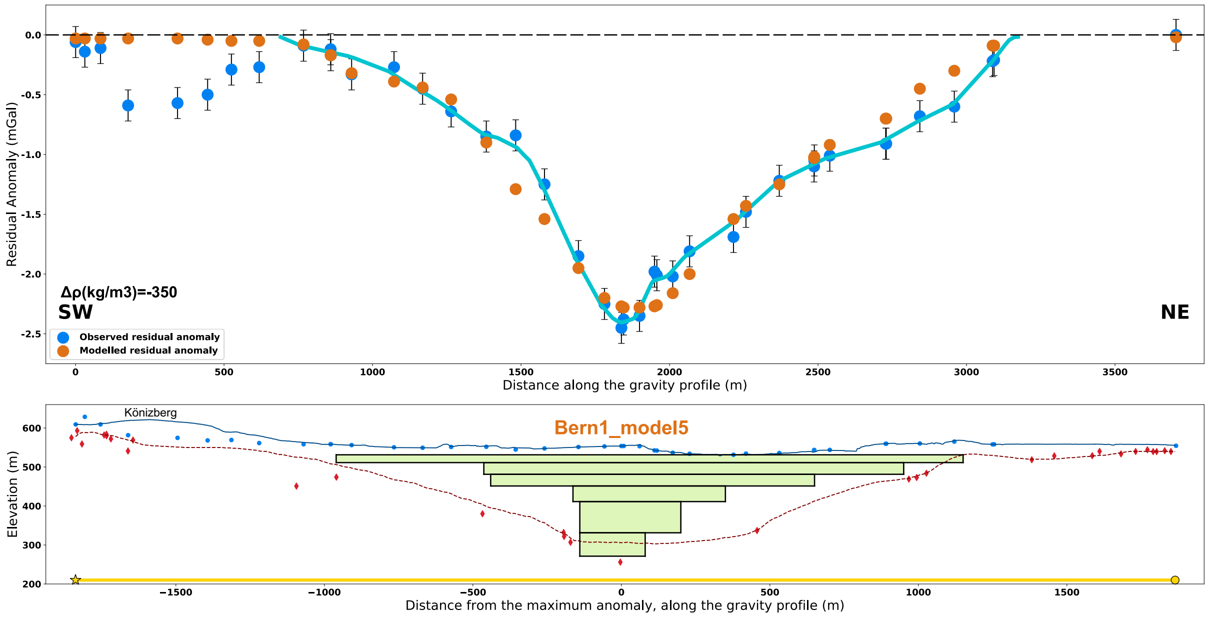

Figure D.4.6 *Intermediate model for the Bern1 gravity profile. Model 5 with 6 prisms with a uniform density contrast for the whole model of -350kg/m^3. Top: The blue dots represent the observed residual anomaly, and the orange dots are the calculated gravity values for model 5. The black bars indicate our maximum uncertainty of +/- 0.13 mGal. The light blue line highlights the main anomaly. Bottom: Elevation (SwissAlti3D 2m DEM (© swisstopo)) along the profile (blue solid line). The red broken line illustrates the bedrock topography of the model Reber and Schlunegger (2016). The blue dots are the gravity stations, the red diamonds indicate drillings that have reached the bedrock. The black rectangle shows the cross sections of the 6 prisms used.*

After fine tuning the geometry of the prisms the final model for the Bern1 profile shows a nearly perfect fit (calculated gravity effect matching the observed residual gravity within the limits of the uncertainty estimates) for most stations (Figure D.4.7). The values at some stations cannot fully be reproduced with the model, which we explain by the fact that side channels and meanders introduce a complexity that cannot be considered by prisms alone. Furthermore, we also note that some drillings are far off the modelled solution, particularly for the deeper V-shaped part of the overdeepening. We

explain this misfit by the erosional mechanism, which was most likely accomplished with water (due to the V-shaped character, see discussion in the main text). Bedrock incision by water results in the formation of meanders, deep and narrow gorges, and sharp turns at short downstream distances, which cannot be reproduced by prisms simply following the longitudinal trend of the main channel depression. Therefore, while our gravity models perfectly reproduce the overall character of the overdeepening's geometry for the cross section (i.e. wide and U-shaped top and a narrow V-shaped, and deep base), it fails to reproduce meanders, side channels and local features that occur over short down-stream distances.

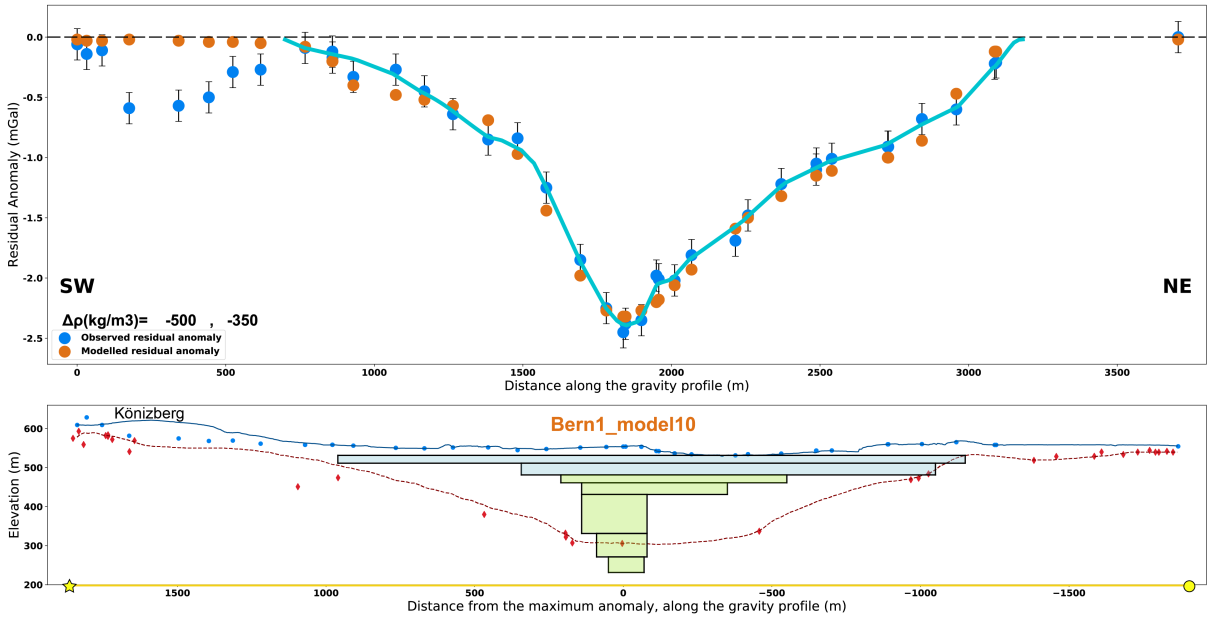

Figure D.4.7 *Final model for the Bern1 profile, model 10 made with a total of 7 prisms with a density contrast of -500 kg/m^3 (light blue) for the top two prisms and -350 kg/m^3 (light green) for the rest of the prisms. Top: The blue dots represent the observed residual anomaly, and the orange dots are the modelled residual anomaly values for model 10. The black bars indicate our maximum uncertainty of +/- 0.13 mGal. The light blue line highlights the main anomaly. Bottom: Elevation (SwissAlti3D 2m DEM (© swisstopo)) along the profile (blue solid line). The red broken line illustrates the bedrock topography of the model Reber and Schlunegger (2016). The blue dots are the gravity stations, the red diamonds indicate drillings that have reached the bedrock. The black rectangle shows the cross sections of the 7 prisms used.*

D.5 The Bern2 profile

<u>Data collection and estimation of regional gravity gradient, and estimation of residual anomalies</u>

The Bern2 profile is oriented from the SSW to the NNE cutting across the city of Bern (Figure D.1). At either end of the profile the bedrock has been documented to be very near to the surface, thanks to the numerous drillings in both areas. The northern part of the profile runs through the historical part of the city of Bern and the stop for trams and buses next to the Bern main train station. This offered an extra challenge due to the high traffic, disturbing the gravity measurements but also due to the tall buildings interfering with the reception of a good GPS signal. This led to a few larger gaps between the stations in the NNE half of the profile (Figure D.5.1).

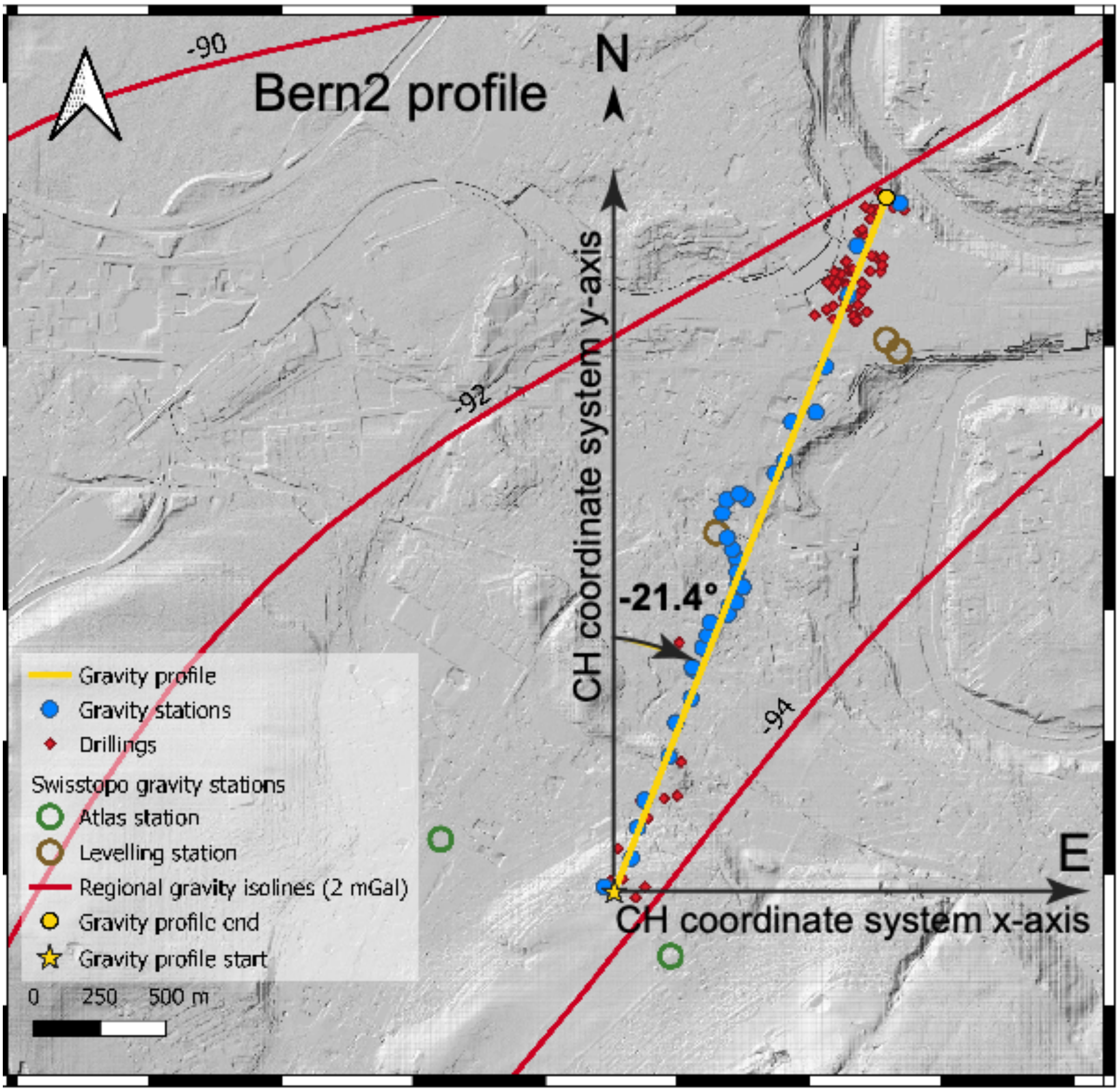

Figure D.5.1 *This map shows the SwissAlti3D 2m DEM (© swisstopo) of the area of the Bern2 profile, where the yellow line denotes the gravity profile. The yellow star and dot indicate the start and the end of the profile, respectively. The green circles represent the gravity stations of the Gravimetric Atlas of Switzerland, and the red lines denote the regional gravity isolies (Olivier et al., 2008, 2011). The blue dots are the 39 newly measured gravity stations. The red diamonds show the location of the drillings that reached the Molasse bedrock. This profile runs obliquely to the -92 mGal and -94 mGal regional gravity isoliness.*

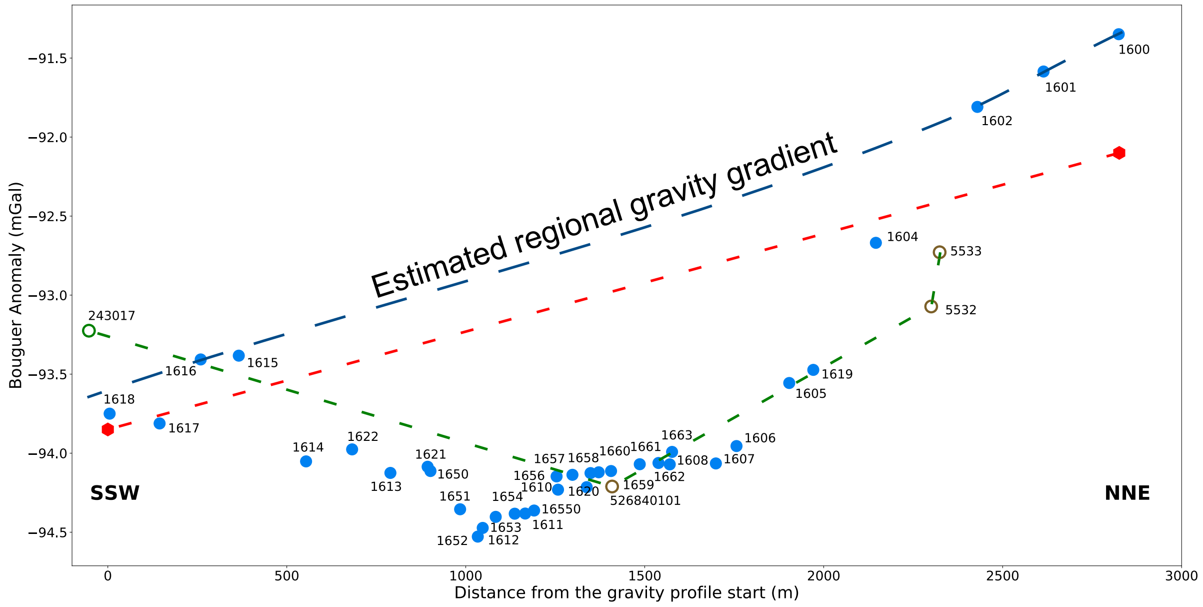

Figure D.5.2 *Bouguer anomaly along the Bern2 profile with the 35 newly measured gravity stations (blue solid dots). The green broken line denotes the local gravity anomaly along the profile as estimated based on points of the Gravimetric Atlas of Switzerland (Olivier et al. 2008; 2011, shown as green circles) and from levelling projects (brown circle). The red broken line denotes the linear interpolation from the regional map isolines (-94 mGal and -92 mGal; Figure D.5.1). We note that the value at the station 526840101 (brown circle near 1400 m profile distance) correlates well with the gravity value of our new station 1609. We estimated a regional gravity gradient (blue broken line) using MPs 1615 and 1616 in the SSW and MPs 1600, 1601 and 1602 in the NNE profile ends as references.*

The local gravity anomaly caused by sedimentary fill of the overdeepening beneath the Bern2 profile seems to extend from MP 1615 to MP 1602 (Figure D.5.2). A local gravity low is also visible from just the few gravity stations from the swisstopo database (Figure D.5.2, green circles connected by green broken line). The estimated regional gravity gradient was defined using the information offered by the stations 1600 to 1602 for the NNE part whereas stations 1618, 1616 and 1615 were used for the SSW part. These points align well with the trend given by the regional information, and their locations on either side of the profile also correspond well with the bedrock at very shallow levels as constrained by the drillings (Figure D.5.3). Upon subtracting the estimated regional gravity gradient (broken blue line in Figures D.5.2 and D.5.3) from the Bouguer gravity we obtain the residual gravity along the Bern2 profile (Figure D.5.3). The local residual gravity anomaly has a wavelength of about 2.1 km and it is approximately U-shaped with two locations separated by about 700m showing a maximum amplitude of -1.6 mGal. We note that the SSW flank of the anomaly is less uniform than the NNE flank, where Bouguer gravity values change in steps.

On closer inspection of the gravity field, we conclude that there probably exist two gravity anomalies of significantly different wavelengths that overlap (Figure D.5.3). The longer wavelength anomaly (blue solid line) suggests the occurrence of a wide somewhat asymmetric trough reaching a maximum depth at around 1700 m profile distance. The shape of this long wavelength anomaly closely corresponds to the geometry proposed by Reber and Schlunegger (2016). The short wavelength (with of approximately 350m) local anomaly (solid red line) reaching an additional -0.5 mGal (relative to the longer wavelength anomaly, see Figure D.5.3) near profile distance 1000 m suggests that there exists a significant (deep and narrow) local bedrock depression underneath the otherwise rather smoothly and gently dipping SSW flank of the main trough.

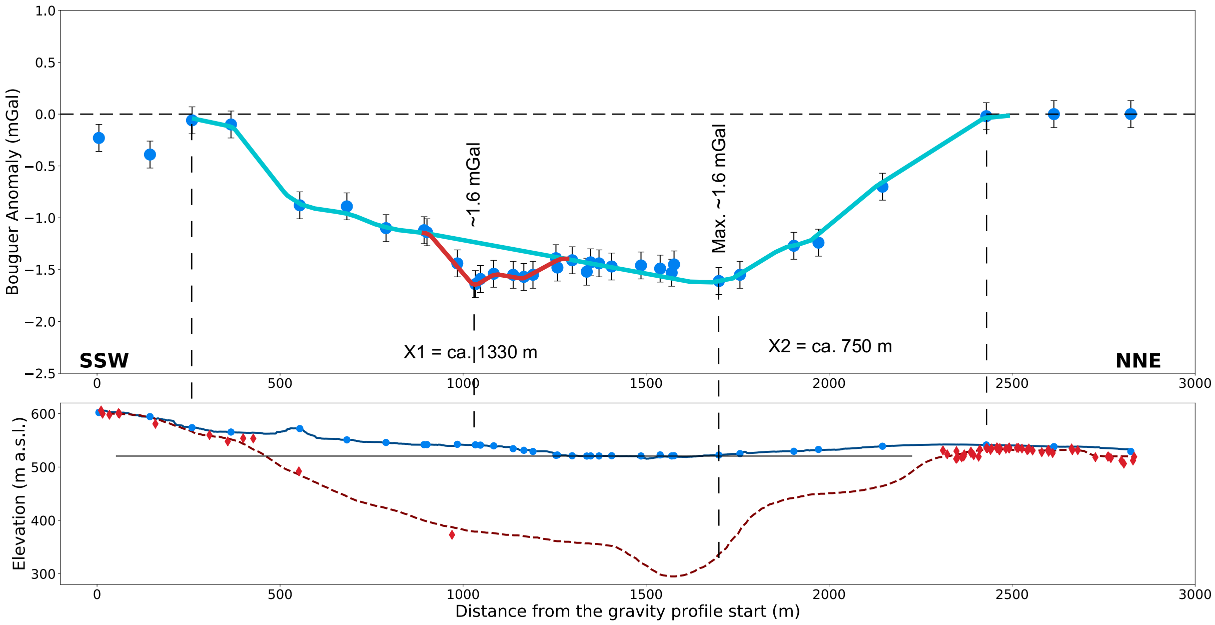

***Figure D.5.3** Top: Residual anomaly for the Bern2 profile. The blue dots represent the residual anomaly calculated for the stations; the black bars are error bar of +/- 0.13 mGal. The residual anomaly was estimated using the regional gravity gradient and using the Bouguer anomaly from Figure D.5.2, the light blue line denotes the overdeepening effect. Bottom: Bottom: Elevation profile (SwissAlti3D 2m DEM (© swisstopo)) and bedrock profile (Reber and Schlunegger, 2016) of the Bern2 section. The blue dots are the gravity stations, the red diamonds indicate key drilling information for the determination of the regional gravity gradient.*

Modelling the residual anomalies of the Bern2 profile

We placed the top prims for modelling using the same strategy as for the Bern1 profile (see section D.4). However, the drilling information and the bedrock model from Reber and Schlunegger (2016) indicate that the near-surface sediment cover building the topographic volumes above the top prism of the main tunnel valley (Figure D.5.4) belongs to the Quaternary sediments, creating the step-topography we observe on both flanks. Due to their proximity to the gravity stations just above them, the effects of these two sedimentary volumes need to be taken into account upon modelling. For technical reasons regarding the PRISMA code, these sedimentary volumes related to the NE and SW surface topography need to be modelled separately. The models show (Figure D.5.4, for further details see Bandou, 2023) that the effect of the sedimentary cover above 520 m can reach up to 0.84 mGal, and this contribution is clearly not negligible to model the geometry of the rest of the overdeepening. The subsequent modelling steps were conducted in a similar way and using the same logics as for the Bern1 profile (see section D.4): We started with an initial model of the main bedrock trough, aiming to reproduce the shape of the main residual anomaly with a uniform density contrast between the Molasse bedrock and the Quaternary fill of -500 kg/m^3 (Bandou et al., 2022). The results clearly documented that this density contrast is too large for the middle and lower sections of the trough. For the following models, we adjusted the density contrast to -350 kg/m^3 for all the prisms except the top one, which remains at the same density contrast value of -500 kg/m^3. We then adjusted the geometry of the prisms upon modelling until a best fit between model results and observations was reached. We thereby considered the contribution of the topography effect and adjusted the iteratively adjusted the width of the prims to improve the fit between model results and observations, which finally resulted in model 10 (Figure D.5.5). However, the local gravity anomaly of about -0.3 mGal and possibly about 200 m wavelength remains unexplained by the main trough sedimentary infill of model 10. This signal could likely be caused by a sedimentary fill of an inner gorge structure in the bedrock, which would be a situation that is similar to the Aare gorge near Innertkirchen some 75 km upstream.

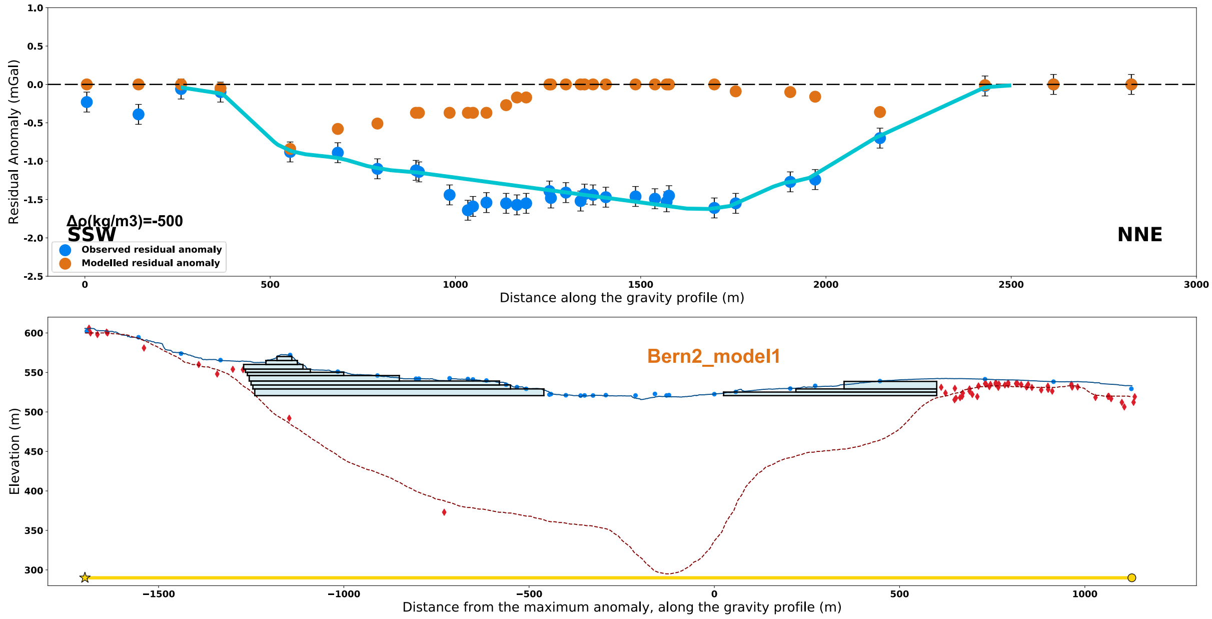

Figure D.5.4 *Model 1 of the Bern2 profile, showing the effects of (i) the SW surface topography model onto 13 stations within the SW valley side and of (ii) the NE surface topography model onto 5 stations within the NE valley side. Their elevation is significantly higher than that of the lowest gravity station along profile. Top (vertical exaggeration): The blue dots represent the observed residual anomaly, and the orange dots are the modelled residual anomaly values for (i) the SW surface topography model with 9 thin prisms and (ii) the NE surface topography model with 3 thin prisms. Bottom: Elevation profile (SwissAlti3D 2m DEM (© swisstopo)) and bedrock profile (Reber and Schlunegger, 2016) of the Bern2 section. The blue dots are the gravity stations, the red diamonds indicate drillings that have reached the bedrock. The black rectangles show the cross sections of the 8 prisms used.*

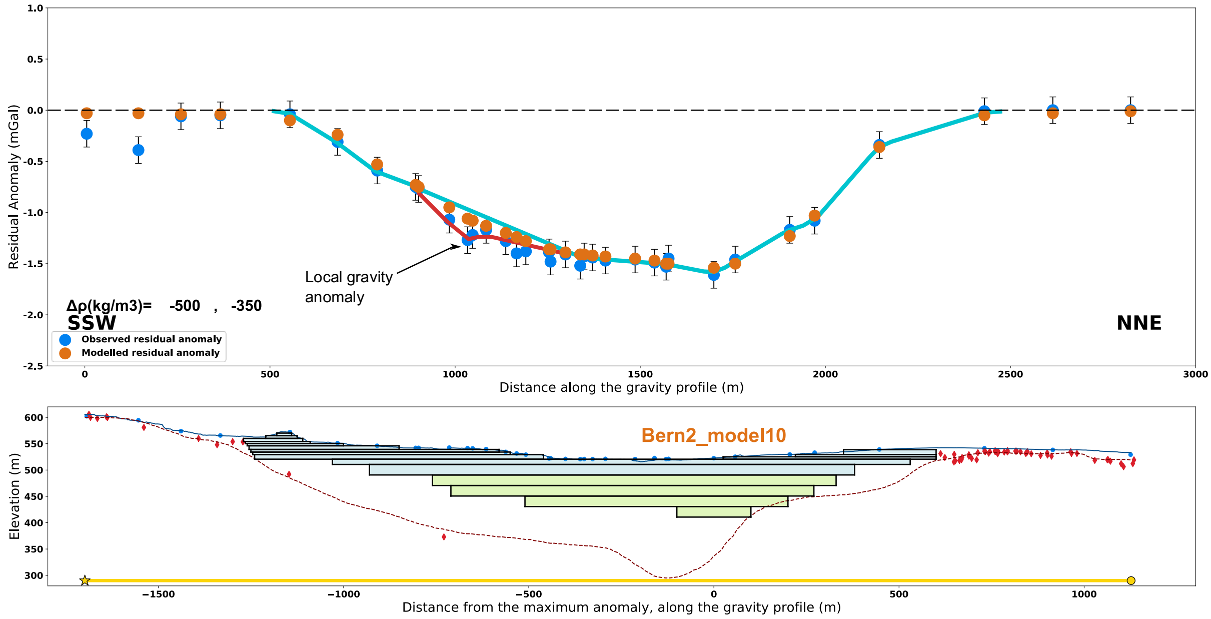

Figure D.5.5 *Results of the model 10 for the Bern2 profile, which yields a best fit for the overall geometry of the overdeepening fill. Two different density contrasts are used, the two top prisms and the side topography prisms have a contrast of -500 kg/m³ (light blue), while the other 4 prisms have a density contrast of -350 kg/m³ (light green). Top: The blue dots represent the observed residual anomaly, and the orange dots are the modelled residual anomaly values for model 10. The black bars indicate our maximum uncertainty of +/- 0.13 mGal. The light blue line highlights the main anomaly and the red line shows the secondary anomaly. Bottom: Elevation profile (SwissAlti3D 2m DEM (© swisstopo)) and bedrock profile (Reber and Schlunegger, 2016) of the Bern2 section. The blue dots are the gravity stations, the red diamonds indicate drillings that have reached the bedrock.*

In order to model such an inner gorge structure, we introduced a new prism extending along the main channel axis 200 m in both directions from the profile. We first select a width of c. 40 m, a value which is based on the largest width of the Aare gorge, and we use the thickness of the fifth prism as a first very conservative estimate (model 11, inner gorge model A, see Fig. 20). We then adjusted the widths and thicknesses of the prisms until a best-fit was reached between model results and observations. This finally yielded in model 13 (Figure D.5.6), which would be a reasonable estimate for the geometry of the inner gorge. However, drilling data (e.g., at the Marzili along the Bern4 profile, see section D.8) shows the inner gorge could possibly be deeper.

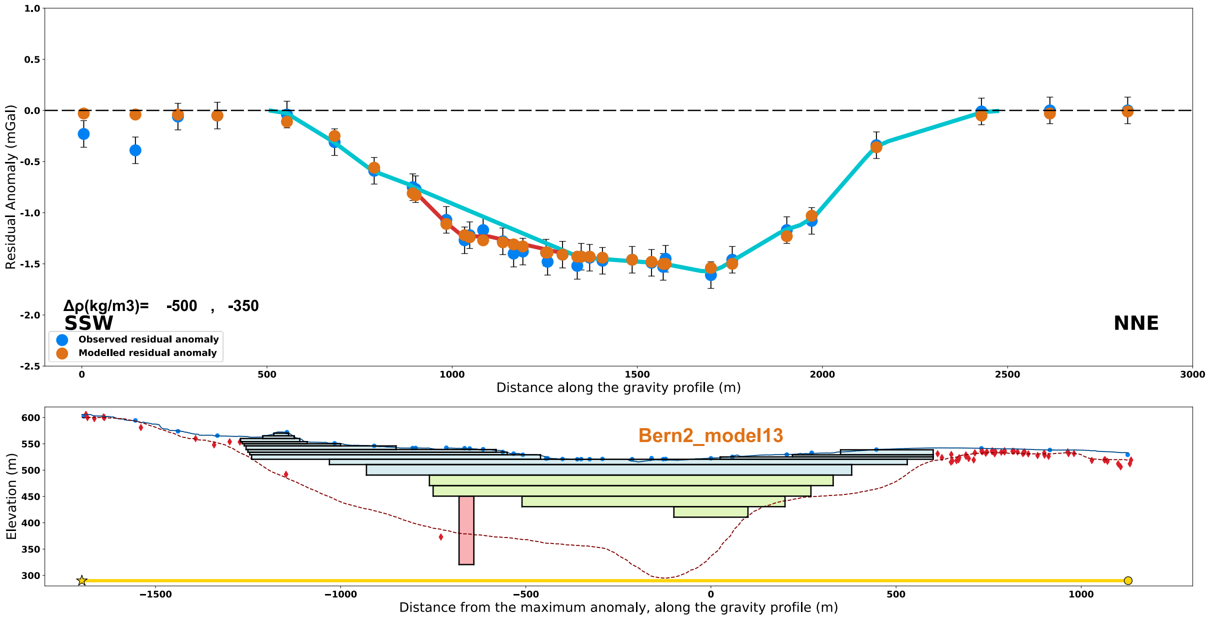

Figure D.5.6. *Inner gorge model. See legend to figures above for explanation of the symbols.*

Further modelling considering a narrower but deeper inner gorge yields the same results. The model uncertainties are quite large, documenting that we have reached the limit offered by the selected approach.

D.6 The Kehrsatz profile

Data collection and estimation of regional gravity gradient, and estimation of residual anomalies

The Kehrsatz gravity profile traverses the Aare valley in the center of the study area (Figure D.1) where the model by Reber and Schlunegger (2016) shows a narrowing of the tunnel valley relative to the geometry farther upstream.

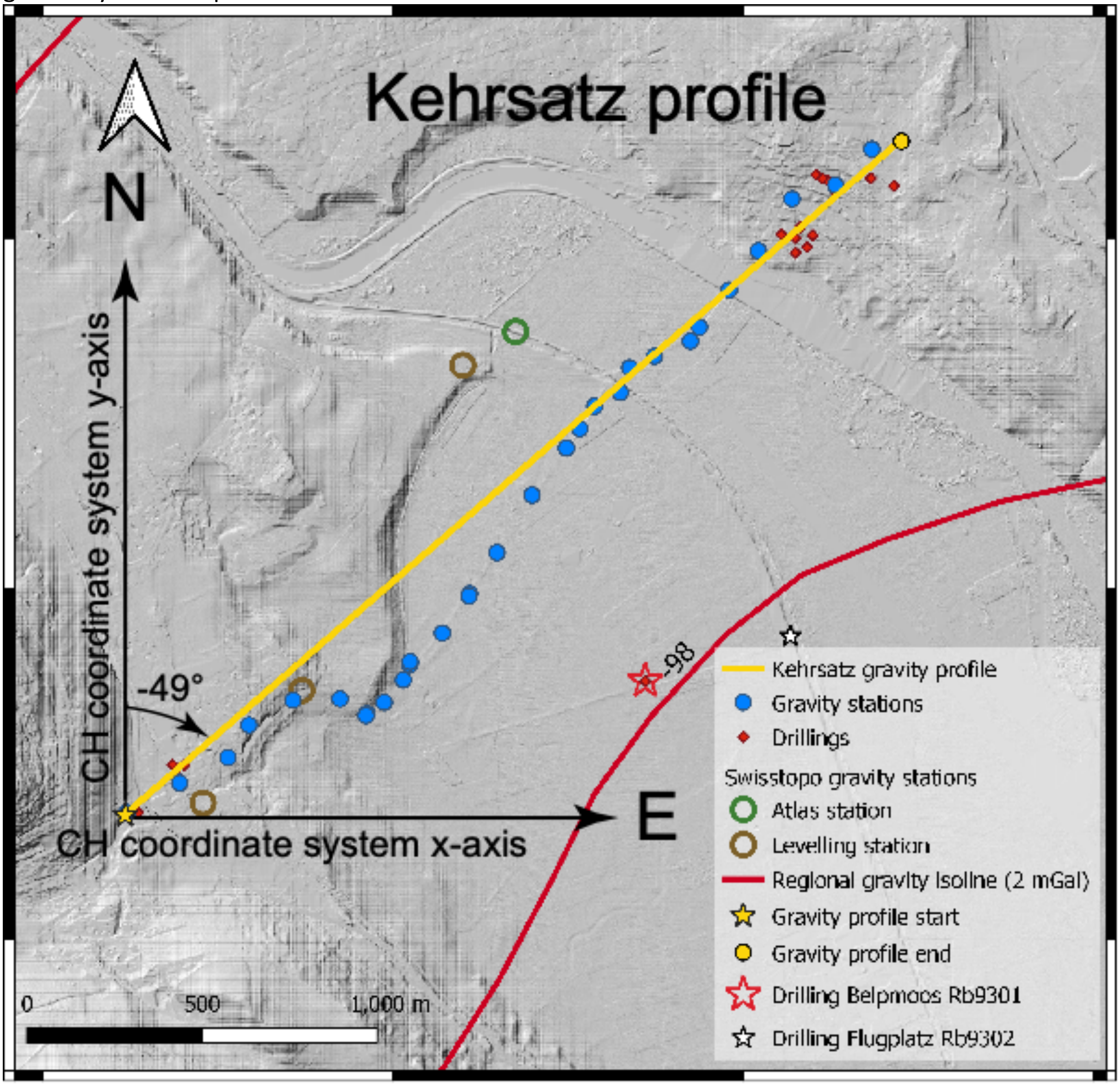

Figure D.6.1 *Details of the regional Bouguer gravity map (green circles representing the gravity stations and red lines denote the regional gravity isolines) showing the vicinity of the Kehrsatz gravity profile (yellow line, see Figure D.1). The yellow star and dot indicate the start and end of the gravity profile, respectively. There exists only a limited number of stations (green circles) from the Gravimetric Atlas of Switzerland © swisstopo (Olivier et al., 2008; 2011) and one levelling station (in brown) close to the profile end. The blue dots are the 22 newly measured gravity stations. The red diamonds show the location of the drillings that reached the Molasse bedrock. The red star highlights the deepest drilling that reached the bedrock, yet it is located far from the profile. The white star indicates the location of the deepest drilling, reaching 200 m beneath the surface, but it did not encounter the bedrock. The gravity profile runs obliquely to the -96 mGal and -98 mGal regional gravity isolines. The regional gravity isolines already document the presence of the negative anomaly caused by the overdeepenings.*

The Kehrsatz gravity profile (Figure D.6.2 and D.6.3) shows two local anomalies: a smaller anomaly on the SW side and a larger one in the center part. A third anomaly starts on the NE end of the profile. We attribute the difference in the gravity between MP 5220 and MP5204 around 700 m profile

distance to the lateral offset of some points from the profile line in combination with the effects of the nearby topography that forms a thick terrasse.
We estimate the regional gravity gradient using the information of the MPs 5200 and 5201 on the SW flank, as their values indicate that they are located above the bedrock and that they do not record the effect of the valley. Likewise, on the NE flank the station 5215 is used to constrain the regional gravity gradient. Note that these stations consistently exhibit Bouguer gravity values that are about 0.5 mGal higher than the regional gravity trend (Figure D.6.2, red broken line).

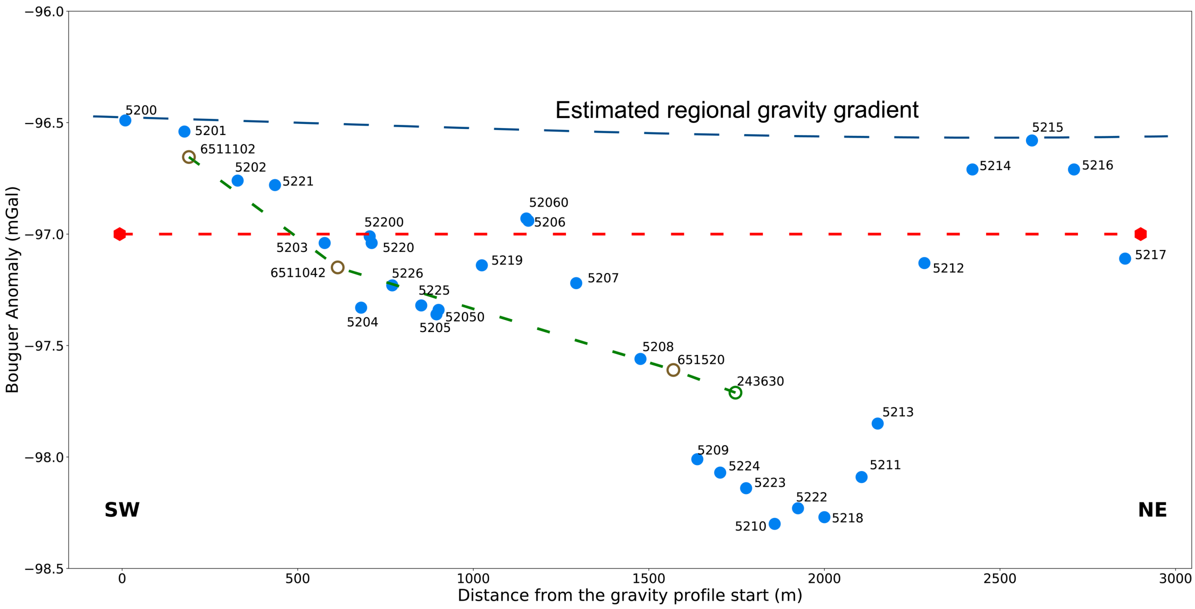

Figure D.6.2 *Bouguer anomaly along the Kehrsatz gravity profile with the 30 newly measured stations (blue solid dots). The green broken line denotes the local gravity anomaly along the profile as estimated based on points of the Gravimetric Atlas of Switzerland (Olivier et al. 2008; 2011, shown as a green circle) and from levelling projects (brown circle). The red broken line denotes the linear interpolation from the regional map isolines (-96 mGal and -98 mGal; Figure D.6.1). Note that no additional data was collected close to the station from the gravimetric atlas 24-3630 (243630). In relation to the estimated regional gravity field (broken blue line), a maximum amplitude of c. -1.7 mGal is measured at MP 5210.*

The 30 gravity stations of the Kehrsatz provide us with a residual anomaly consisting of two parts (Figure D.6.3). A shorter wavelength and lower-amplitude anomaly of about -0.8 mGal on the SW side between c. 500 m and c. 1150 m profile distance, mostly U shaped, and a main anomaly between c. 1150m to c. 2600 m profile distance reaching a maximum amplitude of -1.7 mGal. This main anomaly shows a nearly U-shaped geometry with a pronounced asymmetry as the NE flank appears to be steeper. In a few aspects the residual gravity anomaly diverges from the proposed bedrock geometry (Figure D.1) by Reber and Schlunegger (2016), possibly due to the lack of deep drilling information nearby the profile. The main differences regard the existence of a side trough and the location of the deepest part of the trough, which is situated farther to the NE based on the gravity information.

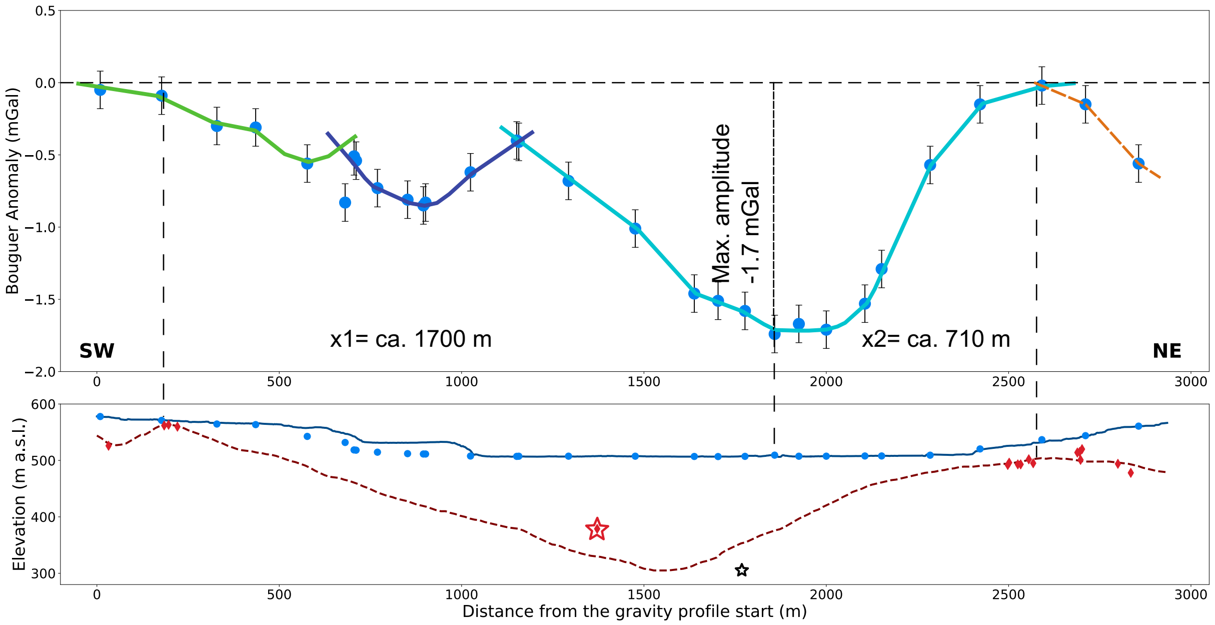

***Figure D.6.3** Top: Residual anomaly for the Kehrsatz profile. The blue dots represent the residual anomaly calculated for the stations; the black bars represent maximum estimated errors of +/- 0.13 mGal* (Bandou et al., 2022)*. The residual anomaly was calculated subtracting the estimated regional gravity gradient from the local Bouguer anomaly. The light blue line summarizes the residual gravity anomaly representing the effect of the sediments in the main channel of the overdeepening, the dark blue line highlights the effect of the side channel and the green line represents the topographic signal of the SW flank. Bottom: Surface topography (SwissAlti3D 2m DEM (© swisstopo)) along the profile (blue solid line) and elevation of the gravity stations (solid blue dots). The red broken line illustrates the bedrock topography of the model by Reber and Schlunegger (2016). The red diamonds indicate the position the drillings that have reached the bedrock. The mostly U-shaped main residual anomaly of -1.7 mGal shows a slight asymmetry between the steeper NE flank and the SW flank. The shorter wavelength and lower-amplitude anomaly on the SW side is also asymmetric and mostly U-shaped.*

We placed the top prims for modelling using the same strategy as for the Bern1 profile (see section D.4). However, the drilling information and the bedrock model from Reber and Schlunegger (2016) indicate that the near-surface sediment cover building the topographic volumes above the top prism of the main tunnel valley belongs to the Quaternary sediments, creating the step-topography we observe on both flanks. We considered this effect in the same way as for the Bern2 profile (see section D.5). Upon modelling, we then first adjusted the density contrasts between the Molasse bedrock and the Quaternary fill and corrected the widths of the prisms using the same logics as outlined for the Bern2 profile (section D.5). We ended with model 6 (Figure D.6.4), which consists of two depressions separated by a local bedrock ridge c. 15 m beneath the surface. The side depression, on the SW, is shallow with a depth of 40 m, and it shows a U-shaped cross-sectional geometry. The cross-sectional geometry of the main depression is also U-shaped with a wide and flat bottom. It has an asymmetry with a flank that is steeper on the NE than on the SW side. This final model 6 shows significant differences to the bedrock model of Reber and Schlunegger (2016), where the lack of deep borehole data at this locality leads these authors to propose a much wider and deeper overdeepening, with a maximum depth located farther to the SW.

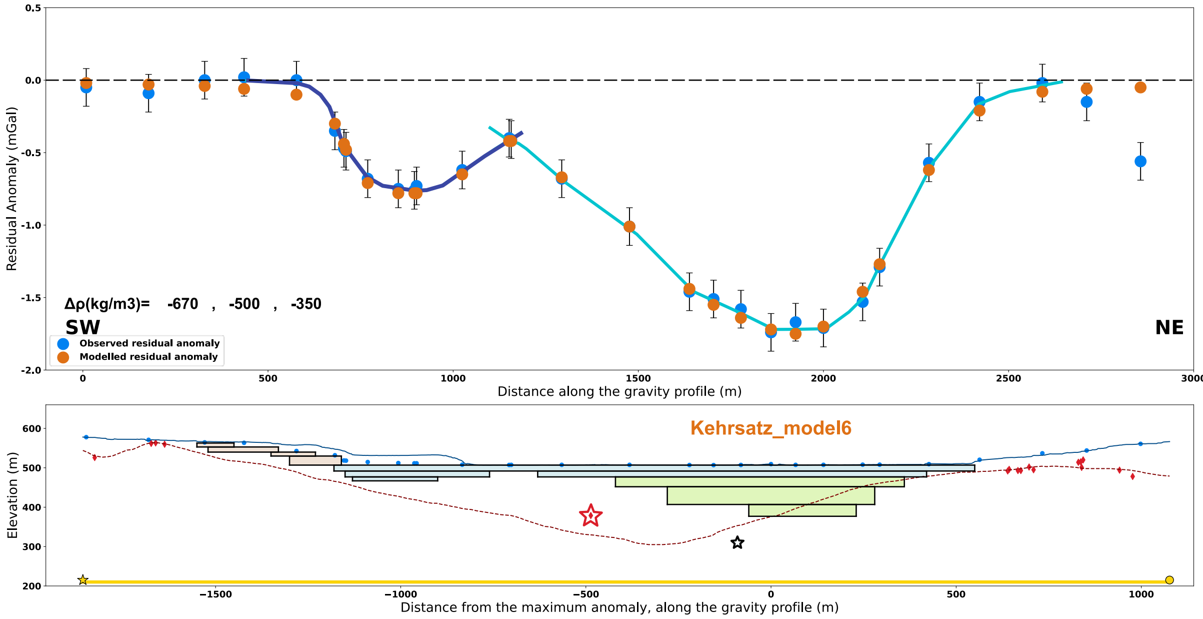

Figure D.6.4 *Final model 6 of the main valley for the Kehrsatz gravity profile. This model is made with 7 prisms, of which 4 have a uniform density contrast of -500 kg/m^3 (light blue) and 3 have one of -350 kg/m^3 (light green) between the Molasse bedrock and the overdeepening fill. The 4 prisms of the SW side topography are shown in light brown. There were assigned a density contrast of -670 kg/m^3 because these prisms consider the topography effect on the residual gravity anomaly. Because standard topography corrections are commonly accomplished using a density of 2670 kg/m^3, the occurrence of a hill made up of Quaternary sediments with a much lower density will result in a negative Bouguer anomaly signal, which has to be corrected accordingly. Top: The blue dots represent the observed residual anomaly, and the orange dots are the calculated gravity values for model 6. The black bars indicate our maximum uncertainty estimates of +/- 0.13 mGal. The light blue line highlights the main residual gravity anomaly corrected for the effect of the SW side topography, see model 10 and the dark blue line highlights the side channel's anomaly. Bottom: Elevation (SwissAlti3D 2m DEM (© swisstopo)) along the profile (blue solid line). The red broken line illustrates the bedrock topography of the model by Reber and Schlunegger (2016). The blue dots are the gravity stations, the red diamonds indicate drillings that reached the bedrock. The yellow star and point indicate the start and end of the gravity profile, respectively. The stars show drillings located > 900 m away from the profile, the red one for the Rb9201 drilling while the white one shows the Rb9202 drilling that did not reach the bedrock. The black rectangles filled in light blue show the cross sections of the 7 prisms of model 6.*

D.7 The Airport profile

<u>Data collection, estimation of regional gravity gradient, and estimation of residual anomalies</u>

The Airport profile is located to the South of the city of Bern, runs perpendicularly to the Aare valley and crosses the Gürbe and Aare Rivers (Figure D.1). Due to logistic reasons, the gravity stations could not always be placed closely along the line profile. In particular, the centre of the valley hosts the Bern Airport (hence the name of the profile) which forced us to shift the position of the stations farther to the South of the profile (Figure D.7.1).

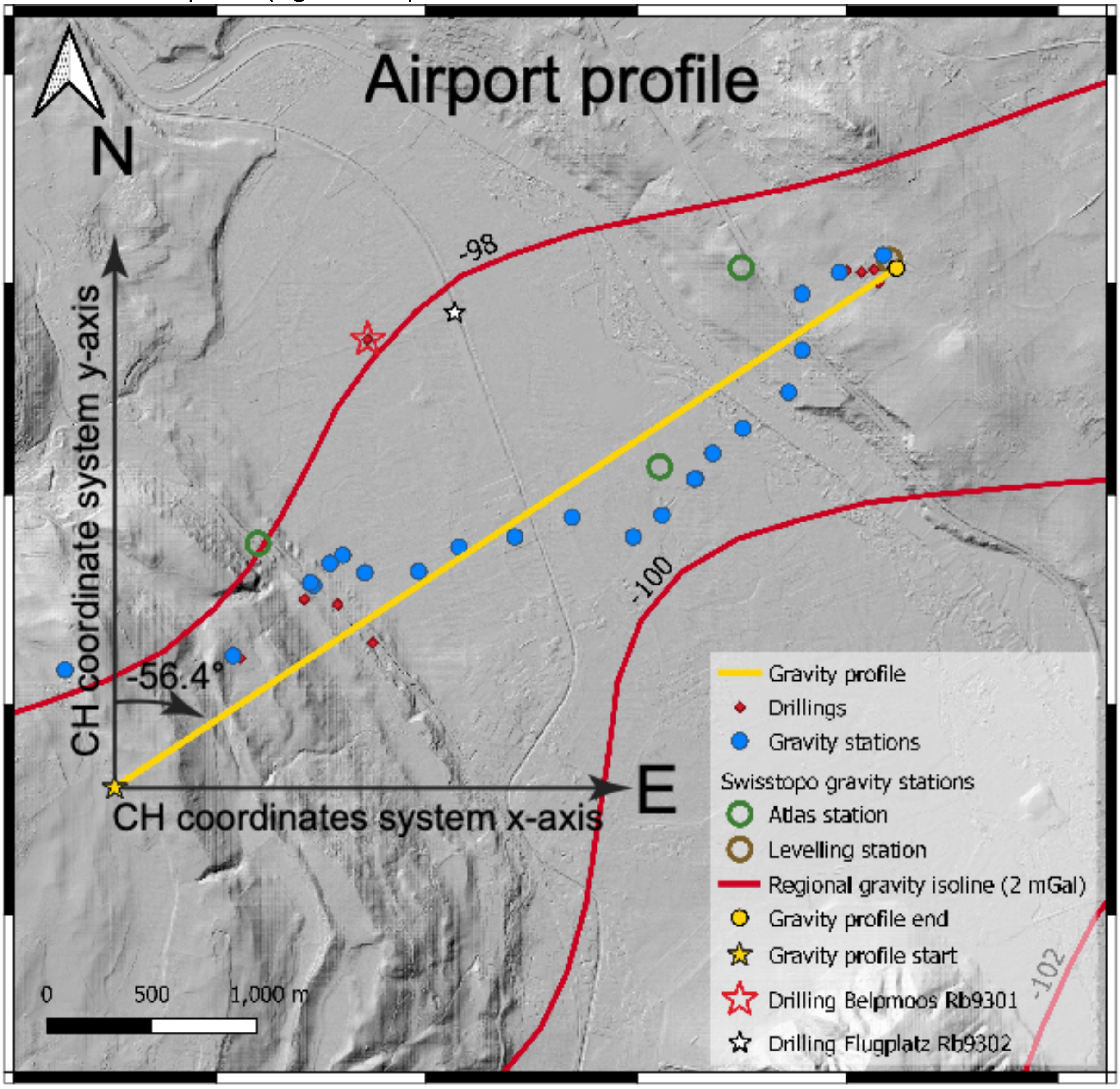

Figure D.7.1 *Enlarged extraction of regional Bouguer gravity map (green circles representing the gravity stations and red lines denote the regional gravity isolines) showing the vicinity of the Airport gravity profile (yellow line). The yellow star and dot indicate the start and end of the gravity profile, respectively. There exists only a limited number of stations (green circles) from the Gravimetric Atlas of Switzerland © swisstopo (Olivier et al., 2008; 2011) and one levelling station (in brown) close to the profile end. The blue dots are the 22 newly measured gravity stations. The red diamonds show the location of the drillings that reached the Molasse bedrock. The red star highlights the deepest drilling that reached the bedrock, yet it is located far from the profile. The white star indicates the location of the deepest drilling, reaching 200m beneath the surface, but it did not encounter the bedrock. The gravity profile runs obliquely to the -98 mGal and -100 mGal regional gravity isolines. The regional gravity isolines already document the presence of the negative anomaly caused by the overdeepenings.*

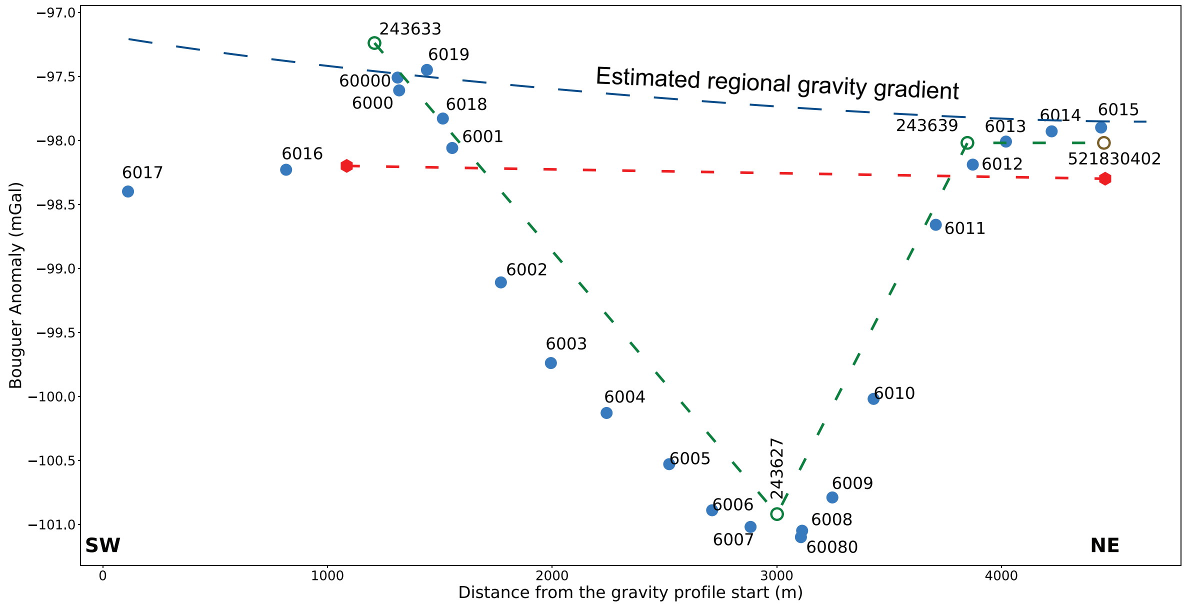

Figure D.7.2 *Bouguer anomaly along the Airport gravity profile with the newly 22 measured stations (blue solid dots). The green broken line denotes the local gravity anomaly along the profile as estimated based on points of the Gravimetric Atlas of Switzerland (Olivier et al. 2008; 2011, shown as green circles) and from levelling projects (brown circle). The red broken line denotes the linear interpolation from the regional map isolines (-98 mGal and -100 mGal; Fig. 3). We note that the values of the 3 atlas stations (green circles) correlate well with the newly measured gravity values at the nearby stations (blue solid dots). This allows us to integrate our new gravity profile into context of the regional gravity field. We estimated a regional gravity gradient (blue broken line) using MP 6019 and MP 6015 as references, where the former is the least negative value on the SW flank. The values of the stations on the NE flank indicate that the local anomaly from the valley seems to end around MPs 6013 to 6015. In relation to this regional gravity field, a maximum amplitude of c. 3.3 mGal is measured at MP 60080.*

The Bouguer anomaly profile for the Airport profile shows a wide V-shape anomaly, which is additionally asymmetric with a steeper NE flank. The three atlas stations indicate very well the occurrence of an overdeepening recorded by stations 243627 (Figure D.7.2), documenting a value that is very close to the maximum amplitude found by the new gravity measurements. Two local gravity anomalies are identifiable along the Airport profile (Figure D.7.3). The first one, situated on the SW side, has an amplitude of c. -1.2 mGal and is caused by the Molasse bedrock of the Längenberg, which is a mountain ridge to the West. The Molasse bedrock exhibits a bulk density of 2500 kg/m^3 (Bandou et al. 2022) and, consequently, standard Bouguer gravity topography corrections calculated with 2670 kg/m^3 will results in a negative local gravity anomaly at the two stations located on the hillslope of the mountain ridge made up of Molasse bedrock. A second anomaly, which we identify as the main anomaly, documents the effect of the sedimentary infill of the valley's overdeepening with a maximum amplitude of -3.3 mGal. This large local anomaly extends from c. 1500 m to c. 3900 m distance along the profile. The maximum amplitude of c. -3.3 mGal is measured at the MP 6008 situated at c. 3100 m distance (Figure D.7.3). The main anomaly is V-shaped and asymmetric. It has a relatively steep NE flank and a flatter but almost concave SW flank. The data additionally suggest that the deepest part of the overdeepening is shifted to the NE compared to what is proposed by the bedrock model of Reber and Schlunegger (2016). Moreover and also contrarily to the bedrock topography model of Reber and Schlunegger (2016), the Bouguer anomaly shows that the flank of the valley dips much more steeply on the NE side, and that the flank is not uniformly dipping towards the centre on the SW side. The bedrock model interpretations of the aforementioned authors were likely made through a linear inter- and extrapolation because of the scarcity of information on deep drillings along this section of the Aare valley. For this same reason, we will take into account two drillings located > 900m from the profile (Figure D.7.1) as they might still provide useful constraints upon modelling.

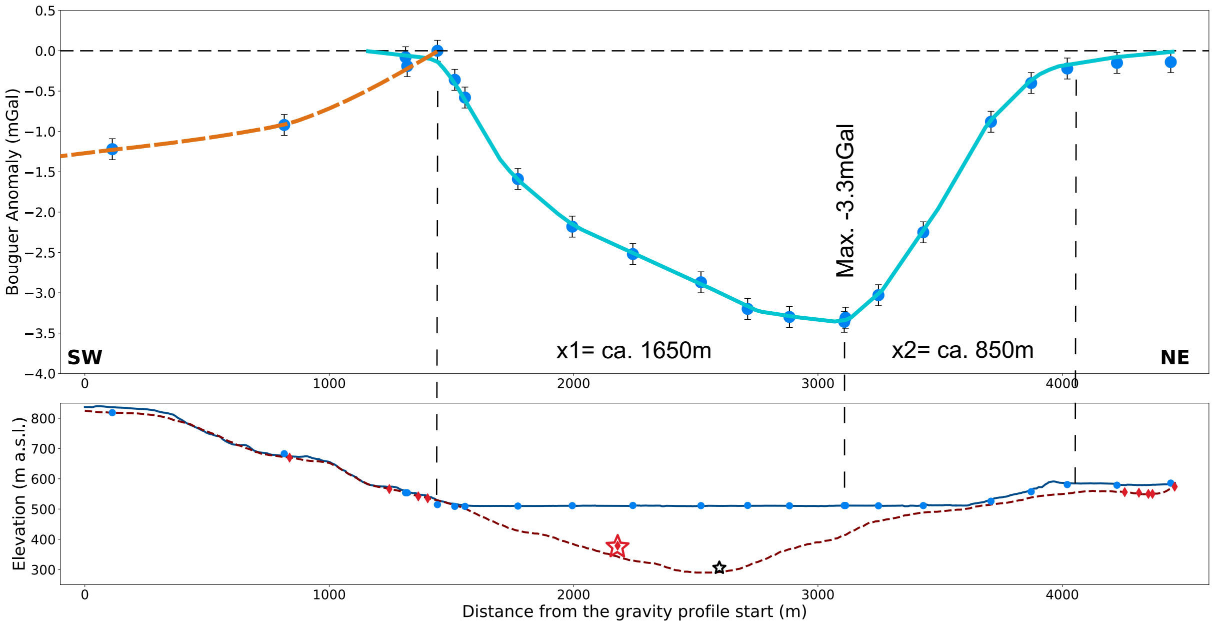

Figure D.7.3 *Top: Residual anomaly for the Airport South profile. The blue dots represent the residual anomaly calculated for the stations; the black bars are error bar of +/- 0.13 mGal. The residual anomaly was calculated by subtracting the regional gravity gradient from the Bouguer anomaly (figure 5). The light blue line denotes the overdeepening effect. Bottom: Surface topography (SwissAlti3D 2m DEM (© swisstopo)) along the profile (blue solid line) and elevation of the gravity stations (solid blue dots). The red broken line illustrates the bedrock topography of the model Reber and Schlunegger (2016). The red diamonds indicate the position the drillings that have reached the bedrock.*

Modelling the residual anomalies of the Airport profile

Preparation of the models

The lack of drilling information on the NE flank and the discrepancy between shape and extent of our residual anomaly and the bedrock model of Reber and Schlunegger (2016) led us to only use the information offered by the SW flank to define the valley direction. We therefore used the orientation of the mountain ridge's flank along this flank to constrain the direction of the main tunnel valley to be modelled (Figure D.7.4). The valley width along the profile is defined by the surface topography, by the shallow bedrock information from Reber and Schlunegger (2016) and using the information provided by the gravity data. On both sides, therefore, we placed it close to the stations that are clearly affected by effect of the overdeepening fill. Moreover, according to our modelling strategy, we designed the initial model such as to slightly overestimate the extent of the valley width. This allowed us then reduce it in the subsequent models.

The northern boundary of the top prism is defined by the bedrock topography on the NE corner. On the southern side, the bedrock flank of the Belpberg mountain marks the limit of the prism (Figure D.7.4). An additional prism was placed on the NE flank to model the occurrence of a presumably shallow sedimentary layer. Its limited extend to the NW is justified because the bedrock topography map of Reber and Schlunegger (2016) points towards the occurrence of a bedrock knob in this area.

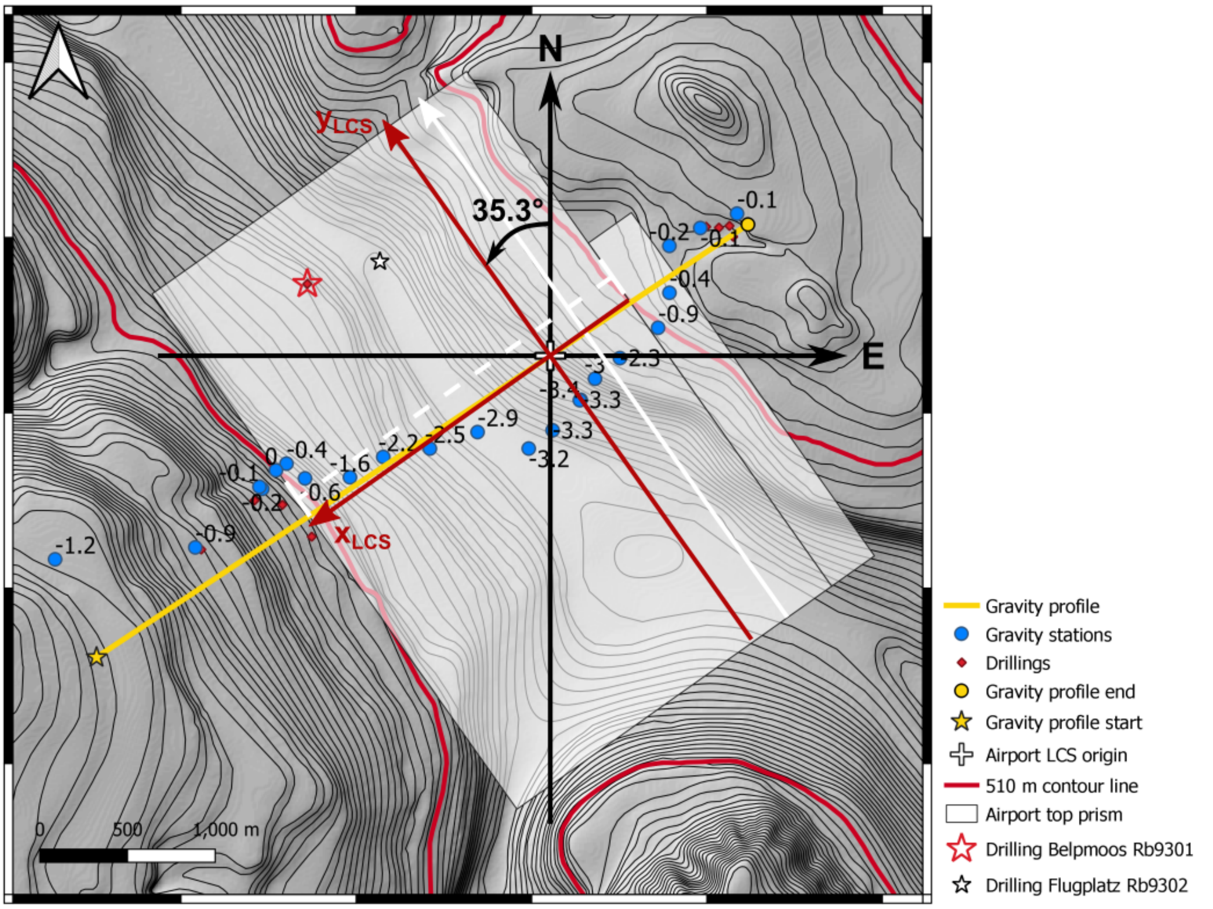

Figure D.7.4 *Map of the bedrock in the region of the Airport profile (Reber and Schlunegger 2016) and estimated geometries of top prisms for the initial model. The yellow line indicates the location of the gravity profile, the blue dots show the gravity stations with their residual anomaly values and the red dots are drillings that reached the bedrock. The white and red stars locate deep drillings far from the profile; the white star is a drilling that did not reach the bedrock. The red x- and y-axes indicate the LCS used for the modelling. The LCS y-axis is oriented parallel to the tunnel valley main direction, indicated by the white line (see Figure 7b). The direction of the LCS x-axis (red arrow) parallels the white broken line and is oriented perpendicularly to the direction of the overdeepening. Both axes are crossing the LCS origin, marked by the white plus sign. This location denotes the projection of the gravity station with the maximum amplitude onto the profile. Note that the LCS x-axis is not exactly parallel to the gravity profile. There is a small angle between them as discussed in the figures below. The LCS y-axis is obtained by a counterclockwise rotation of 35.3° from the North that denotes the y-axis of the Swiss coordinates system (black solid lines). The white rectangle indicates the dimensions of the top prism used to model the overdeepening infill for this profile. The dimensions of the main prism are 2.3 km in width and 3.6 km in length along the valley axis (1.6 km towards the NW and 2 km towards the SE). The dimensions of the side prism are 0.3 km in width and 2.4 km in length (0.4 km toward the NW and 2km toward the SE).*

Conducting the modelling

The initial model consists of 4 prisms. These include the side prism shown on Figure D.7.5 and three large prisms used to represent the main volume of the overdeepening. The initial geometry introduces a slight asymmetry, which is done to match the shape of the residual anomalies that has a steeper NE flank and a flatter SW side. The total thickness of 200 m of the three prisms is given by the drilling information. Although the corresponding drilling (white star on results plots below) is located far away from the profile, we consider its depth information as a minimum constraint for the elevation of the main overdeepening's base. The initial density contrast of -500 kg/m^3 between the Molasse bedrock and the overdeepening fill is taken from Bandou et al. (2022). We used the value of these authors because similar to the aforementioned study, the overdeepening fill of the Airport profile is underlain by the Upper Marine Molasse bedrock. In addition, the Bandou et al. (2022) profile through the Aare valley is quite close to the Airport profile (see Figure D.1).

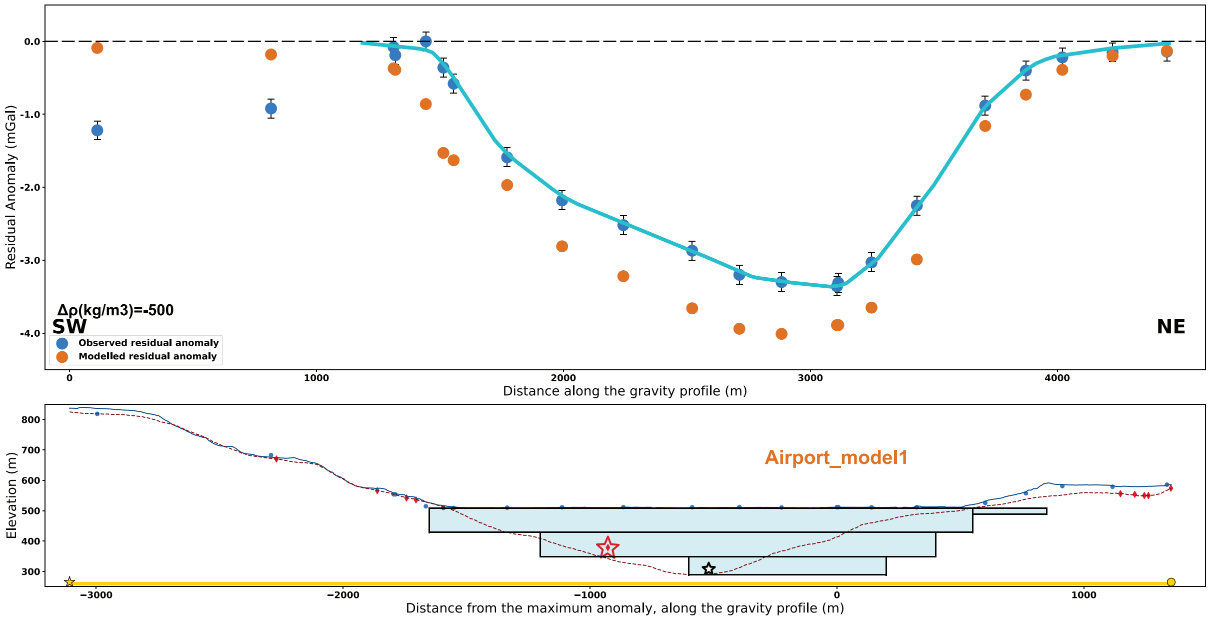

Figure D.7.5 *Initial model1 for the Airport gravity profile, made with 4 prisms with a uniform density contrast of -500 kg/m*^{3}*between the Molasse bedrock and the overdeepening fill. Top: The blue dots represent the observed residual anomaly, and the orange dots are the calculated gravity values for model 1. The black bars indicate our maximum uncertainty of +/- 0.13 mGal. The light blue line highlights the main anomaly. Bottom: Elevation (SwissAlti3D 2m DEM (© swisstopo)) along the profile (blue solid line). The red broken line illustrates the bedrock topography of the model Reber and Schlunegger (2016). The blue dots are the gravity stations, the red diamonds indicate drillings that reached the bedrock. The yellow star and point indicate the start and end of the gravity profile, respectively. The stars show drillings located > 900 m away from the profile, the red one for the Rb9201 drilling while the white one shows the Rb9202 drilling that did not reach the bedrock. The black rectangles show the cross sections of the 4 prisms used for modelling. Modelling conclusions: The overall shape of the overdeepening effect is fairly well reproduced, but the model overestimates the observed anomaly in the wavelength and the amplitude. The position of the modelled maximum anomaly position is located too far to the SW.*

The results of the initial model match the residual anomaly asymmetry quite well (Figure D.7.5). However, the modelled gravity signals extend too far on both flanks and the modelled maximum amplitude obviously exceeds the measured maximum amplitude. These differences between calculated and measured gravity imply that on the SW side the overdeepening flank must be shifted farther to the NE and on the NE flank, both the extent and the thickness of the side prism may be reduced. The following models therefore focused on improving the fit by adjusting the top part of the model. First the width of the top prism was reduced in order to adjust the model wavelength to the observations. Then the top prisms were split so that we could better fine-tune their geometries, thereby reducing the overestimates of the effects on both flanks.
By two iterations we obtain model 3 (Figure D.7.6) that shows the upper geometry of the through has been reproduced, as the modelled and observed residual anomalies perfectly fit. The NE flank also shows an overall perfect fit between model results and observations. The location where the model results overestimate the gravity effect of the overdeepening fill on the SW side is situated where the slope angle of the residual anomalies decreases in the middle of the flank. This indicates that the prism 4 (numbered from top) in model 3 is too thick and extends too far towards the SW. For the subsequent models we therefore split the prism 4 into a thinner upper prism and a shorter but thicker lower prism to properly model the maximum anomaly.

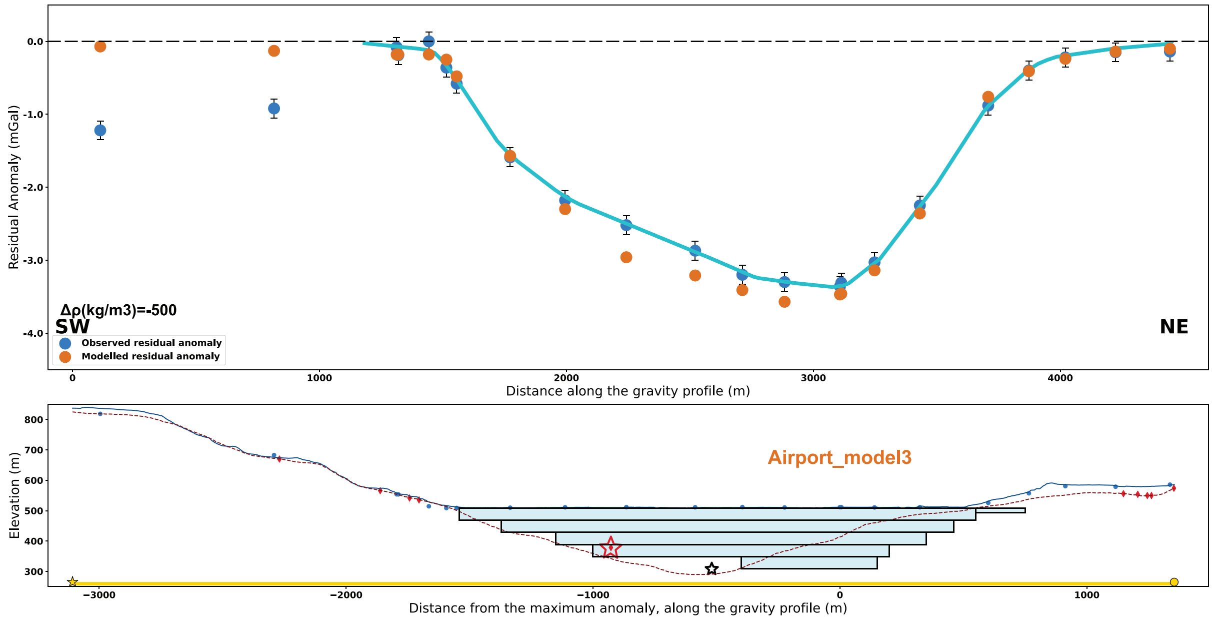

Figure D.7.6 *Model 3 of Airport profile with 6 prisms that exhibit a uniform density contrast of -500kg/m³ relative to the bedrock. Top: The blue dots represent the observed residual anomaly, and the orange dots are the calculated gravity values for model 3. The black bars indicate our maximum uncertainty of +/- 0.13 mGal. The light blue line highlights the main anomaly. Bottom: Elevation (SwissAlti3D 2m DEM (© swisstopo)) along the profile (blue solid line). The red broken line illustrates the bedrock topography of the Reber and Schlunegger (2016) model. The blue dots are the gravity stations, the red diamonds indicate drillings that have reached the bedrock. The stars show drillings located > 900 m away from the profile; the red one for the Rb9201 drilling while the white one shows the Rb9202 drilling that did not reach the bedrock. The black rectangles show the cross sections of the 6 prisms of model 3. Modelling conclusions: Using twice the number of prisms (in comparison to model 1), the observed and modelled gravity wavelengths now properly fit. The same fit between model results and observations is reached for the whole NE flank. On the lower part of the SW flank though, the gravity effect of the overdeepening fill is still overestimated. The location of the modelled maximum anomaly is still slightly too far to the SW.*

Two possible final geometries

So far, the modelling of the Airport profile was done with a uniform density contrast between the bedrock and overdeepening fill of -500 kg/m³ for all prisms. This solution (Figure D.7.7a) allowed us to reproduce the bedrock depth as encountered by the Rb9201 drilling (red star, albeit with a large distance). But we were not able to reach the deeper depth as inferred by the Rb9202 (white star) drilling, even if the model does reproduce its depth of 200m (yet, this drilling did not reach the bedrock). Subsequent modelling (models 5 to 7) with a variable density contrast, however, revealed that even a slightly better fit between modelled and observed gravity is possible by using a different density contrast of -350 kg/m³ for the lower three prisms (Figure 7.7.7b). This alternative solution for the geometry of the lower part of the Airport profile also correlates with the depths as constrained by the Rb9202 drilling.

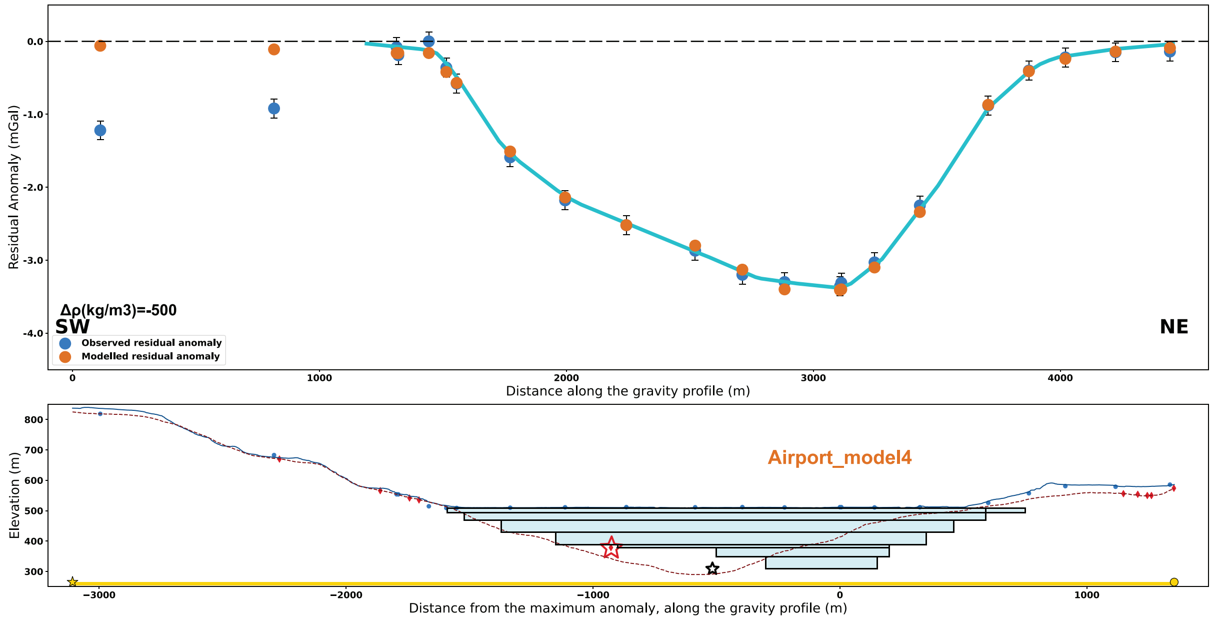

Figure D.7.7a *Final model for the Airport profile. Model 4 was conducted with a total of 8 prisms with a density contrast of -500 kg/m³ assigned to all prisms. Top: The blue dots represent the observed residual anomaly, and the orange dots are the modelled residual anomaly values for model 4. The black bars indicate our maximum uncertainty of +/- 0.13 mGal. The light blue line highlights the main anomaly. Bottom: Elevation (SwissAlti3D 2m DEM (© swisstopo)) along the profile (blue solid line). The red broken line illustrates the bedrock topography of the model Reber and Schlunegger (2016). The blue dots are the gravity stations, the red diamonds indicate drillings that have reached the bedrock. The stars show drillings located > 900 m away from the profile; the red one for the Rb9201 drilling while the white one shows the Rb9202 drilling that did not reach the bedrock. The black rectangle shows the cross sections of the 7 prisms used for modelling. Modelling conclusions: The observed main anomaly is very well fit on both flanks by the model results. The remaining differences between the observed and modelled gravity are all within the limits of the maximum uncertainty estimates. The deep geometry of this model though does only coincide with one of the two deep drillholes information (see text). The maximum thickness assigned to the overdeepening fill in this model 4 is 200 m. The modelled geometry shows a strong asymmetry between the SW and NE flanks, with a steep uniform NE flank and a SW flank that is less steep. The model output finally returns a c. 1 km-long plateau. The bottom of the overdeepening is wide and flat, suggesting an overall asymmetrical U-shaped geometry.*

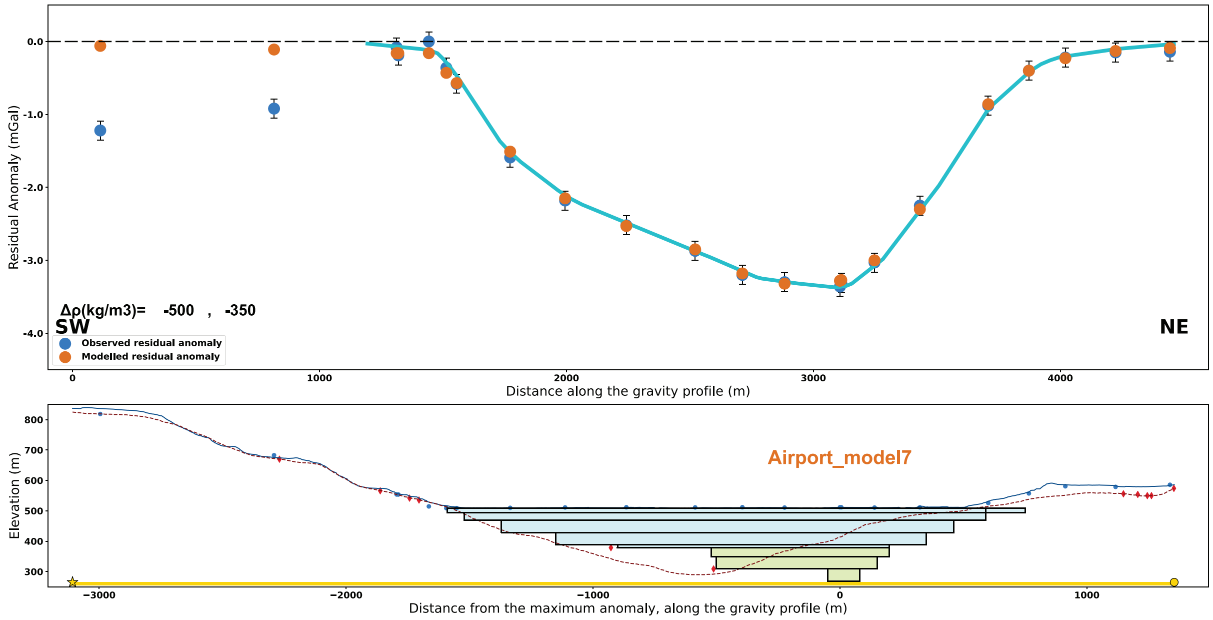

Figure D.7.7b *Final model for the Airport profile, model 7 made with a total of 9 prisms with a density contrast of -500 kg/m³ for the top six prisms (light blue) and of -350 kg/m³ for the bottom three prisms (light green). Top: The blue dots represent the observed residual anomaly, and the orange dots are the modelled residual anomaly*

values for model 7. The black bars indicate our maximum uncertainty of +/- 0.13 mGal. The light blue line highlights the main anomaly. Bottom: Elevation (SwissAlti3D 2m DEM (© swisstopo)) along the profile (blue solid line). The red broken line illustrates the bedrock topography of the model by Reber and Schlunegger (2016). The blue dots are the gravity stations, the red diamonds indicate drillings that have reached the bedrock. The stars show drillings located over 900 m away from the profile: the red one for the Rb9201 drilling while the white one shows the Rb9202 drilling that did not reach the bedrock. The black rectangle shows the cross sections of the 9 prisms used for modelling. The prisms in light blue have a density of -500 kg/m^3 with those in light green have one of -350 kg/m^3. Modelling conclusions: This final geometry solution follows the same conclusions as model 4, with even a slightly better overall fit between the modelled gravity effect and observations due to the different density contrasts assigned to the prisms upon modelling. The geometries are identical for the 6 prisms above the plateau. The main difference is the geometry of the deeper section. In this model 7, the modelled geometry matches the depth of the Rb9202 drilling. The model output is characterized by a wider and flat lower section, and a narrow deepest part. This would indicate the occurrence of a trough dominated by a U-shaped geometry with a more V-shaped geometry for the deepest section.

The final models 4 and 7 (Figure D.7.7) show the same geometry for the upper section of the overdeepening pointing towards an asymmetrical U-shaped geometry for the bedrock topography underneath the overdeepening fill. However, both models differ regarding the lower sections. Whereas model 4 infers the occurrence of a flat and wide U-shaped deeper part, model 7 returns a geometry where the bedrock shape is narrow and V-shaped in the deepest part. Note that these models are constrained by a drilling located very far from the profile. Therefore, the drillholes information might not be exactly representative for the geometry of the valley along the profile where our gravity stations were placed. The geometry of both models is as likely as the other, considering that the geological data, the drilling information available for the Airport profile cannot favour one over the other.

D.8 The Bern3, Bern4, Wabern1 and Wabern2 profiles

The residual anomaly values and the related profiles were determined with the identical logic as for the other profiles. We therefore refer the reader to the sections above for details. Here, we solely document the location of the Bern3 (Figure D.8.1), the Bern4 (Figure D.8.2), the Wabern1 (Figure D.8.3) and the Wabern2 (Figure D.8.4) profiles.

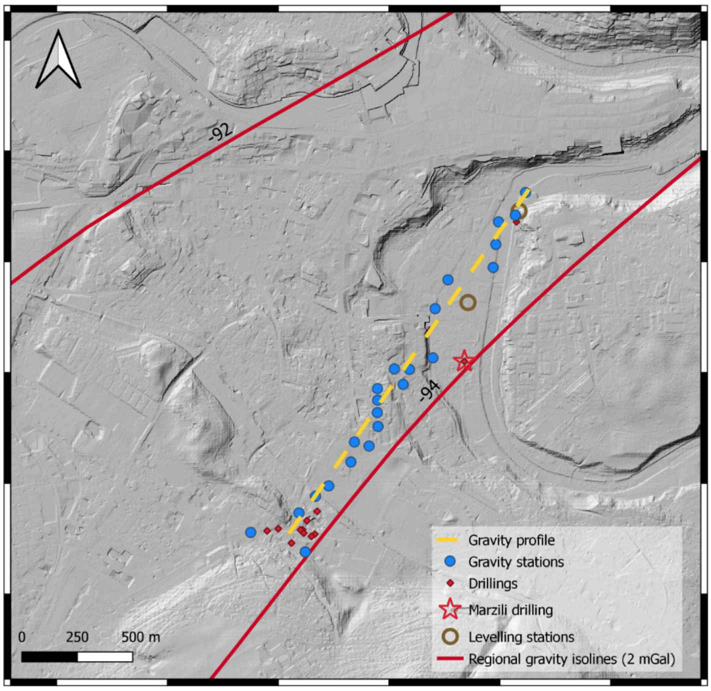

Figure D.7.1 *Enlarged extraction of regional Bouguer gravity map showing the vicinity of the Wabern1 gravity profile (yellow line). The blue dots are the newly measured gravity stations. The red diamonds show the location of the drillings that reached the Molasse bedrock. The red star highlights the location of the Marzili drilling.*

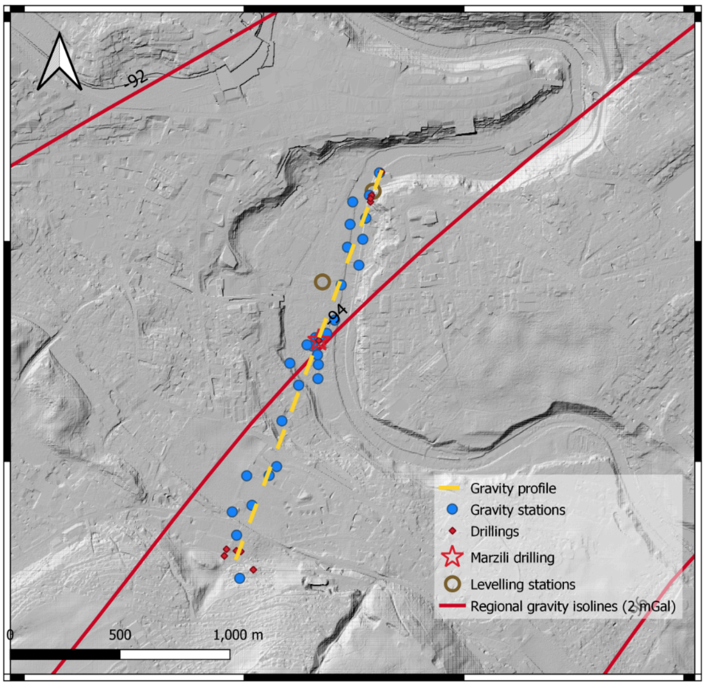

Figure D.7.2 *Enlarged extraction of regional Bouguer gravity map showing the vicinity of the Bern4 gravity profile (yellow line). The blue dots are the newly measured gravity stations. The red diamonds show the location of the drillings that reached the Molasse bedrock. The red star highlights the location of the Marzili drilling.*

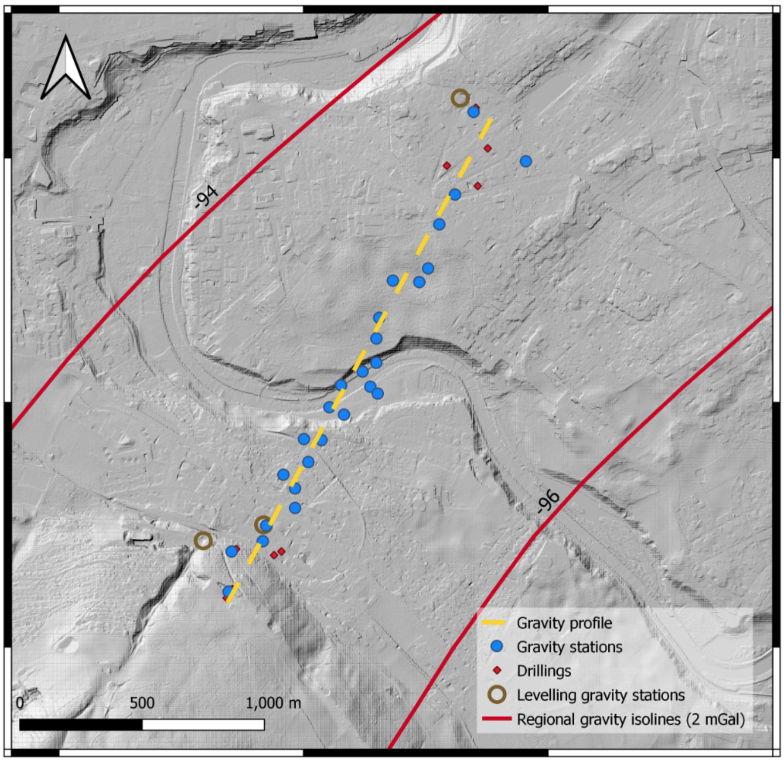

Figure D.7.3 *Enlarged extraction of regional Bouguer gravity map showing the vicinity of the Wabern1 gravity profile (yellow line). The blue dots are the newly measured gravity stations. The red diamonds show the location of the drillings that reached the Molasse bedrock.*

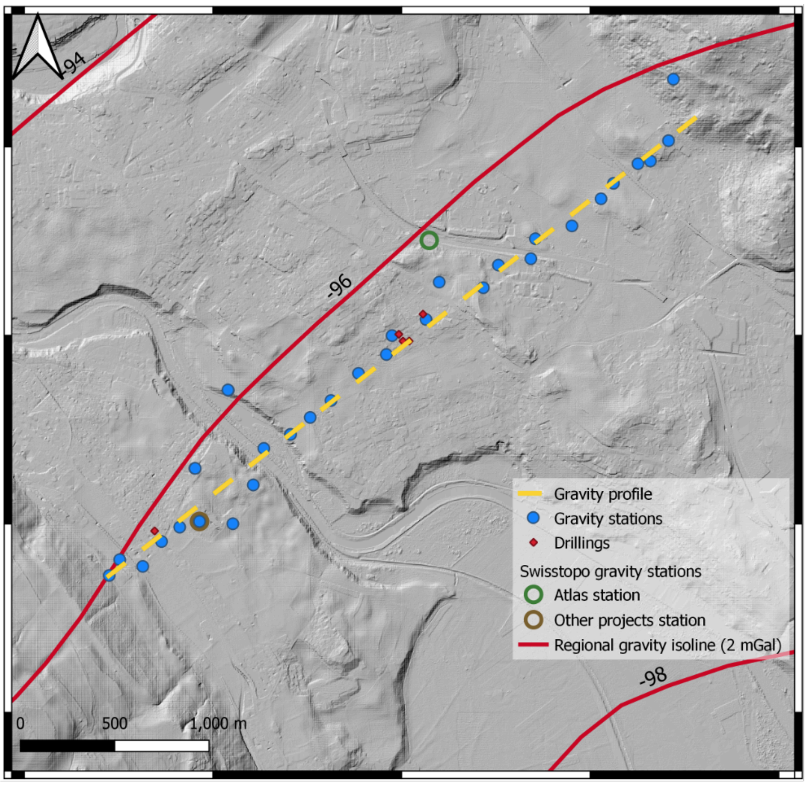

Figure D.7.4 *Enlarged extraction of regional Bouguer gravity map showing the vicinity of the Wabern2 gravity profile (yellow line). The blue dots are the newly measured gravity stations. The red diamonds show the location of the drillings that reached the Molasse bedrock.*
